# Non-invasive characterization of perivascular subarachnoid spaces

**DOI:** 10.1038/s41467-026-76306-9

**Published:** 2026-08-05

**Authors:** Nina E. Fultz, Geir Ringstad, Madda Debiasi, Emiel C. A. Roefs, Siri Fløgstad Svensson, Per Kristian Eide, Marianne A. A. van Walderveen, Jeroen de Bresser, Matthias J. P. van Osch, Lydiane Hirschler

**Affiliations:** 1 C.J. Gorter MRI Center, Department of Radiology, Leiden University Medical Center, Leiden, Netherlands; 2 Institute of Clinical Medicine, Faculty of Medicine, University of Oslo, Oslo, Norway; 3 K.G. Jebsen Centre for Brain Fluid Research, University of Oslo, Oslo, Norway; 4 Department of Radiology, Oslo University Hospital – Rikshospitalet, Oslo, Norway; 5 Department of Physics and Computational Radiology, Oslo University Hospital, Oslo, Norway; 6 Department of Neurosurgery, Oslo University Hospital – Rikshospitalet, Oslo, Norway

**Keywords:** Neuroscience, Anatomy

## Abstract

Cerebrospinal fluid (CSF) is thought to facilitate brain waste clearance and immune surveillance, yet its compartmentalization remains unclear. Previous work, using invasive dynamic intrathecal MRI contrast imaging, identified a perivascular subarachnoid space (PVSAS) that enhances along the major cerebral arteries with a ‘donut’-like appearance. These findings suggest that the PVSAS may be separated from the surrounding broader subarachnoid space (SAS) by a semipermeable perivascular membrane. To investigate if the PVSAS could be observed non-invasively in healthy controls, we used a magnetic resonance imaging technique, CSF-STREAM (CSF-Selective T_2_-prepared REadout with Acceleration and Mobility-encoding), that assesses CSF-mobility at a high spatial resolution by isolating CSF from blood and tissue signal. Here, we observe high CSF-mobility next to the vasculature, with a steep drop-off into the surrounding SAS around both the middle and anterior cerebral arteries, suggesting the presence of the PVSAS in healthy controls. We find that CSF dynamics may be more spatially distinct than previously thought, providing a possible foundation for understanding brain CSF patterns in health and disease.

## Abstract (web summary)

Cerebrospinal fluid’s compartmentalization remains unclear. Here, the authors show increased CSF-mobility around major cerebral arteries within the surrounding subarachnoid space, suggesting the presence of distinct CSF spaces in healthy individuals.

## Introduction

Converging evidence suggests that cerebrospinal fluid (CSF) plays a central role in brain homeostasis^1–4^, functioning as a pathway for nutrient delivery^5,6^, immunosurveillance^7^, and metabolic waste clearance^8,9^. However, recent research challenges the traditional view^10^ of the subarachnoid space (SAS), which harbors the CSF, as a single continuous space^11,12^. For example, dynamic imaging after intrathecal injection of an MRI contrast agent shows a perivascular subarachnoid space (PVSAS) that enhances along the major cerebral arteries with a ‘donut’-like appearance^10^. This suggests that CSF flows from the basilar cisterns along the anterior cerebral artery (ACA) and the middle cerebral artery (MCA). The flow appears to occur within a narrow PVSAS adjacent to the vasculature, potentially separated from the broader, outer SAS by a semipermeable perivascular membrane.

However, these observations were made in patients undergoing intrathecal contrast agent injections as part of their diagnostic work-up, i.e. with suspicion of a CSF disorder. In patients with idiopathic normal pressure hydrocephalus, the PVSAS were significantly enlarged, and CSF transport within the PVSAS was impaired^10,13^. It is unknown whether the PVSAS can also be detected in healthy individuals and whether it can be observed non-invasively. To investigate this, we employed a magnetic resonance neuroimaging technique, called CSF-STREAM (CSF-Selective T_2_-prepared REadout with Acceleration and Mobility-encoding)^14^, that assesses high resolution (0.45 mm isotropic) CSF-mobility. Importantly, this MRI-technique isolates CSF by suppressing blood and tissue signal, thereby ensuring the measured motion solely reflects CSF dynamics and not slow blood flow.

Based on the previous intrathecal contrast agent work, we hypothesize that the CSF-mobility maps would share a high level of similarity in their spatial patterns to early timepoint intrathecal contrast-enhanced scans. Because the intrathecal contrast traveled fastest along the PVSAS, we expect PVSAS in healthy controls to exhibit higher CSF-mobility around branches of the MCA and ACA (M1-M4, A1-A4) compared with the adjacent SAS.

In this work, we first compare CSF-STREAM CSF-mobility maps in healthy controls to intrathecal contrast-enhanced scans from reference patients. Given that post-M1 (defined as M2-M4) arteries showed the highest incidence of PVSAS in the intrathecal scans, we then examine the characteristics of CSF-mobility around the post-M1 arteries, and then the CSF-mobility around the M1, ACA, and ICA arteries. Specifically, we hypothesize that CSF-mobility would be significantly higher within the PVSAS than in the surrounding SAS. We further hypothesize that CSF-mobility principal orientation will be more homogeneous within the PVSAS compared to the SAS, reflecting an anatomical constraint, and that transitions at the ICA bifurcation would mark the emergence of distinct PVSAS compartments. Motivated by recent reports of arachnoid cuff exit (ACE) points^7^, we then examine arteries and veins adjacent to the superior sagittal sinus (SSS) for PVSAS CSF-mobility patterns. Finally, we examine possible drivers of the CSF-mobility by looking at how cardiac and respiration dynamics modulate CSF-mobility within the PVSAS and SAS.

## Results

### CSF-mobility and contrast-enhanced scans show similar PVSAS

**Fig. 1:**
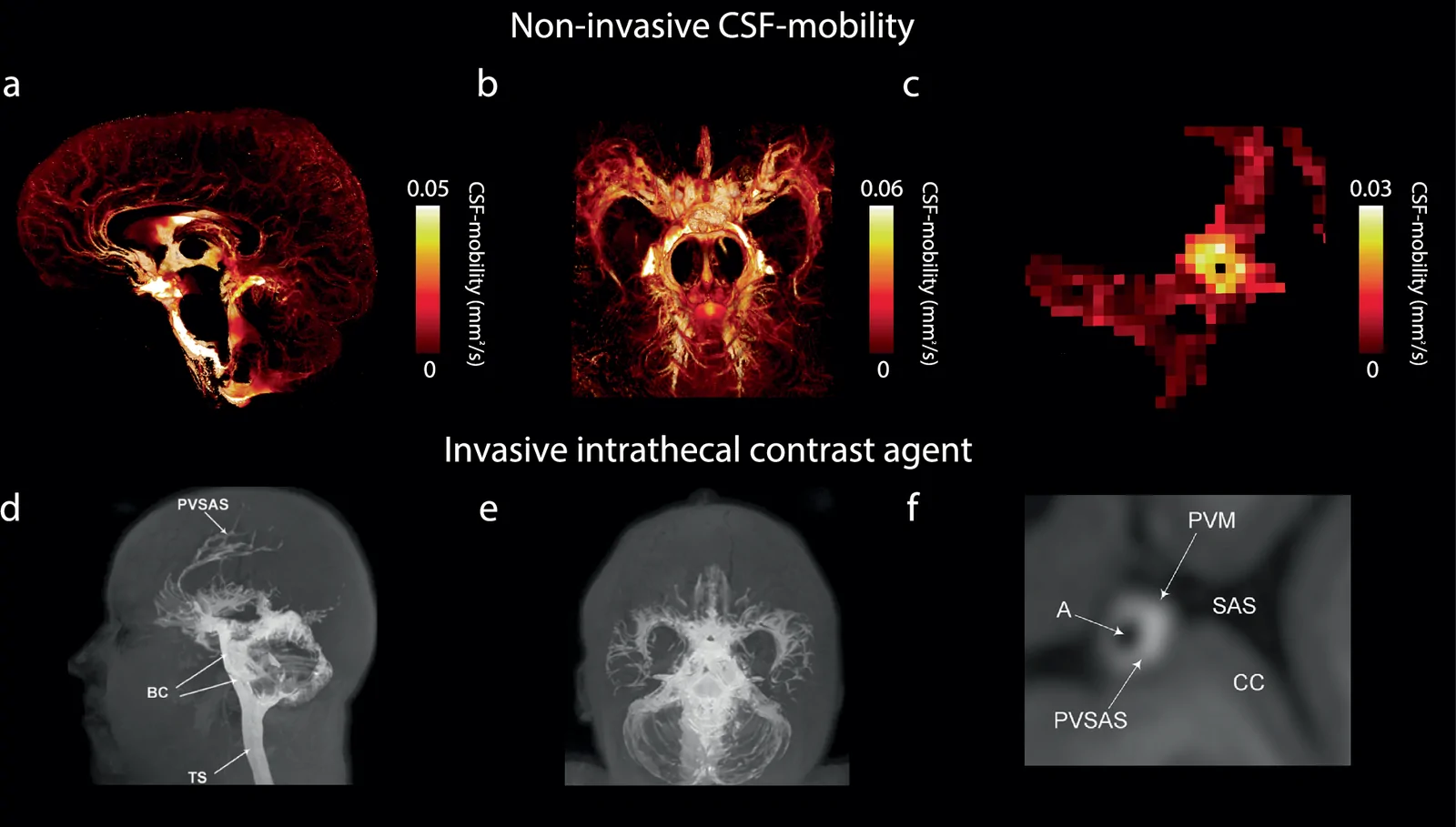
Comparison of 3D CSF-mobility and intrathecal contrast scans. **a** Sagittal view of a 7 Tesla CSF-mobility scan in a healthy control shows perivascular subarachnoid space (PVSAS) surrounding the anterior cerebral artery (ACA). **b** Transverse view of a CSF-mobility scan reveals high CSF-mobility PVSAS around the middle cerebral artery (MCA). **c** Zoomed-in example of a high CSF-mobility PVSAS surrounding an artery in a region of otherwise low CSF-mobility near the superior sagittal sinus (SSS). **d**–**f** Corresponding intrathecal contrast agent scans from reference patients: sagittal (**d**), transverse (**e**), and a zoomed-in PVSAS example (**f**), showing similar spatial organization to CSF-mobility scans. Contrast is concentrated near the vessel, within a larger subarachnoid space (SAS). ‘A’ indicates the artery; ‘PVM’ denotes the perivascular membrane; ‘CC’ shows the cerebral cortex; versions of these images have been presented in previous work^10^.

First, we examined whether CSF-mobility maps reflect similar spatial patterns to those observed in intrathecal contrast-enhanced scans that delineate the PVSAS. In eleven healthy controls, whole-brain comparisons revealed that CSF-mobility patterns closely resembled those reported in the intrathecal scans^10^. In intrathecal CSF scans, sagittal views show a prominent signal in the basilar cisterns, reflecting rapid contrast transport. A similar pattern is observed in the CSF-mobility scans, which, without using contrast, show elevated CSF-mobility in the basilar cisterns (Fig. 1a, b, d, e, Supplemental Figs. 2, 4c, g, 9a–f).

**Fig. 2:**
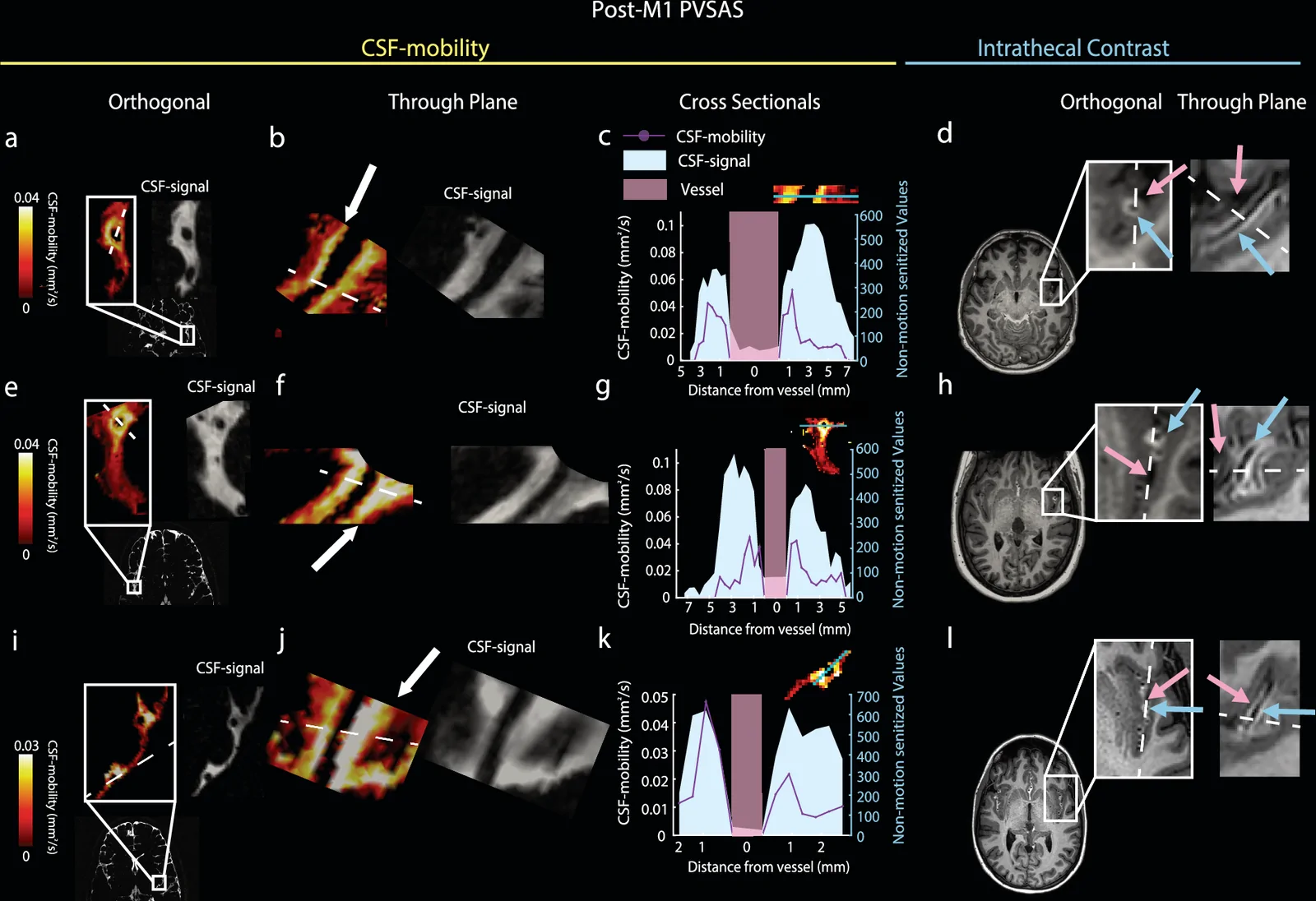
Perivascular subarachnoid space (PVSAS) in the post-M1 (M2-M4) region. **a**, **e**, **i** The *first column* shows full-donut PVSAS with high CSF-mobility surrounding post-M1 arteries. Non-motion-sensitized scans (gray) (referred to as ‘CSF-signal’) confirm equal CSF presence in both high- and low-mobility regions. Some of the PVSAS show some structural heterogeneity, indicating that the full-donut PVSAS can be asymmetric and deviate from perfect circularity (e.g., (**e**, **i**)). White boxes mark regions used for PVSAS extraction; dotted lines indicate locations of the corresponding through-plane images in the second column. **b**, **f**, **j** *Second column* shows through-plane views of orthogonal CSF from the first column, displaying CSF-mobility, alongside matching non-motion-sensitized scans (gray). Dotted lines indicate locations of the corresponding orthogonal images in the first column. White arrows show a sharp drop-off. **c**, **g**, **k** The *third column* presents representative cross-sectional plots from similar areas as in the first and second columns. The pink bar marks the vessel; the plots show CSF-mobility (purple, left y-axis) and non-motion-sensitized signal (blue, right y-axis) as a function of the distance to the vessel (in mm) on the x-axis. ROI used are indicated in blue on the CSF-mobility map in the top right of the plot. Source data are provided as a Source Data file. **d**, **h**, **l** *Fourth column* shows post-M1 intrathecal contrast-enhanced PVSAS from patients from similar anatomical locations as in the first and second columns (examples are from data used in Eide & Ringstad, 2024). Dotted white lines indicate intersecting locations. Pink arrow highlights the subarachnoid space (SAS), and the blue arrows show the contrast within the PVSAS.

**Fig. 3:**
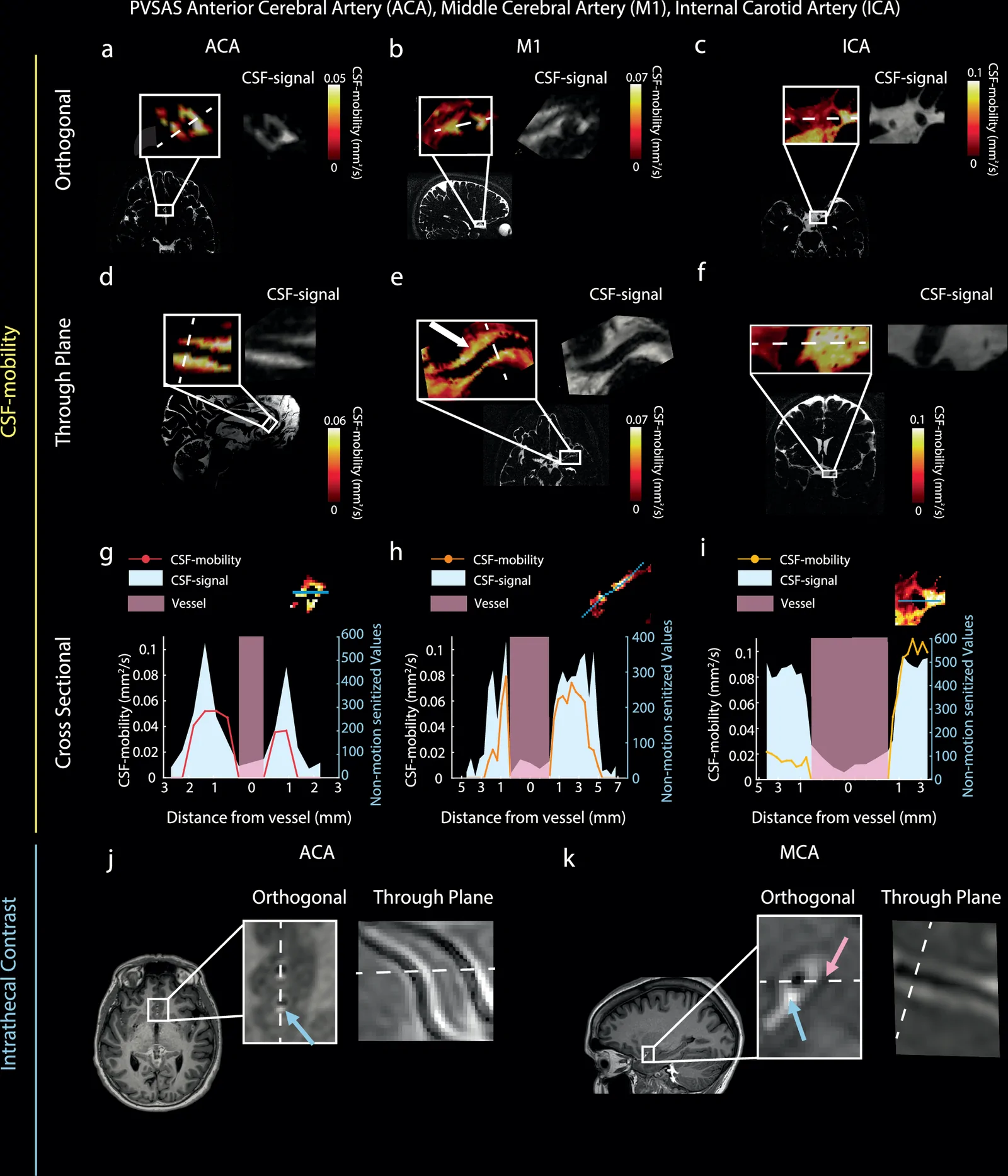
Perivascular subarachnoid space (PVSAS) across the anterior cerebral artery (ACA), M1 and internal carotid artery (ICA). **a** ACA CSF-mobility PVSAS, displaying tight-CSF in the orthogonal image. The non-motion-sensitized scan (gray) (referred to as ‘CSF-signal’) shows a narrow CSF space around the vessel. The white box marks the extraction site of PVSAS; the white dotted line indicates the location of the through-plane image in (**d**). **b** M1 CSF-mobility PVSAS showing high CSF-mobility near the artery in a bilateral triangular pattern within a region of lower CSF-mobility. The non-motion-sensitized scan (gray) indicates that reduced mobility is not due to a lower CSF-signal. Identical layout to ‘a’. **c** ICA CSF-mobility two-compartment, showing asymmetric mobility: low CSF-mobility on one side of the artery and high on the other. The non-motion-sensitized scan (gray) confirms a similar CSF-signal on both sides of the artery. The white box indicates the extracted region for the through-plane image. **d**–**f** Through-plane images of ACA, M1, and ICA CSF-mobility. The non-motion-sensitized scan (gray) shows CSF-signal. The white dotted line shows the location of the orthogonal cut from (**a**–**c**). **g**–**i** Representative cross-sectional plots of the PVSAS are shown in a similar area to the orthogonal cut for ACA, M1, and ICA above. Pink bar represents vessel; the x-axis indicates distance from vessel (mm), the left y-axis shows CSF-mobility, and the right y-axis shows non-motion-sensitized signal. ROI location shown in the corner in blue. Source data are provided as a Source Data file. **j** ACA (A1) intrathecal contrast scan showing tight contrast-enhancement^10^ near the vessel (blue arrow), and can be compared to the ACA PVSAS in (**a**, **d**). The white dotted line indicates orthogonal and through-plane images. **k** MCA intrathecal contrast scan shows high eye-shaped contrast-enhancement^10^ next to the vessel (blue arrow), within a lower contrast area (pink arrow), and can be compared to M1 PVSAS in (**b**, **e**). The white dotted line shows orthogonal and through-plane images.

Ascending the vascular tree, intrathecal contrast enhancement around the ACA is mirrored by increased CSF-mobility. Transverse views show that both intrathecal contrast and high CSF-mobility trace the trajectory of the MCA branches from M1 to M4 (Figs. 1b, e, 2, 3, Supplemental Figs. 2e–h, 4a, b, e, f, 9d–i). Additionally, when examining the whole brain CSF-mobility images, we found high CSF-mobility staying in a confined space around vessels within the SAS (Fig. 1c) paralleled in the intrathecal imaging (Fig. 1f). Areas with low contrast enhancement, such as the SAS at the brain surface, showed low CSF-mobility overall.

### CSF-mobility around arteries shows distinct PVSAS characteristics

Given that Eide and Ringstad^10^ observed the PVSAS most prominently around arteries distal to M1, we next examined the characteristics of CSF-mobility surrounding post-M1 vessels. In orthogonal views to these arteries, we consistently observed regions of high CSF-mobility immediately adjacent to the vessel wall, with a sharp drop-off into surrounding CSF-pools with low mobility (Fig. 2a, e, i, Supplemental Figs. 3a, 4f, 9g–i). In through-plane views along the vessel, the same high-mobility regions ran along the artery within a broader area of lower CSF-mobility (Fig. 2b, f, j).

**Fig. 4:**
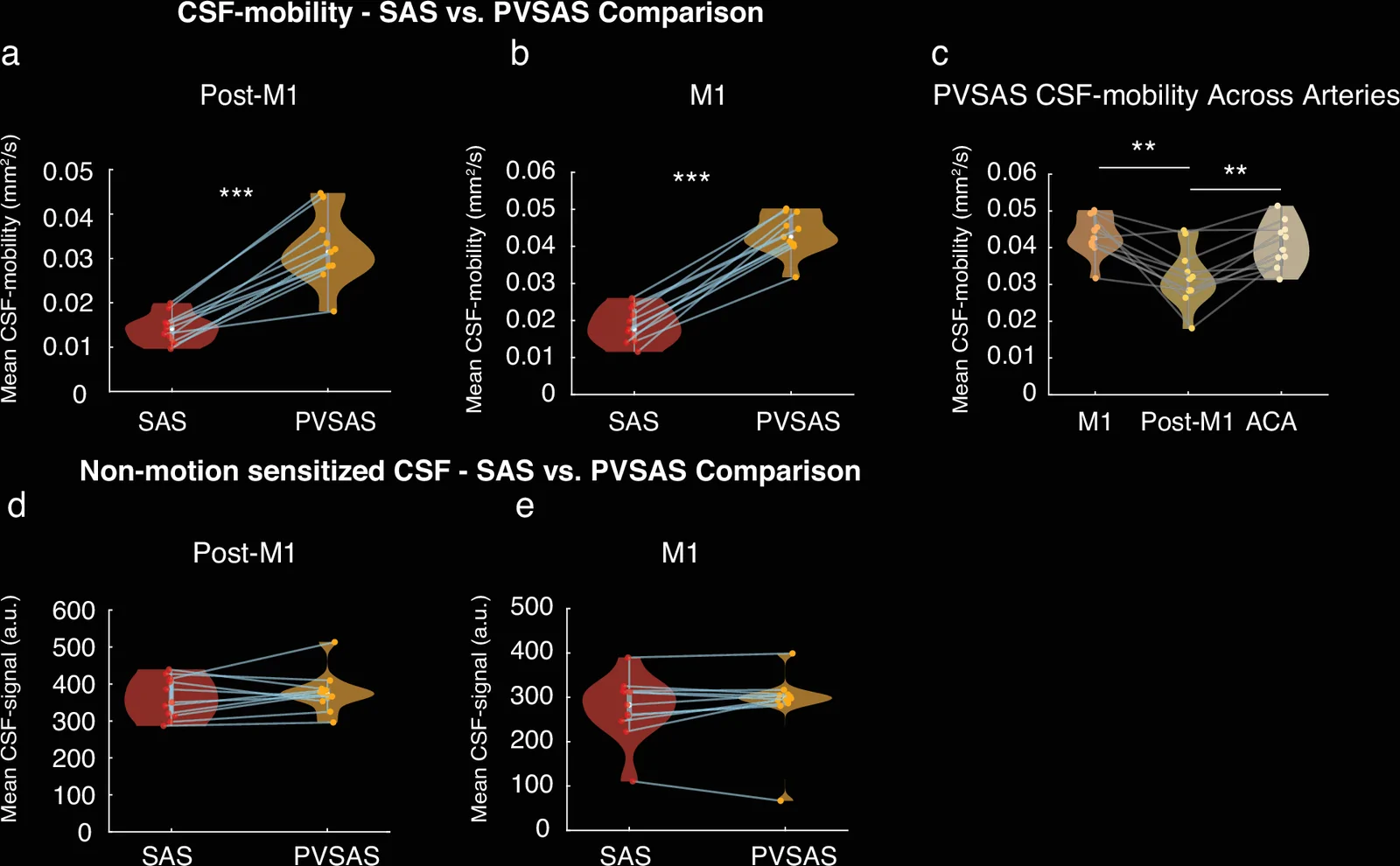
Elevated CSF-mobility in the perivascular subarachnoid space (PVSAS) relative to the subarachnoid space (SAS). **a** Violin plot of post-M1 mean CSF-mobility in two subsections of the SAS and PVSAS (post-M1 PVSAS mean = 0.03 ± 0.008 mm^2^/s, post-M1 SAS mean = 0.01 ± 0.003 mm^2^/s; *n* = 21 segments, 11 subjects, *p* = 2.0 × 10^−6^, two-sided paired *t*-test, Bonferroni-corrected, 2 tests). **b** Violin plot of M1 mean CSF-mobility in two subsections of PVSAS and SAS (M1 PVSAS mean = 0.04 ± 0.005 mm^2^/s, post-M1 SAS mean = 0.02 ± 0.005 mm^2^/s; 21 segments, 11 subjects, *p* = 6.5 × 10^−8^, two-sided paired *t*-test, Bonferroni-corrected, 2 tests). **c** PVSAS CSF-mobility values across M1, post-M1, and ACA, shows significantly more CSF-mobility in the M1 and ACA PVSAS than the post-M1 PVSAS (11 subjects, M1 vs. post-M1: *p* = 1.49 × 10^−3^; M1 vs. ACA: *p* = 0.19; post-M1 vs. ACA: *p* = 1.12 × 10^−3^, two-sided paired *t*-test, Bonferroni-corrected, 3 tests). **d**, **e** SAS and PVSAS ROIs of the post-M1 and M1 on non-motion-sensitized images show similar CSF signal intensity, indicating that differences in CSF-mobility between SAS and PVSAS are not due to a change in CSF presence (11 subjects, post-M1 *p* = 0.32, M1 *p* = 0.32, two-sided paired *t*-test). All source data for this figure are provided as a Source Data file.

Post-M1 cross-sectional PVSAS plots show a steep CSF-mobility drop-off from ~0.04 mm²/s to ~0.01 mm²/s in the surrounding SAS (Fig. 2c, g, k). Overall, mean post-M1 CSF-mobility is significantly lower in the SAS compared to the PVSAS (Fig. 4a; post-M1 PVSAS mean = 0.03 ± 0.008 mm^2^/s, post-M1 SAS mean = 0.01 ± 0.003 mm^2^/s; 21 segments, 11 subjects, *p* = 2.0 × 10^−6^, paired *t*-test, Bonferroni-corrected, 2 tests). The non-motion-sensitized scans show no significant change in the post-M1 PVSAS vs SAS (Fig. 4d; 21 segments, 11 subjects, post-M1 *p* = 0.32, paired *t*-test). Semi-automated concentric circles around the post-M1 vasculature mirror these results, showing high CSF-mobility with a steep drop-off at ~1.5 mm distance from the vessel wall (Supplemental Fig. 7f; 11 subjects, post-M1 *p* = 6.0 × 10^−4^, paired *t*-test, Bonferroni-corrected, 2 tests; see also Supplemental Figs. 7e, 8). Mean non-motion-sensitized CSF-signal show no significant change between where the PVSAS is expected (0–1.5 mm) and the SAS (1.5–3 mm) (Supplemental Fig. 7h; 11 subjects, post-M1 *p* = 0.74, paired *t*-test; see also Supplemental Figs. 7g, 8).

**Fig. 5:**
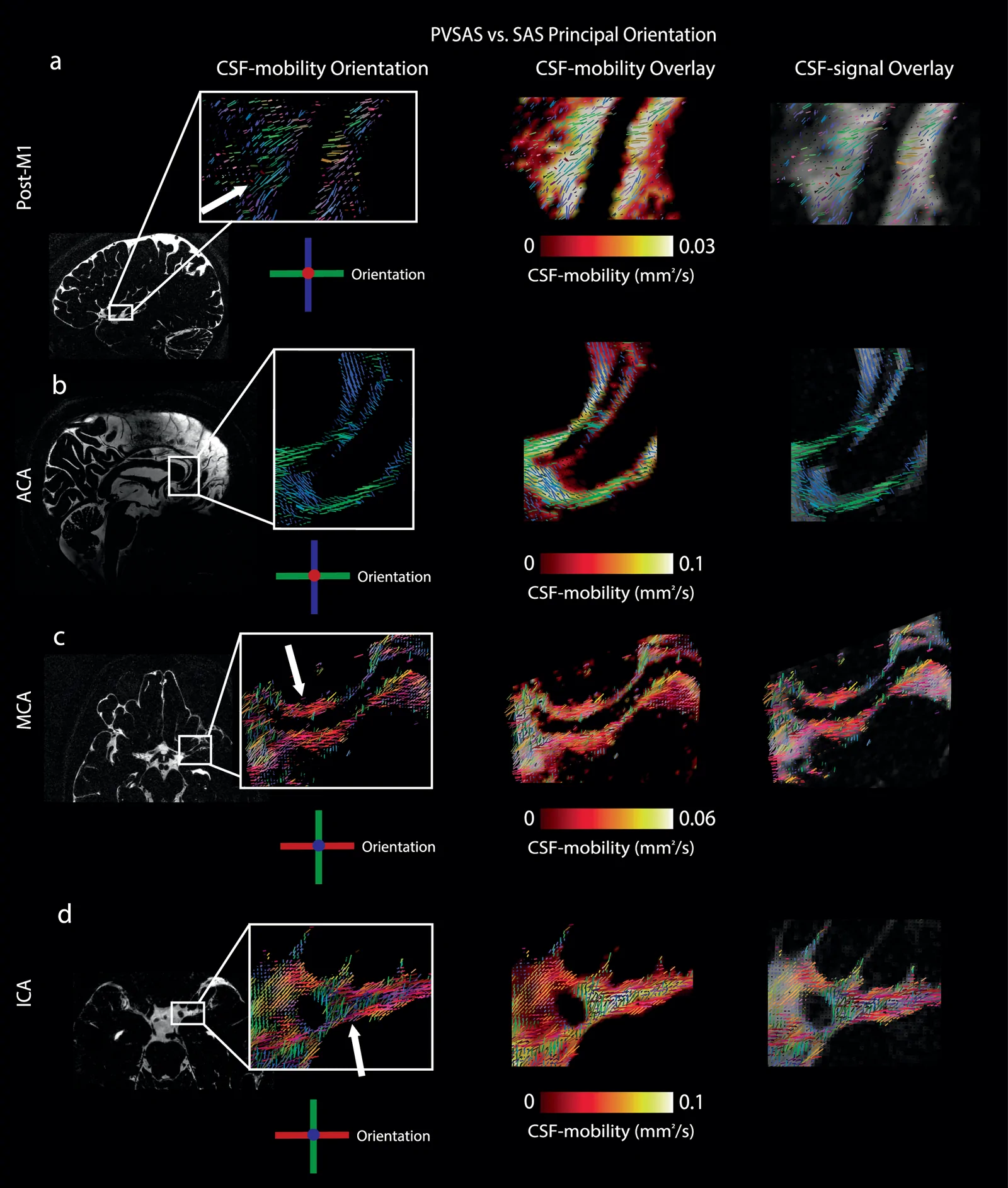
Principal orientations within the perivascular subarachnoid space (PVSAS). Principal orientations are overlaid on a through-plane of the PVSAS, shown on both the CSF-mobility and the non-motion-sensitized (gray) images. Line colors indicate orientation: red = left-right, green = anterior-posterior, blue = superior-inferior. White arrows point to PVSAS. **a** In the post-M1 PVSAS, CSF principal orientation shows greater directional coherence, indicated by the blue and green lines. In the subarachnoid space (SAS), CSF principal orientation shows lower directional coherence, reflected by more heterogeneous lines. **b** In the ACA PVSAS, CSF principal orientation travels anterior-posteriorly and then travels around the vessel superior-inferiorly, indicated by the green and blue lines; there is limited SAS present. **c** Around the M1, large red lines within the PVSAS indicate more directionality in the left/right direction. In the SAS near the M1, there is a more heterogeneous color distribution, meaning less directional coherence. **d** The ICA shows two compartments in the CSF-mobility, which is reflected in the principal orientation: in the high CSF-mobility region, there is more directional coherence, and lower directional coherence in the lower CSF-mobility region.

Principal orientation of the post-M1 CSF-mobility shows a more homogeneous directionality within the PVSAS compared to the SAS (Fig. 5a). The steep drop-off in CSF-mobility from the PVSAS to SAS and increased PVSAS directionality is reflected in the intrathecal contrast agent scans, where the contrast progresses fastest close to the vessel (Fig. 2d, h, l).

**Fig. 6:**
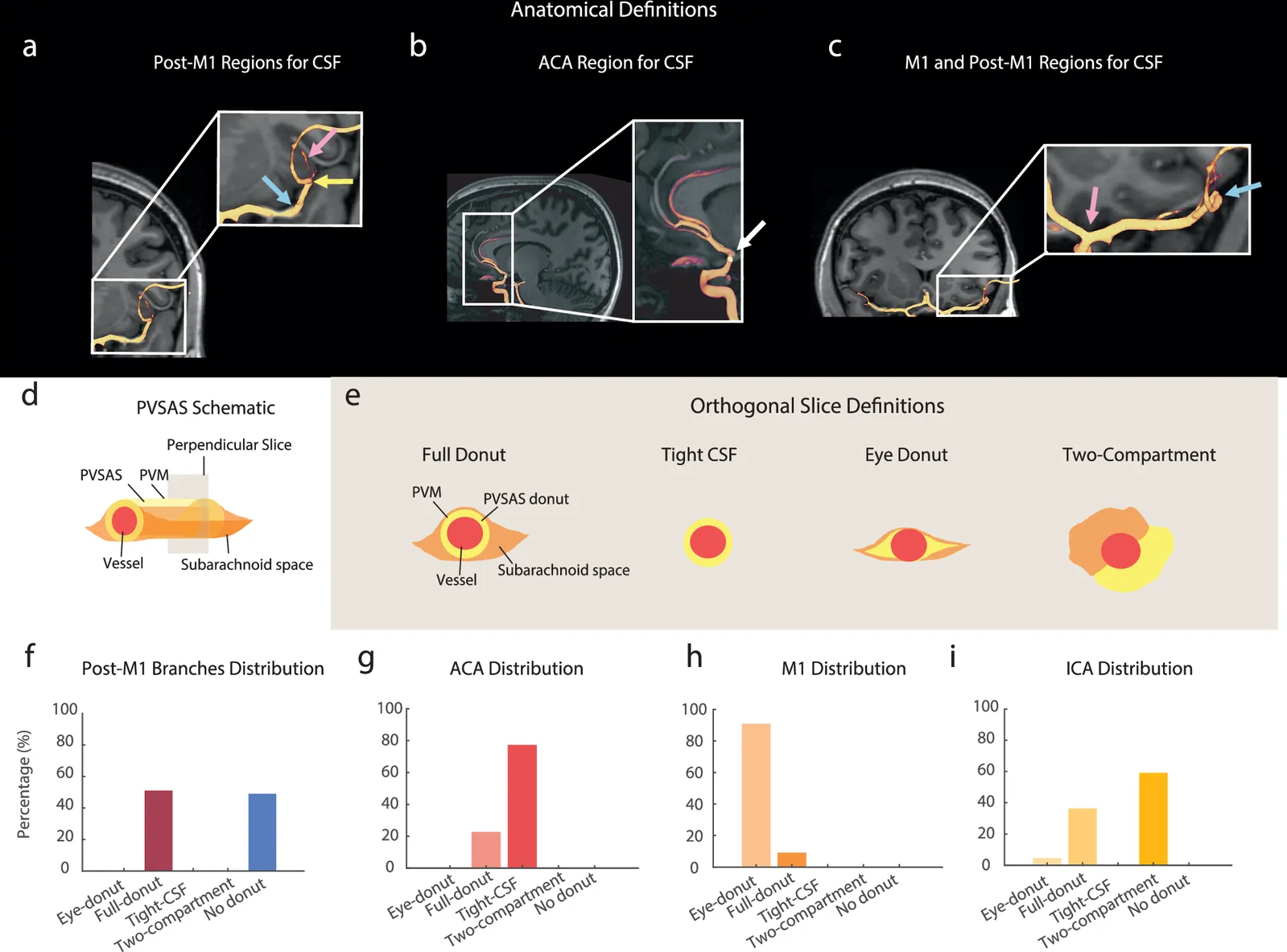
Characterizing perivascular subarachnoid space (PVSAS). **a** 3D representation of the arterial tree on top of a T1-weighted anatomical transverse slice shows an example of arteries that were rated in post-M1 distribution (pink arrow points to a smaller artery). The blue arrow points to turn from M1 to M2. The yellow arrow points to turn from M2 to M3. **b** A 3D representation of the anterior cerebral artery (ACA) on top of a T1-weighted anatomical sagittal image defines the ICA-to-ACA transition (white arrow). **c** 3D representation of middle cerebral artery (MCA) on top of T1-weighted anatomical coronal image defines arterial transition (internal carotid artery (ICA)-to-M1) (pink), and M1-to-M2 transition (blue). **d** Schematic illustration of perivascular subarachnoid space (PVSAS). **e** Cross-sectional schematics of CSF shapes. **f** The proportion of PVSAS located within the post-M1 arterial segments indicate 51% of visible arteries branching off the MCA have full-donuts (*n* = 93 segments, 11 subjects). **g**–**i** Distributions of observed CSF shapes around ACA, M1, and ICA arteries (*n* = 21 segments, 11 subjects). All source data for this figure are provided as a Source Data file.

When examining the CSF-mobility by visual inspection in healthy subjects, we consistently saw four distinct patterns related to PVSAS that we classified based on spatial distribution and CSF-mobility changes: full-donut, tight-CSF, eye-donut, and two-compartment (Fig. 6d, e), which are described in detail in the methods. Classification criteria were based on the extent of high CSF-mobility around the vessel, its presence within the SAS, and the magnitude of CSF-mobility changes. All arteries within the post-M1 segments, and their visible branches on non-motion-sensitized CSF-STREAM scans, were included in the post-M1 PVSAS classification.

The full-donut PVSAS were identified in ~51% of arteries in the post-M1 segment (Fig. 6f). These spatial patterns closely matched those seen in intrathecal contrast-enhanced scans from reference patients without relevant health findings, in which the contrast agent similarly accumulated along post-M1 closely around the arteries, within a larger not-enhancing (or later-enhancing) SAS. The remaining ~49% of arteries that do not exhibit PVSAS may reflect either the closeness of the PVSAS to the vessel wall, low CSF-mobility leading to an absence of CSF-mobility contrast with the surrounding SAS, or a complete absence of PVSAS. The PVSAS morphology varied across subjects: post-M1 vessels often showed asymmetric full-donut PVSAS, reflecting some structural heterogeneity that has also been observed via intrathecal contrast scans (Fig. 2e, i).

Beyond the post-M1 vessels, we observed the ACA running through a tightly bounded anatomical corridor with almost no surrounding CSF, and CSF-mobility in this region reflected that spatial constraint. Cross-sectionals show how the ACA CSF-mobility does not change within the restricted space, but stays high (Fig. 3a, d, g, Supplemental Figs. 3c, 9a–c). The principal orientation of CSF-mobility shows how CSF around the ACA moves along the vessel, i.e. first anterior-posteriorly, and then, as the ACA turns around the genu of the corpus callosum, superior-inferiorly (Fig. 5b). Around the ACA, there is often only a single tight-CSF compartment: the ACA showed in ~77% tight-CSF and ~23% full-donuts (Fig. 6g, *p* = 5.9 × 10^−6^, Wilcoxon rank-sum, Bonferroni-corrected, 3 tests). These patterns parallel findings from intrathecal contrast scans, which show concentrated PVSAS running closely along the ACA with little surrounding SAS (Fig. 3j, Supplemental Fig. 4c, g).

Intrathecal contrast agent-based detection of PVSAS is very time-sensitive: if the scan is performed too early, the contrast agent has not yet arrived; if scanned too late, it has already spread throughout the entire SAS^10^. While this approach is useful for tracking overall contrast distribution to the brain tissue, it will only provide snapshots of CSF dynamics. In contrast, our non-invasive strategy is tracer-independent and higher in resolution, which could allow for the detection of PVSAS further up or down the vascular tree beyond the post-M1. Because of this, we examined arteries supplying more proximal territories, including the M1 and the ICA.

In M1 PVSAS cross-sections, CSF-mobility displayed high CSF-mobility in a triangle on adjacent sides of the vessel, in an ‘eye’ shape (Fig. 3b, Supplemental Fig. 3b). Through-plane views again revealed high CSF-mobility extending along the length of the M1 segment, within a lower CSF-mobility area (Fig. 3e) and this was reflected in an increased CSF-mobility in the M1 PVSAS compared to SAS (Fig. 4b: M1 PVSAS mean = 0.04 ± 0.005 mm^2^/s, post-M1 SAS mean = 0.02 ± 0.005 mm^2^/s; 21 segments, 11 subjects, *p* = 6.5 × 10^−8^, paired *t*-test, Bonferroni-corrected, 2 tests). Once again, the non-motion-sensitized M1 CSF indicated no significant change between the SAS and the PVSAS (Fig. 4e, 21 segments, 11 subjects, M1 *p* = 0.32, paired *t*-test). A cross-sectional plot of the PVSAS and the SAS surrounding the M1 shows a steep drop-off from ~0.08 mm^2^/s in the PVSAS to ~0.02 mm^2^/s in the surrounding SAS (Fig. 3h). The principal orientation of the M1 PVSAS CSF-mobility shows that there is homogeneous directionality in the right-left direction, and the SAS has more heterogeneous directionality (Fig. 5c). The M1 PVSAS pattern parallels intrathecal contrast scans that show higher contrast close to the MCA in an ‘eye’ shape, with lower contrast in the SAS (Fig. 3k). Across subjects, ~91% of M1 arteries exhibited eye-donuts, while the remaining ~9% displayed full-donuts (Fig. 6h, *p* = 7.9 × 10^−6^, Wilcoxon rank-sum, Bonferroni-corrected, 3 tests).

To further characterize PVSAS space and understand where these perivascular compartments originate, we examined the ICA and its transition points to the MCA and the ACA. At the ICA level, CSF-mobility often appears as sharply bounded regions: a high CSF-mobility area is observed on one side and, on the other side, lower CSF-mobility (Fig. 3c, f, Supplemental Fig. 3d). In cross-sectional plots, there is a steep change depending on the side of the vessel - often dropping from ~0.1 mm^2^/s to ~0.02 mm^2^/s (Fig. 3i). The ICA principal orientation indicates more directionality in the high CSF-mobility compartment compared to the low CSF-mobility area (Fig. 5d). In ~59% of ICA sections, the CSF configuration resembled two-compartments; ~5% showed eye-donuts, and ~37% exhibited full-donuts (Fig. 6i). There were significantly more two-compartments compared to full-donuts at the ICA level (Fig. 6i, *p* = 9.5 × 10^−6^, Wilcoxon rank-sum, Bonferroni-corrected, 3 tests). As the ICA bifurcates into the A1 and M1, we observed a shift from open regions into more spatially constrained, PVSAS-shaped regions. As with previous CSF segments, non-motion-sensitized scans confirmed that reductions in CSF-mobility were not due to reduced CSF presence, but rather to altered fluid dynamics (Fig. 3c, f, i). Notably, at the ICA-to-ACA transition, we frequently observed regions of high CSF-mobility surrounded by areas of lower mobility that would then become tight CSF-mobility along the ACA. In contrast, anterior-inferior CSF-filled basilar cisterns such as the interpeduncular and pontine cisterns showed no evidence of PVSAS and, instead, a large pool of homogeneous CSF-mobility.

When comparing the PVSAS across vessels, there is higher CSF-mobility around the M1 and ACA (M1 mean = 0.04 ± 0.005 mm^2^/s, ACA mean = 0.04 ± 0.006 mm^2^/s) compared to the post-M1 PVSAS (post-M1 mean = 0.03 ± 0.008 mm^2^/s) (Fig. 4c: M1 vs post-M1 *p* = 1.49 × 10^−3^, ACA vs. post-M1 *p* = 1.12 × 10^−3^, paired *t*-test, Bonferroni corrected, 3 tests).

### High CSF-mobility PVSAS near the superior sagittal sinus

**Fig. 7:**
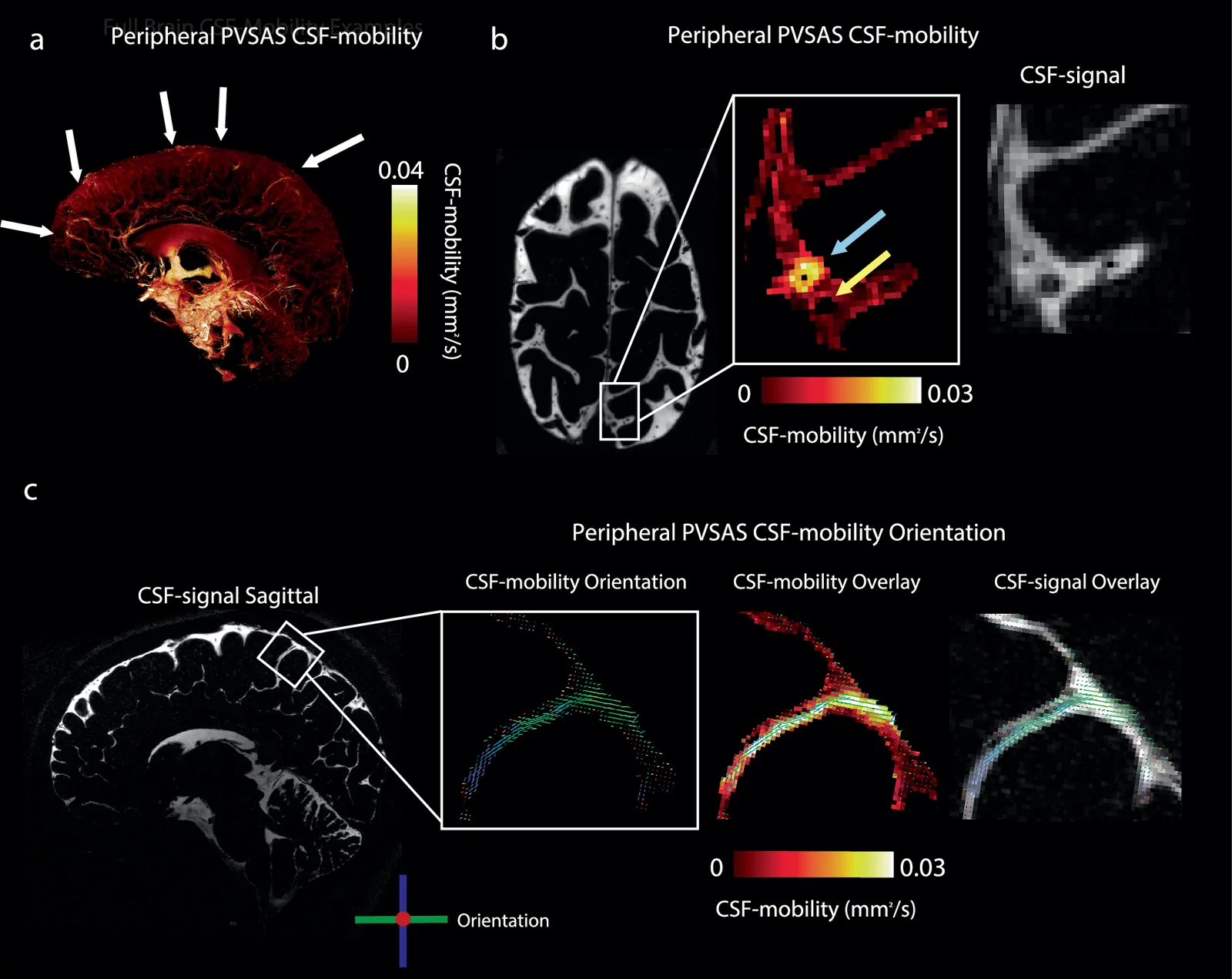
Perivascular subarachnoid space (PVSAS) near the superior sagittal sinus (SSS) was observed in two out of eleven subjects. **a** Whole-brain example from a subject exhibiting numerous high CSF-mobility PVSAS near the SSS (white arrows). **b** Peripheral high CSF-mobility PVSAS near the SSS, and the non-motion-sensitized scan (gray) confirms the presence of CSF, indicating that reduced mobility is not due to reduced CSF signal. Two adjacent vessels show that not all peripheral PVSAS have high CSF-mobility (blue arrow indicates vessel with high CSF-mobility; yellow indicates vessel with no visible high CSF-mobility PVSAS). **c** Principal orientation of CSF-mobility around peripheral PVSAS shows there is more directional coherence within the PVSAS , indicated by the green and blue lines. In the subarachnoid space (SAS), there is lower directional coherence. Lines are overlaid on the CSF-mobility and the non-motion-sensitized (gray) images. Line colors indicate orientation: red = left-right, green = anterior-posterior, blue = superior-inferior.

Given that ACE points have recently been described as openings around bridging veins where molecules and fluid are thought to move between the SAS and the dura^7^, we examined CSF-mobility near the SSS. In 2 out of 11 participants, we observed regions of high-mobility CSF PVSAS around vessels near the SSS, suggesting the existence of PVSAS beyond the major cerebral arteries (Fig. 7a, b). The principal orientation of the CSF-mobility in these PVSAS moves along the vasculature and is more homogeneous within the PVSAS compared to the SAS (Fig. 7c).

These high CSF-mobility peripheral regions appeared in two distinct patterns. They either extend continuously from a major artery, such as the ACA, and run to the periphery near the SSS, or appear de novo near the SSS, without an evident high CSF-mobility arterial connection. These peripheral PVSAS were heterogeneous and were found around veins, arteries, and often near arachnoid granulations.

### Cardiac and respiration dynamics modulate the PVSAS

**Fig. 8:**
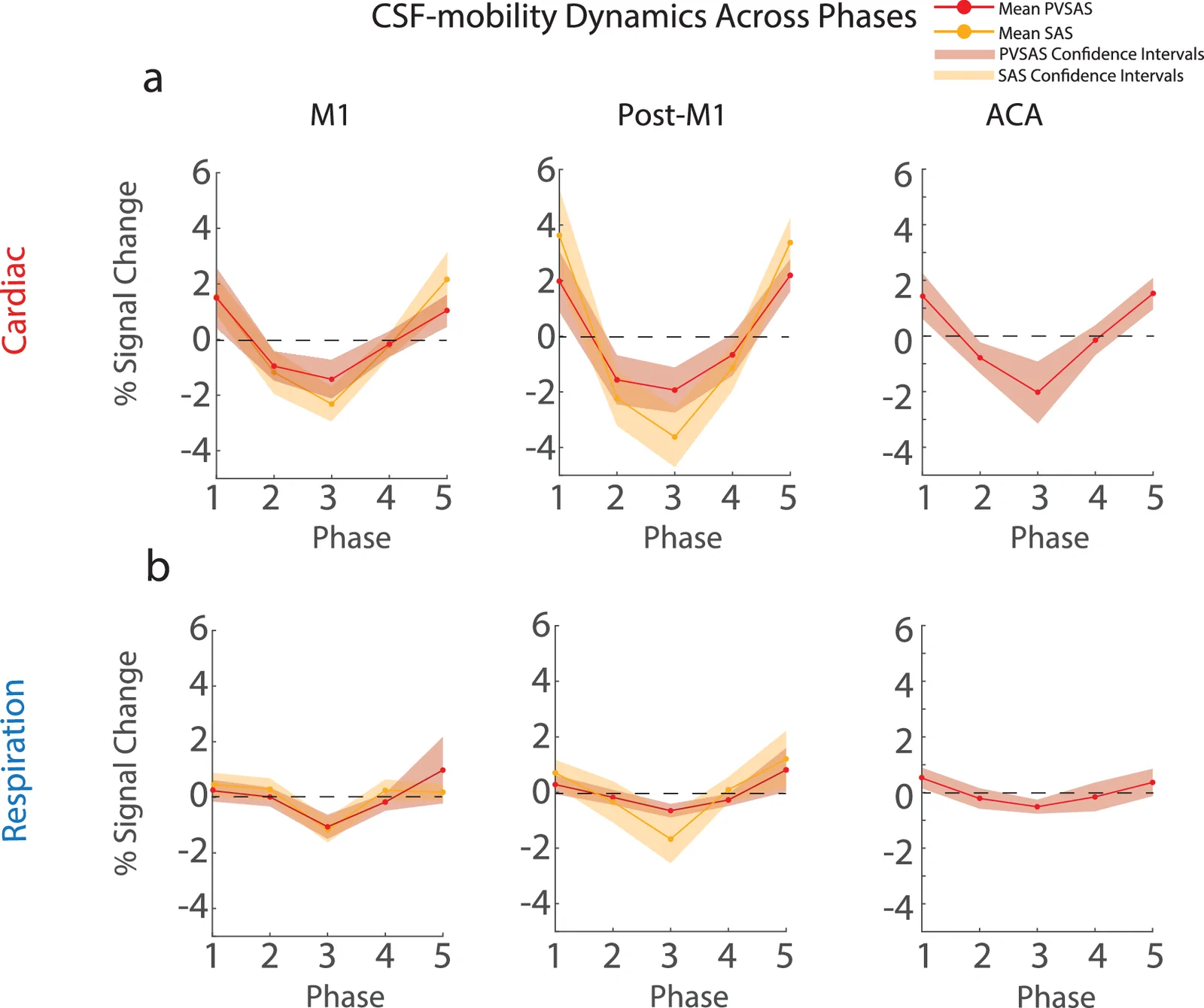
Cardiac cycle and respiration modulate CSF-mobility across phases in the perivascular subarachnoid space (PVSAS) and the subarachnoid space (SAS). CSF-mobility signal change from the mean value over phases (in %). Each line shows the mean across individuals, and the shaded error areas represent confidence intervals of SD × 1.96/√n (*n* = 11). Source data are provided as a Source Data file. **a** Cardiac dynamics modulate PVSAS signal across CSF-mobility phases in M1, post-M1, and the anterior cerebral artery (ACA). However, the relative percent signal change does not differ significantly between PVSAS and SAS in the M1 and post-M1. **b** Respiratory dynamics produce smaller modulations across phases in M1, post-M1, and ACA, with no significant differences in relative percent signal change between PVSAS and SAS in the M1 and post-M1.

CSF is thought to be driven by cardiac, respiration, and vasomotor activity^1,14–18^. Because of this, we examined whether the PVSAS would have distinct cardiac and respiration dynamics in comparison with the SAS. We found that overall, CSF-mobility in PVSAS and SAS is modulated most with the cardiac cycle (Fig. 8a) and is minorly modulated with respiration (Fig. 8b) in the M1, post-M1, and ACA PVSAS. In the PVSAS, the absolute cardiac and respiratory CSF-mobility pulsatility is higher than in the SAS. However, the relative mobility fluctuations measured as the change in mobility from the mean within the SAS and the PVSAS are comparable.

## Discussion

The current view is that the PVSAS is bound by a semipermeable perivascular membrane. In intrathecal contrast studies, the contrast agent initially accumulates near the vessel, then diffuses more broadly into the SAS over time. Our CSF-STREAM data aligns with this view: CSF-mobility and its principal orientation are higher in the CSF around the MCA and ACA than in the surrounding SAS, consistent with PVSAS existence.

Additionally, the CSF-mobility PVSAS to SAS transition shows a sharp drop-off (e.g., Fig. 2a–c, e–g, i–k, Supplemental Figs. 3a, 4b, f, 9g–i), which may indicate the presence of a membrane. This is corroborated quantitatively by steep drop-off profiles transitioning from the PVSAS to the SAS (Figs. 2c, g, k, 4a, b) and by the concentric circle analysis of post-M1 vessels, which show drop-off at ~1.5 mm from the vessel wall (Supplemental Figs. 7, 8). With the concentric circles, we also found that the majority of the post-M1 PVSAS examined showed a ≥ 20% drop-off from the maximum mobility. If elevated CSF-mobility near vessels arises solely from cardiac and respiratory pulsations, we would instead predict a gradual, rather than abrupt, decline with distance. Consistent with a membrane, intrathecal contrast scans^10^ (Fig. 2d, h, l, Supplemental Figs. 2c, d, g, h, 4a, c, e, g) show that the contrast stays close to the vessel for a prolonged time (e.g., MCA) and then diffuses. The increase in CSF-mobility within the PVSAS likely also explains the faster dispersion of intrathecal contrast agent within the PVSAS. However, the presence of a membrane should still be considered a hypothesis until confirmed anatomically.

CSF-mobility is specifically measured by isolating the CSF signal from blood and tissue using T_2_-preparation and a long echo-time (495 ms)^14^, thereby limiting partial volume effects. To ensure that the signal in the PVSAS was not driven by partial volume effects, we measured the signal intensity in the PVSAS and SAS in non-motion-sensitized scans (Fig. 4d, e, Supplemental Fig. 7g, h). If the CSF-mobility changes observed in SAS vs PVSAS were due to partial volume artifacts or pulsation-induced noise, signal intensity differences between the SAS and PVSAS would be expected. Importantly, we found no change in signal intensity in the SAS vs PVSAS, demonstrating that the observed changes in CSF-mobility were not due to differences in CSF presence.

We also parsed the characteristics of the PVSAS around major arteries: post-M1 segments primarily exhibited full-donut PVSAS, M1 segments mostly exhibited eye-donut PVSAS, and the ACA predominantly showed tight-CSF. These changes in PVSAS likely reflect the anatomical relationships between the PVSAS and adjacent structures, which shape the overall CSF geometry.

At the ICA bifurcation, CSF-mobility shifts from a larger pool to constrained PVSAS regions, suggesting that the PVSAS begins at the ICA-to-MCA and ICA-to-ACA transitions. Additionally, the CSF-mobility PVSAS speed decreases through the vascular tree: higher CSF-mobility was found in the M1 PVSAS and ACA PVSAS in comparison with the post-M1 PVSAS.

Beyond the major vascular tree, the peripheral high-mobility PVSAS were found heterogeneously around both arteries and veins and often near arachnoid granulations, albeit only seen in a minority of subjects (Fig. 7a–c). These peripheral high-mobility PVSAS often run from around a major artery to the periphery, or they appear de novo near the SSS. Interestingly, the example shown in Fig. 7b shows the presence of several vessels close to each other (see CSF-signal insert in Fig. 7b), but higher CSF-mobility is only observed around one of the vessels, pointing towards a physiological explanation rather than methodological. The observations of peripheral PVSAS were sparse, but do not appear to reflect methodological artifacts. Signal changes in the non-motion-sensitized scan, head motion, and partial volume effects from adjacent vessels were each considered to exclude the possibility of an artifactual observation. Though they cannot be ruled out entirely, these effects would not be expected to act selectively on the signal at the brain surface or around individual vessels. We applied conservative criteria for rating peripheral PVSAS (see “Methods”). Importantly, image quality in both subjects was high, with minimal noise or artifacts. We therefore think these peripheral PVSAS may reflect true physiological CSF dynamics and could correspond to a recently described leptomeningeal perivascular network involved in fluid shunting, CSF drainage, and immune surveillance^19^.

Finally, CSF-mobility is similarly modulated in PVSAS and SAS by cardiac and respiration dynamics, with no relative difference between the two areas. The cardiac cycle was found to have more influence than the respiratory cycle, in line with previous work^14^. As the CSF-mobility is higher in PVSAS than in SAS, the equal relative fluctuations still translate into larger excursions. The origin of the higher CSF-mobility might therefore be more easily explained by the presence of a membrane.

CSF-STREAM’s low b-value motion sensitizing strategy primarily encodes for CSF flow (whether bulk, parabolic, oscillatory or random) rather than diffusion^20,21^. This is also reflected in the CSF-mobility values measured in SAS/PVSAS (~10–50 × 10^−3^ mm^2^ s^−1^), which are more than ten times higher than brain tissue water diffusion (~1 × 10^−3^ mm^2^ s^−1^)^22^. However, CSF-STREAM does not measure phase accrual and therefore cannot determine flow directionality (i.e., it cannot differentiate right-to-left from left-to-right flow, but it can show increased mobility in the right-left direction versus, e.g., feet-head direction)^14^. Previous intrathecal scans showing contrast traveling up along the PVSAS, with rapid arrival in the ACA and MCA (~23 min to A1; ~38 min to M1), also hint at possible net flow^10^. Additionally, cardiac and respiratory cycles modulate CSF-mobility in both the SAS and PVSAS. If the measured CSF-mobility were purely diffusion-derived, cardiac and respiratory dynamics would be expected to have no effect^23–25^.

In terms of limitations, beyond the lack of ability to detect net flow, we have a small cohort. However, this cohort size has been used previously to assess CSF dynamics^12,14^. Furthermore, the intrathecal images and non-invasive CSF-STREAM data were obtained from separate cohorts. Though a limitation, the consistency of PVSAS across both modalities supports the robustness of these findings. Although we implemented a quantitative pipeline, future work should automate the characterization of the PVSAS around the vasculature across the brain. Moreover, we have a long scan time that could lead to motion artifacts, but we were careful to exclude any participants with too much motion^14^. Importantly, although the overall scan time is ~38.5 min, each sub-scan lasts ~5.5 min, making standard motion correction appropriate. Ongoing work aims at reducing the scan time to ~20 min through further acceleration^26^, and this shorter protocol will be implemented in future clinical studies^27–29^. Additionally, we have low temporal resolution when parsing the cardiac and respiration dynamics, but it has been shown previously that this temporal resolution is sufficient to detect physiological modulation above noise levels^14^. Finally, we were unable to directly compare CSF-mobility with intrathecal imaging for the ICA, because intrathecal contrast reaches it too rapidly and fills the compartment globally^10^, precluding spatial assessment of PVSAS with contrast at the ICA level.

Alternative barriers, such as the debated subarachnoid lymphatic-like membrane (SLYM)^30,31^, outer pial layers^32,33^, or leptomeningeal sheath cells^34^, may constitute, overlap with, or act independently of the PVSAS in shaping CSF compartmentalization. The presence of distinct, high-mobility PVSAS in the absence of pathology raises important questions: how do these compartments circulate CSF, and do they exhibit unidirectional CSF movement? Future work should investigate how PVSAS vary with vasomotion fluctuations, how they change in aging and neurodegeneration, and their potential clinical relevance for drug delivery.

## Methods

### Subjects

Twelve healthy individuals (mean age: 34 ± 13 years; 9 females, 3 males) participated in the study. Data from one participant was excluded due to motion artifacts. All participants were screened for MRI contraindications and provided written informed consent. The study was conducted in compliance with the Leiden University Medical Center Institutional Review Board (Leiden, The Netherlands) under authorization number P07.096. No sex or gender analyses were done because of the small sample size. Each subject was compensated with a €20 voucher for every completed scan.

### MRI scan acquisition

All scans were performed on a 7 Tesla MRI scanner (Achieva, Philips, Best, The Netherlands) with a quadrature birdcage head coil and a 32-channel receive coil array (Nova Medical, Wilmington, MA, USA). This retrospective study utilized data that had been previously published. Detailed methods for image acquisition, reconstruction, and CSF-mobility quantification are available in earlier work^14^. In short, CSF-STREAM consists of seven sub-scans: one without motion encoding and six with motion-sensitizing gradients (3.5 mm/s) applied in different orthogonal directions. Importantly, the CSF signal is isolated while suppressing blood and brain tissue contributions thanks to a T2-preparation followed by a long echo-time turbo-spin-echo readout (0.45 mm isotropic resolution, FOV = 250 × 250 × 190 mm, TE = 495 ms, TR = 3.4 s, TSE-factor = 146).

### Post-processing

#### CSF-mobility, principal orientation, and Fractional Anisotropy

T1-weighted and CSF-STREAM images were registered using Elastix version 4.9.0, and manually corrected with ITK-SNAP 4.2.2. if needed. From these CSF-STREAM measurements, CSF-mobility, the principal orientation, and fractional anisotropy (FA) maps were calculated in MATLAB as the mean eigenvalue of a rank-two positive-definite tensor, similar to diffusion tensor imaging (DTI). CSF-mobility is more sensitive to flow than to diffusion because it is acquired at a very low b-value (motion encoding gradients of 3.5 mm/s equivalent to a b-value of 13 s^2^/mm). The CSF-mobility maps quantify average CSF motion over the acquisition period (38 min and 30 s).

#### Vasculature definition

The vasculature was defined by examining the registered T1-weighted image. The MCA, ACA, and ICA were all defined based on their T1-weighted inflow-dependent bright appearance. Sections of the MCA and ACA were identified by the sharp turns in the vasculature^12^ (Fig. 6a–c). Additionally, the non-motion-sensitized scan image was overlaid onto the T1-weighted scan to confirm the absence of CSF in the region corresponding to the vessel.

Vessels surrounded by high CSF-mobility near the SSS were classified as artery, vein, or adjacent to an arachnoid granulation by two experienced neuroradiologists, using both T1-weighted and CSF-STREAM non-motion-sensitized images.

#### PVSAS characterization

To categorize CSF-mobility patterns around the vessel, we defined PVSAS classifications based on spatial distribution and CSF-mobility changes: full-donut, tight-CSF, eye-donut, two-compartment, and no-donut. To minimize subjective bias, we established a predefined set of criteria for classification based on an initial reference subject, and these criteria were then applied consistently to all subsequent cases. Specifically, CSF-mobility patterns were categorized according to predefined morphology with quantitative thresholds.

The categories were defined as follows:

- **Full-donut:** high CSF-mobility around ≥50% of the vessel circumference.
- **Eye-donut:** high CSF-mobility visible on both lateral sides of the vessel within a lower CSF-mobility area.
- **Tight-CSF:** high CSF-mobility visible around at least 50% of the vessel with less than a two-voxel circumference of low CSF-mobility. Additionally, to be classified as tight-CSF, the mobility had to be higher than 0.02 mm²/s. The lower bound was chosen based on prior reports indicating this value marks the approximate boundary between higher-mobility around the MCA and lower-mobility CSF in the perivascular spaces, and SAS near the sulci^14^
- **Two-Compartment:** high-CSF-mobility with adjacent low CSF-mobility next to the vessel.
- **No donut:** no marked CSF-mobility around the vessel relative to the surrounding SAS, or less than 50% of the vessel circumference.
  Quantitative thresholds were applied to further reduce rater bias:
- **High CSF-mobility:** a circumference of ≥2 voxels and local ≥20% change relative to SAS.

These definitions were consistently applied to all datasets, and classification was based on the thresholds rather than subjective judgment.

CSF-mobility maps were masked to remove noise by applying a subject-specific threshold on the non-motion-sensitized scan (either 100 or 150 a.u.).

For the post-M1 characterization, all arteries (93 segments, 11 subjects) within the post-M1 (M2-M4) segments, as well as branching arteries originating from these segments that were visible with CSF-STREAM, were included if distinguishable from other arteries. Each branch was defined by the presence of a sharp turn. Each CSF segment around each artery was then categorized into full-donut, tight-CSF, eye-donut, two-compartment, and no-donut. The CSF-mobility maps were overlaid onto the non-motion-sensitized scan image to validate the presence of CSF.

#### PVSAS ‘donuts’ and CSF two-compartment visualizations

Visualizations of MCA, ACA and ICA were created in ParaView (5.13.2) with the ‘black-body radiation’ color scale. Reformatting of images in ParaView was done for the orthogonal and through-plane examples, which introduced interpolation. CSF-mobility and FA maps were masked to remove noise by applying a subject-specific threshold on the non-motion-sensitized scan (either 100, 150, or 175 a.u.). All subjects are represented in the visualizations between the main text and the supplemental.

#### Cross-sectional

Cross-sectionals were manually delineated in ITK-SNAP 4.2.2. on CSF-mobility maps to show the width of the CSF-mobility change and the sharp drop in CSF-mobility, and the non-motion-sensitized scan is also plotted. All plots were made using MATLAB 2020b (The MathWorks, USA). In the corner of each cross-sectional plot, the ROI on the CSF-mobility map in ITK-SNAP is shown in the ‘Hot’ color scale.

#### Bar plots

Bar plots were generated in MATLAB using the bar function to show the percentage distribution of visible PVSAS types across arteries.

#### Post-M1 and M1 SAS vs. PVSAS CSF-mobility Comparison

ROIs were manually drawn on an M1 and post-M1 slice for each subject using CSF-mobility maps thresholded based on the non-motion-sensitized scan to remove background noise (either 100, 150, or 175 a.u.). The PVSAS ROI encompassed an area approximately two voxels from the artery’s circumference. The SAS ROI was placed further from the artery cross-section, avoiding regions with vessel turns, and remained within the same imaging plane. The SAS ROI aimed to follow the full circumference of the artery, but some variability occurred due to anatomical heterogeneity in SAS shape (Supplemental Fig. 1a–f). One subject did not show clear PVSAS in one hemisphere, and therefore, the measurement was omitted.

#### Violin plots

Violin plots were created using the MATLAB Violinplot-Matlab-master function. The mean of post-M1 and M1 SAS vs. PVSAS CSF-mobility ROIs were plotted. Each subject relates to a corresponding line. Line and dots are jittered for visualization.

#### Statistical comparison

Paired two-tailed *t*-tests with Bonferroni correction were done to compare the mean CSF-mobility on post-M1, and M1 SAS and PVSAS ROIs as described above, and the same was done to compare the CSF-mobility across arteries (M1, post-M1, and ACA). Statistical analysis was implemented in MATLAB 2020b (The MathWorks, USA).

To compare the distributions of CSF-mobility shapes around different vessels, we performed Kruskal-Wallis tests to assess whether there were significant differences across multiple shape categories for the ACA, MCA, and ICA. This non-parametric test was chosen due to the ordinal nature of the shape ratings and the potential for non-normal distributions. If the Kruskal-Wallis test indicated a significant difference (*p* ≤ 0.05), we conducted post-hoc pairwise Wilcoxon rank-sum tests with Bonferroni correction to identify specific group differences.

#### Cardiac and respiration binning

CSF-STREAM k-space was retrospectively binned into six cardiac and six respiration phases to assess the CSF-mobility and FA dynamics over driving forces, as described before^14^.

Changes in CSF-mobility and FA were obtained across driving forces after normalizing the maps voxel-wise to the mean value over phases. To avoid phase cancellation within an ROI, the maximum of each timeseries was realigned voxel-wise to the first phase; this reference phase was excluded from visualization. Cardiac and respiration signals have previously been compared with random noise as a negative control^14^. The PVSAS and SAS ROIs were used to extract the signal over phases for the M1 and post-M1 areas. For the ACA,only the PVSAS was extracted because of the lack of surrounding SAS. Plots show CSF-mobility and FA change from the mean value over phases (in %). Each line shows the mean across individuals, and the shaded error areas represent confidence intervals of SD × 1.96/√n (*n* = 11).

#### Semi-automatic PVSAS analysis (concentric circles)

Vessel masks were first created by thresholding the registered T1-weighted anatomical image, in which we can take advantage of the inflow effect, as it makes the major arteries (MCA, ACA) have a bright signal. A seed point was placed at the internal carotid artery (ICA) and allowed to grow across the thresholded T1 volume, yielding an initial vessel mask. Any voxels within the initial vessel mask that overlapped with the CSF signal, due to registration imperfections in the non-motion-sensitized image, were manually removed from the mask. This mask was combined with the thresholded non-motion-sensitized CSF signal image, and morphological operations (imfill, imclose; MATLAB2024a) were applied to close residual gaps. The vessel mask was then dilated, and made to stop at the boundary of the combined mask, and the thresholded non-motion-sensitized CSF signal was subtracted to isolate the vasculature area (Supplemental Fig. 7a, b). Post-M1 segments of the MCA were manually delineated and extracted, and all vessel masks were visually inspected to exclude voxels falling outside the vessel boundary. Concentric rings were generated by iteratively dilating the post-M1 vessel masks (imdilate; MATLAB). This mask was applied to the non-motion-sensitized CSF-signal images and to the CSF-mobility maps (Supplemental Fig. 7c, d). CSF-mobility and CSF-signal values were extracted within each ring and plotted as a function of distance from the vessel. CSF-signal and mobility values were normalized subject-wise by the max peak. The M1 PVSAS was not included because of its ‘eye-donut’ shape.

### Intrathecal injections examples

#### Patients

Examples of intrathecal T1-weighted images with contrast agent were obtained at the Intervention Center at Oslo University Hospital, Oslo, Norway, in ten patients (mean age: 37 ± 16 years; 8 females, 2 males) who were referred for a possible CSF circulation disorder. The study was approved by the Institutional Review Board (2015/1868), Regional Ethics Committee (2015/96) and the National Medicines Agency (15/04932-7), and registered in the Oslo University Hospital Research Registry (ePhorte 2015/1868). All participants gave informed and written consent, and they received no compensation.

#### MRI protocol

All scans were acquired on a 3 Tesla MRI scanner (Ingenia, Philips Medical Systems) using a 32-channel head coil. Sagittal 3D T1-weighted volumes were acquired using the following parameters: TR/TE = shortest available (typically 5.1/2.3 ms), flip angle = 8°, FOV = 256 × 256 mm, acquisition matrix = 256 × 256 (reconstructed to 512 × 512). A total of 184 overlapping 1-mm slices were acquired and automatically reconstructed to 368 slices at 0.5-mm thickness. Scans were acquired continuously during the first hour following intrathecal injection, then repeated two hours post-injection. Intrathecal contrast (0.5 ml gadobutrol, 1 mmol ml^−1^; Gadovist, Bayer AB, Sweden) was administered at the lower lumbar level by an experienced interventional neuroradiologist. Detailed descriptions of methods have previously been reported^10^.

#### Intrathecal visualizations

PVSAS visualizations were created in ParaView (6.1.0) with the ‘gray scale’ color scale. Reformatting of images in ParaView was done for the orthogonal and through-plane examples, which introduced some interpolation. Intrathecal examples in Figs. 1d–f, 2d, h, l, 3j, k, and Supplemental Figs. 2c, d, g, h, 4a, c, e, g have previously been reported in a prior publication^10^.

### Reporting summary

Further information on research design is available in the Nature Portfolio Reporting Summary linked to this article.

## Supplementary information

Supplementary Information

Reporting Summary

Transparent Peer Review file

## Source data

Source Data

## Data Availability

Source data are available via Zenodo (https://doi.org/10.5281/zenodo.20814851)^35^. Source data are provided with this paper.

## Code availability

Code can be found at: https://github.com/ninafultz/csf_donuts, and archived via Zenodo (https://doi.org/10.5281/zenodo.20814851)^35^.
