# Supplementary material for: Non-invasive characterization of perivascular subarachnoid spaces
Source: Nat Commun. 2026 Aug 5;17:9496. doi: 10.1038/s41467-026-76306-9 (PMC13542271; doi:10.1038/s41467-026-76306-9)
Supplement: Supplementary file 1 [file 41467_2026_76306_MOESM1_ESM.pdf]

Non-invasive Characterization of Perivascular Subarachnoid Spaces

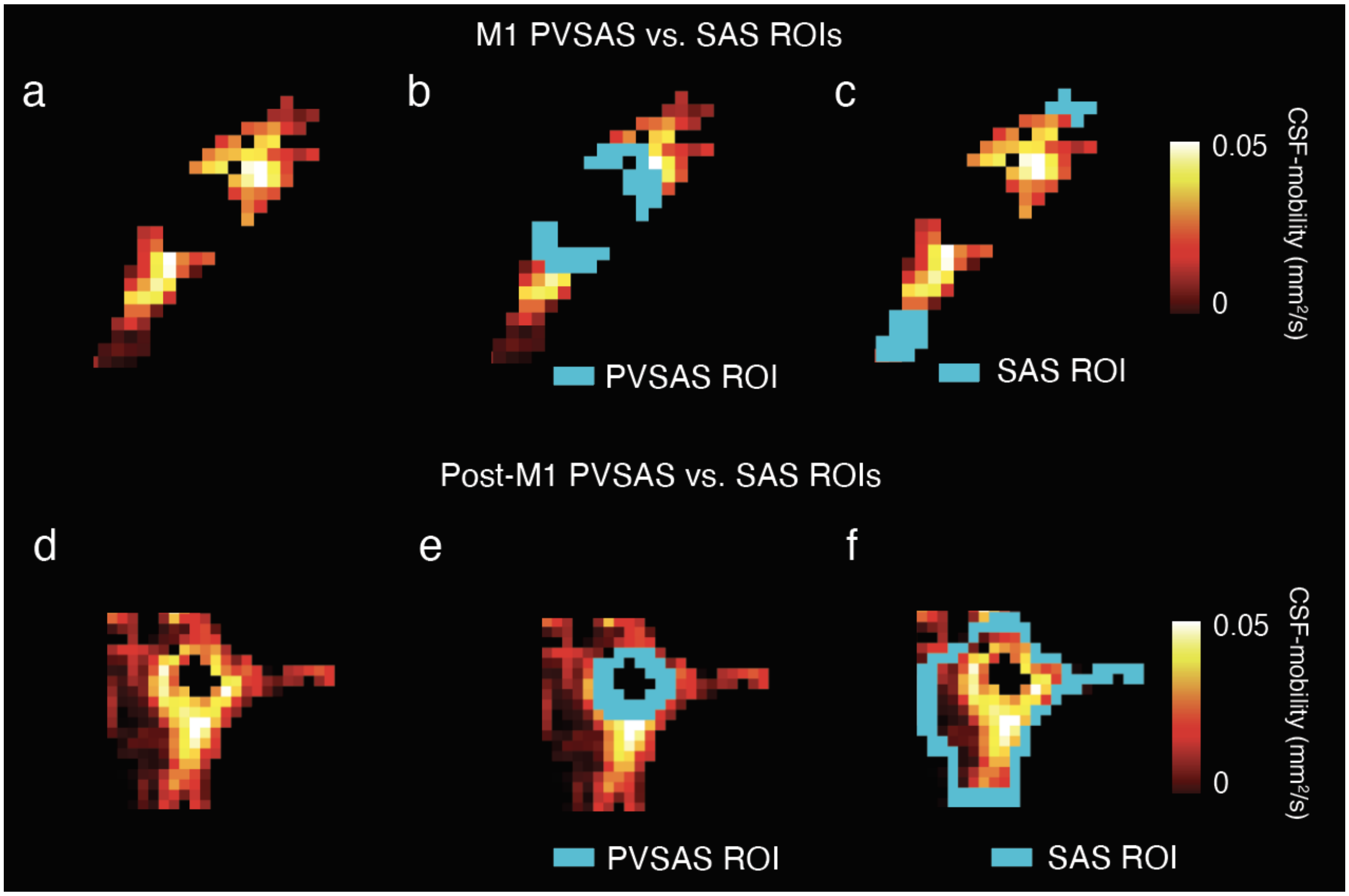

Supplemental Figure 1 | Examples of M1 and post-M1 perivascular subarachnoid space (PVSAS) and subarachnoid space (SAS) ROIs.

a) Representative slice showing CSF-mobility in the M1 region.

b/c) Example ROIs from the M1 region with subsection of the perivascular space (PVSAS) (b) and SAS (c).

d) Representative slice from the post-M1 region.

e/f) Corresponding example ROIs from the post-M1 region illustrating subsets of the PVSAS (e) and SAS (f).

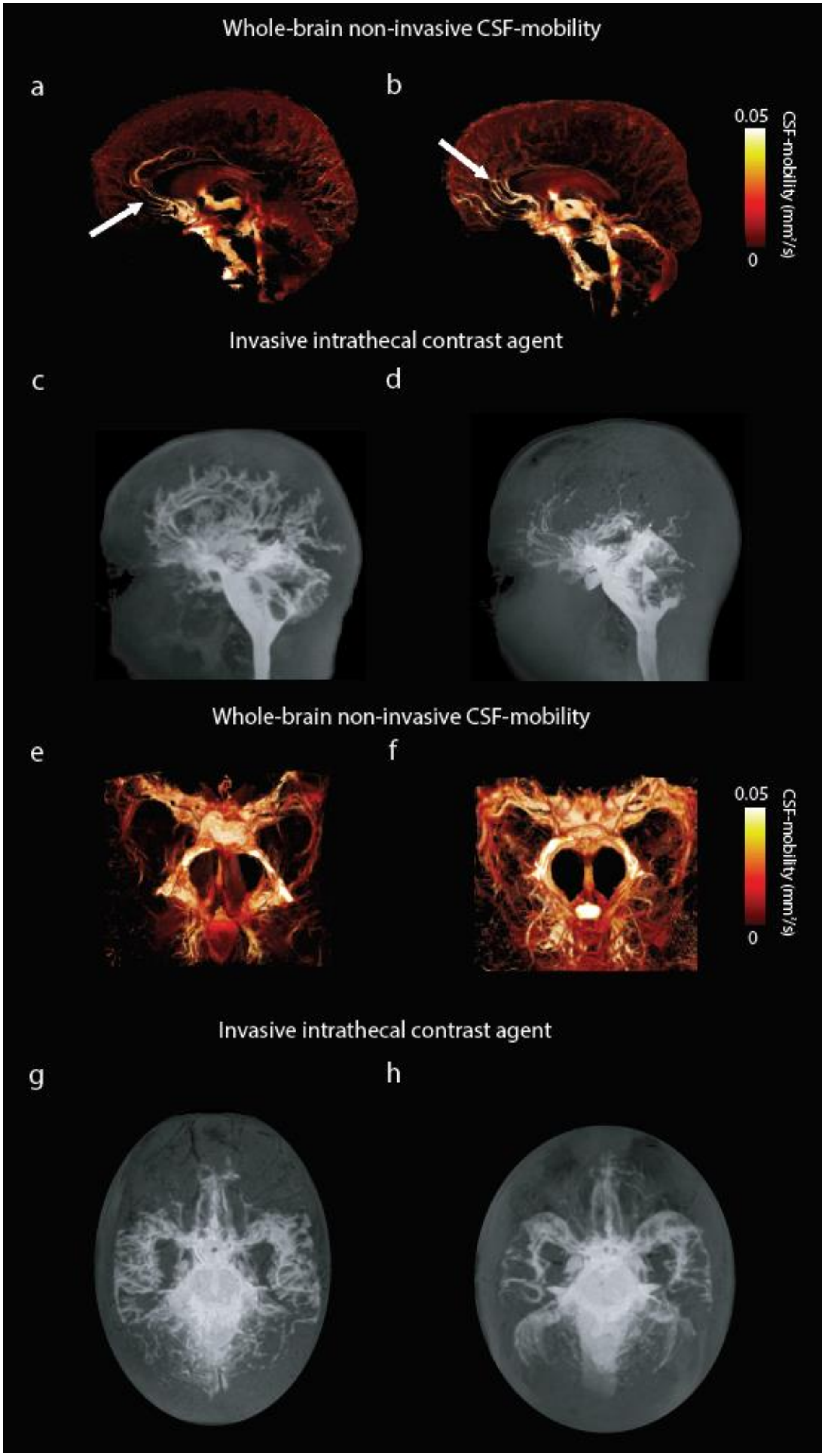
Whole-brain non-invasive CSF-mobility
a
b
0.05
CSF-mobility (mm²/s)
0
Invasive intrathecal contrast agent
c
d
Whole-brain non-invasive CSF-mobility
e
f
0.05
CSF-mobility (mm²/s)
0
Invasive intrathecal contrast agent
g
h

Supplemental Figure 2 | Examples of whole-brain non-invasive CSF-mobility and intrathecal contrast scans.
a/b) Representative sagittal examples of CSF-mobility scans in healthy controls shows perivascular subarachnoid space (PVSAS) surrounding the anterior cerebral artery (ACA) (indicated by white arrows).

c/d) Sagittal examples of intrathecal contrast scans of reference patients shows contrast in the PVSAS surrounding the ACA.

e/f) Transverse views of a CSF-mobility scans indicates high CSF-mobility PVSAS around the middle cerebral artery (MCA).

g/h) Transverse views of a intrathecal contrast scans of reference patients show contrast filling the PVSAS around the MCA.

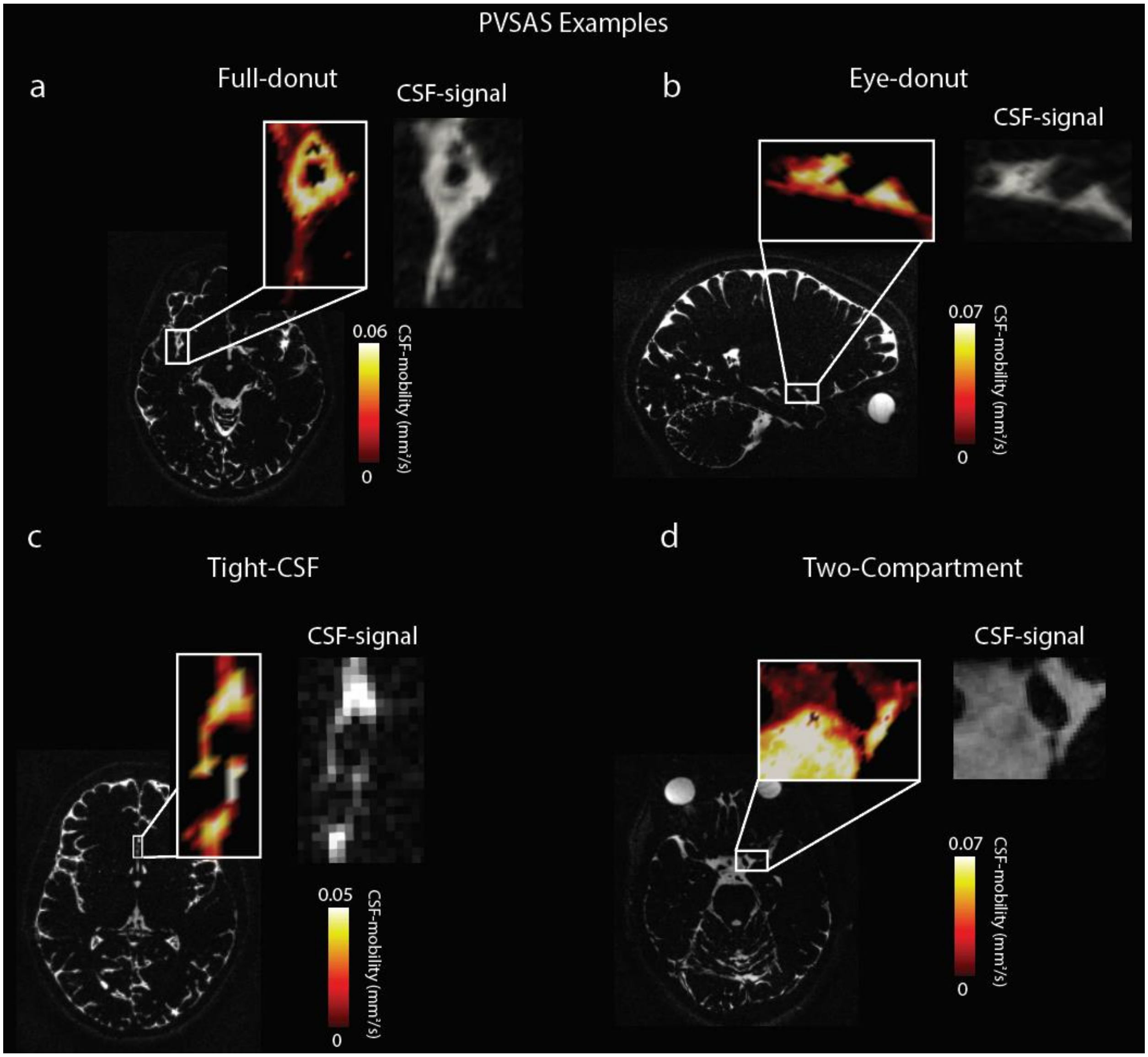

Supplemental Figure 3 | Examples of CSF structure across the anterior cerebral artery (ACA), M1 and internal carotid artery (ICA).

a) Full-donut perivascular subarachnoid space (PVSAS).
b) Eye-donut PVSAS.
c) Tight-CSF.
d) Two-compartment.

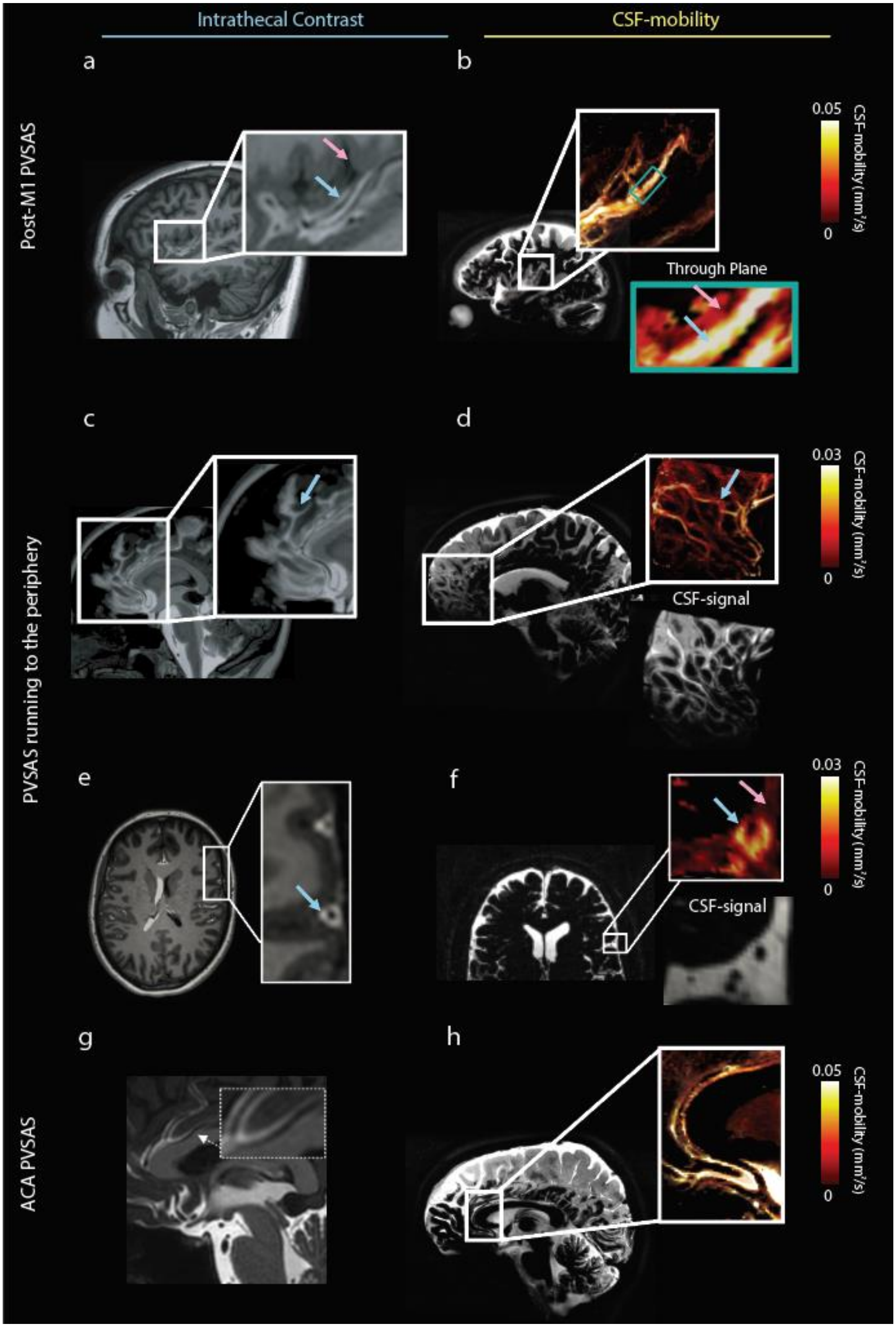

Intrathecal Contrast
CSF-mobility
a
b
Post-M1 PVSAS
0.05
0
CSF-mobility (mm²/s)
Through Plane
c
d
PVSAS running to the periphery
0.03
0
CSF-signal
e
f
0.03
0
CSF-signal
g
h
ACA PVSAS
0.05
0

Supplemental Figure 4 | Intrathecal and perivascular subarachnoid space (PVSAS) comparisons.

a) Intrathecal contrast agent travelling along the post-M1 shows contrast in the perivascular subarachnoid space (PVSAS) close to the vessel (blue arrow), and no contrast in the subarachnoid space (SAS) (pink arrow).
b) CSF-mobility of the post-M1 PVSAS. Shown in gray is a maximum-intensity projection (MIP) of the non-motion sensitized scan, and in the white box is the CSF-mobility, showing the MIP of post-M1 PVSAS. Green box shows cross sectional of post-M1 PVSAS show steep drop off between the PVSAS (blue) and the SAS (pink).
c) Intrathecal contrast agent scan shows contrast running to the periphery in the PVSAS (blue arrow).
d) CSF-mobility, showing PVSAS running to the periphery. Gray MIP of non-motion sensitized scan.
e) Intrathecal contrast agent PVSAS running to the periphery (blue arrow).
f) CSF-mobility PVSAS at the periphery (blue arrow) surrounded by SAS (pink arrow).
g) Intrathecal contrast agent runs along anterior cerebral artery (ACA) shows the PVSAS.
h) CSF-mobility 3D-representation of ACA shows limited SAS around the PVSAS.

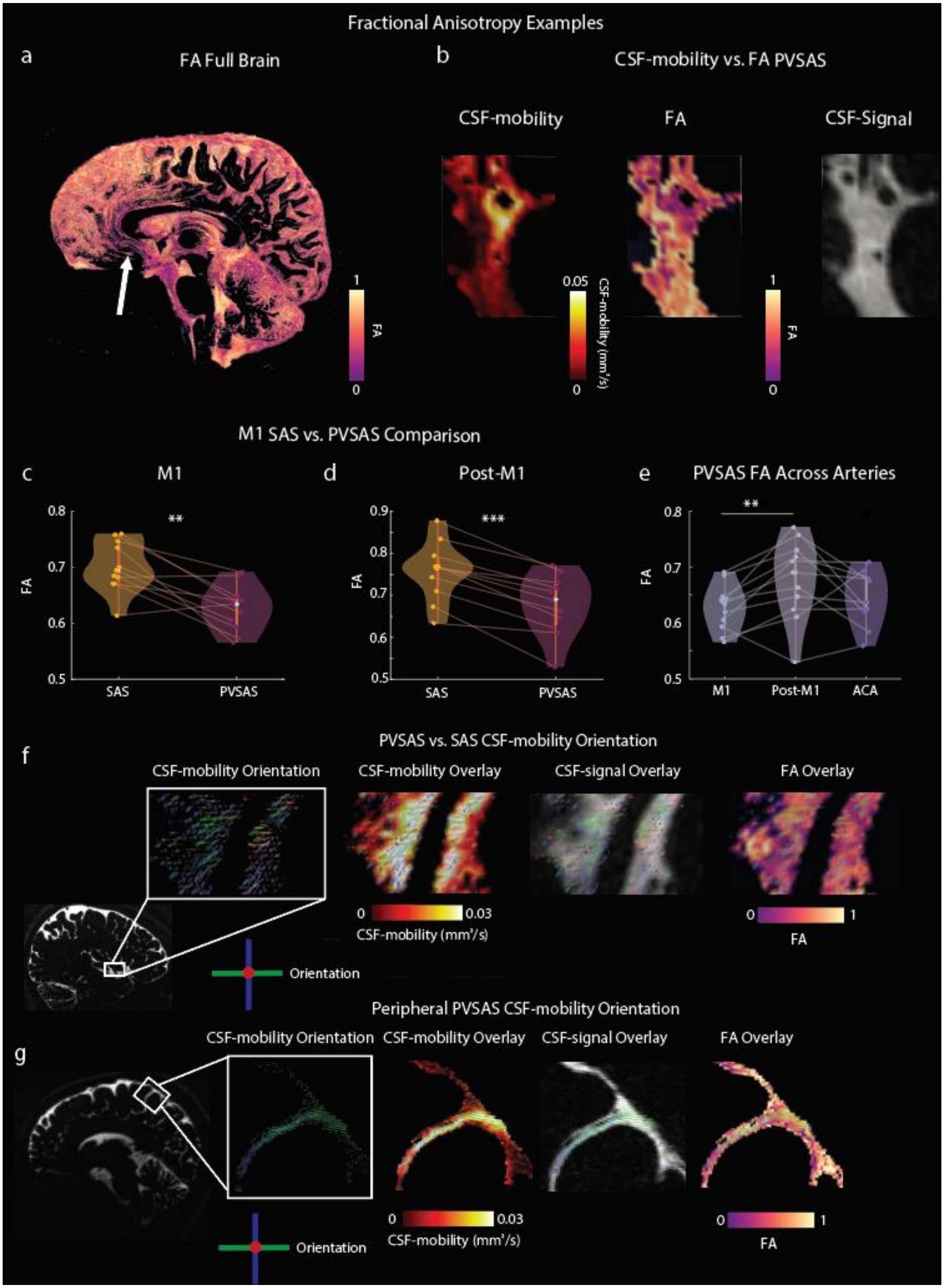

Fractional Anisotropy Examples
a
FA Full Brain
b
CSF-mobility vs. FA PVSAS
CSF-mobility
FA
CSF-Signal
1
FA
0
0.05
CSF-mobility (mm²/s)
0
1
FA
0
M1 SAS vs. PVSAS Comparison
c
M1
d
Post-M1
e
PVSAS FA Across Arteries
**
***
**
FA
SAS
PVSAS
M1
Post-M1
ACA
PVSAS vs. SAS CSF-mobility Orientation
f
CSF-mobility Orientation
CSF-mobility Overlay
CSF-signal Overlay
FA Overlay
0
0.03
CSF-mobility (mm²/s)
0
1
FA
Orientation
Peripheral PVSAS CSF-mobility Orientation
g
CSF-mobility Orientation
CSF-mobility Overlay
CSF-signal Overlay
FA Overlay
0
0.03
CSF-mobility (mm²/s)
0
1
FA
Orientation

Supplemental Figure 5 | Fractional anisotropy (FA) in perivascular subarachnoid space (PVSAS). Source data are provided as a Source Data file.

a) Sagittal full brain example of the fractional anisotropy (FA) shows decreased FA around the anterior cerebral artery (ACA).

b) Example of post-M1 PVSAS with CSF-mobility and matching FA maps. Decreased FA can be seen within the PVSAS with surrounding higher FA.

c/d) Violin plot comparing SAS vs. PVSAS FA values show statistically significant decrease in the PVSAS in the M1 (n=21 segments, 11 subjects, $p=1.2\times10^{-3}$, paired t-test, Bonferroni-corrected, 2 tests) and post-M1 (n=21 segments, 11 subjects, $p=2.73x10^{-5}$, two-sided paired t-test, Bonferroni-corrected, 2 tests).

e) Mean FA of PVSAS in the M1, post-M1, and ACA, indicate that there is a statistically significant increase in FA in the post-M1 compared to both the M1 and the ACA (n=21 segments, 11 subjects, M1 vs. post-M1 $p=6.75\times10^{-3}$, M1 vs. ACA p=0.37, post-M1 vs. ACA=0.13, two-sided paired t-test, Bonferroni-corrected, 3 tests).

f/g) Comparison between principal orientation of CSF-mobility, overlaid on the CSF-mobility, CSF-signal, and FA show that there is inconsistent FA in the PVSAS depending on if the FA is measured around the MCA or ACA (f), and peripheral PVSAS (g). Line colors indicate orientation: red=left-right, green=anterior-posterior, blue=superior-inferior.

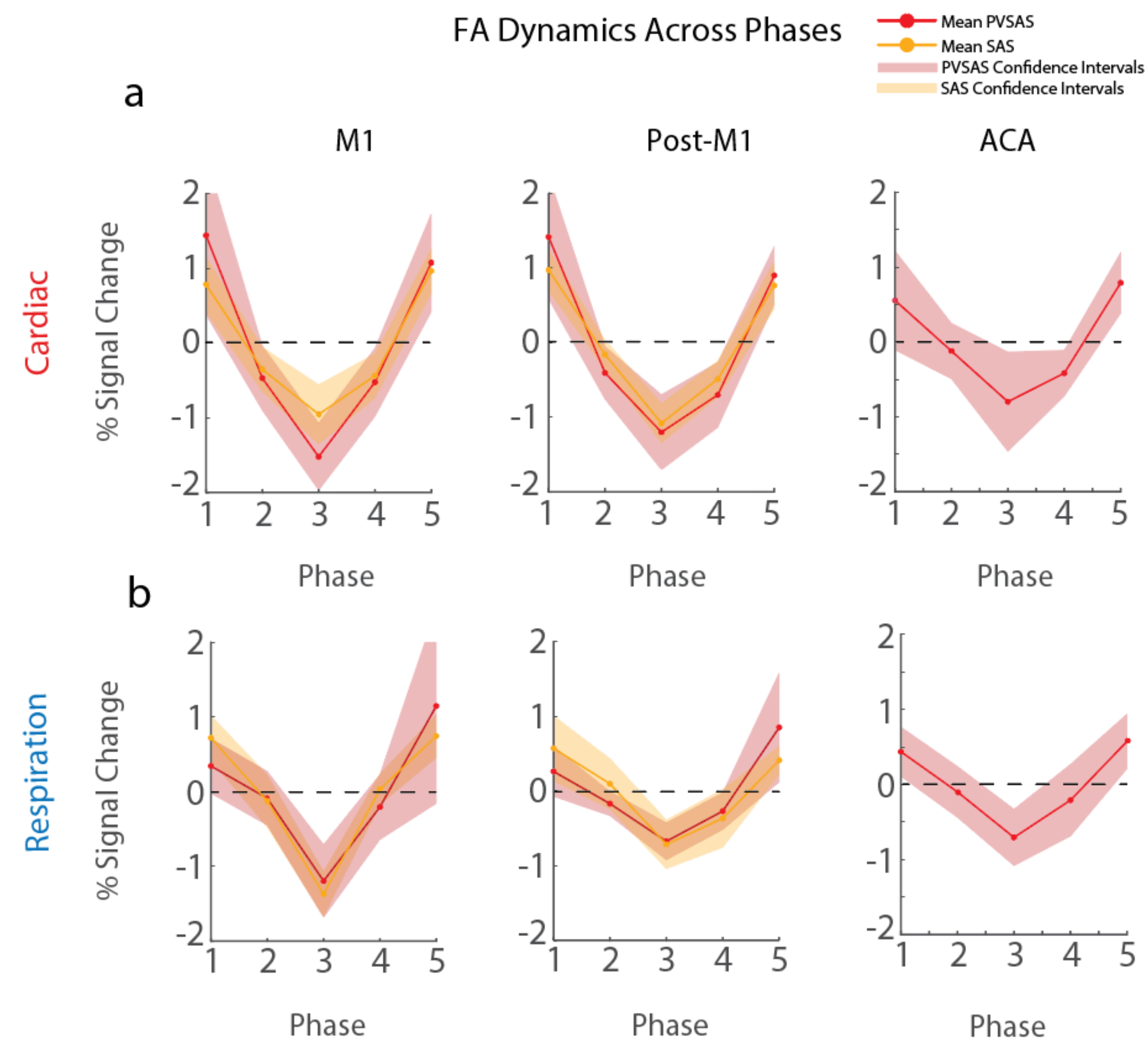

Supplemental Figure 6 | Cardiac and respiration modulate CSF-mobility and fractional anisotropy (FA) in the perivascular subarachnoid space (PVSAS) and the subarachnoid space (SAS). FA change from the mean value over phases (in %). Each line shows the mean over individuals and the shaded error areas represent confidence intervals of $SD \times 1.96/\sqrt{n}$ (n=11). Cardiac (a) and respiration (b) dynamics modulate M1, post-M1, and ACA PVSAS similarly across FA phases. There is no significant difference in the relative percent signal change when comparing the FA PVSAS to the FA SAS. Source data are provided as a Source Data file.

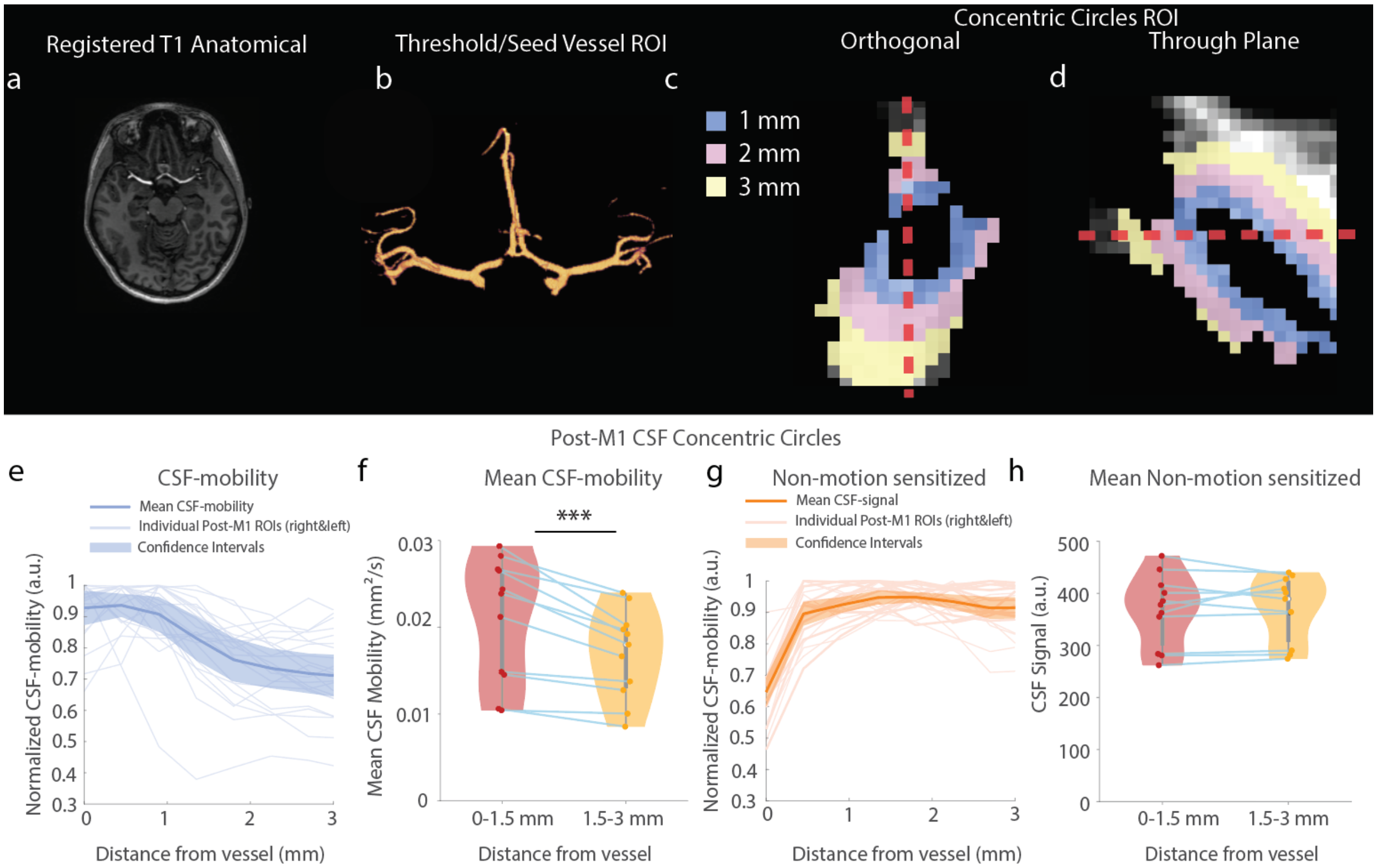

Supplemental Figure 7 | Concentric circles in the post-M1 region show distinct CSF-mobility in the perivascular subarachnoid space (PVSAS) compared to the subarachnoid space (SAS), while CSF-signal remains similar. Source data are provided as a Source Data file.

a) Inflow effect visible in the registered T1-weighted anatomical image used to create vessel mask.

b) Maximum intensity projection (MIP) of the vessel map after thresholding the T1-weighted image and placing a seed at the internal cerebral artery (ICA).

c/d) Concentric circles overlaid on the CSF-mobility scan representing distances of ~1 mm (blue), ~2 mm (pink), and ~3 mm (yellow) from the vessel wall. Red dotted line indicates corresponding orthogonal/through-plane cut.

e) Normalized CSF-mobility stepping away from the vessel shows high CSF-mobility with a steep drop-off at ~1.5 mm to low CSF-mobiliFty. Light blue lines indicate individual post-M1 ROIs (right and left for each subject). Dark blue line indicates average. The shaded blue error area represent confidence intervals of SD×1.96/√n (n=11).

f) Mean CSF-mobility (0-1.5 mm vs. 1.5-3 mm) shows significantly higher CSF-mobility where the PVSAS is expected (11 subjects, post-M1 $p=6.0\times10^{-4}$, two-sided paired t-test, Bonferroni-corrected, 2 tests).

g) Normalized non-motion-sensitized CSF-signal as a function of distance from the vessel. A partial volume effect influences the CSF-signal close to the vessel independently of CSF-mobility,

with no systematic decrease observed with increasing distance. Light orange lines indicate individual post-M1 ROIs (right and left for each subject). Dark orange line indicates average. The shaded orange error area represent confidence intervals of SD×1.96/√n (n=11).

h) Mean CSF-signal (0-1.5 mm vs. 1.5-3 mm) shows no significant change in CSF-signal where the PVSAS is expected (11 subjects, post-M1 p=0.74, two-sided paired t-test).

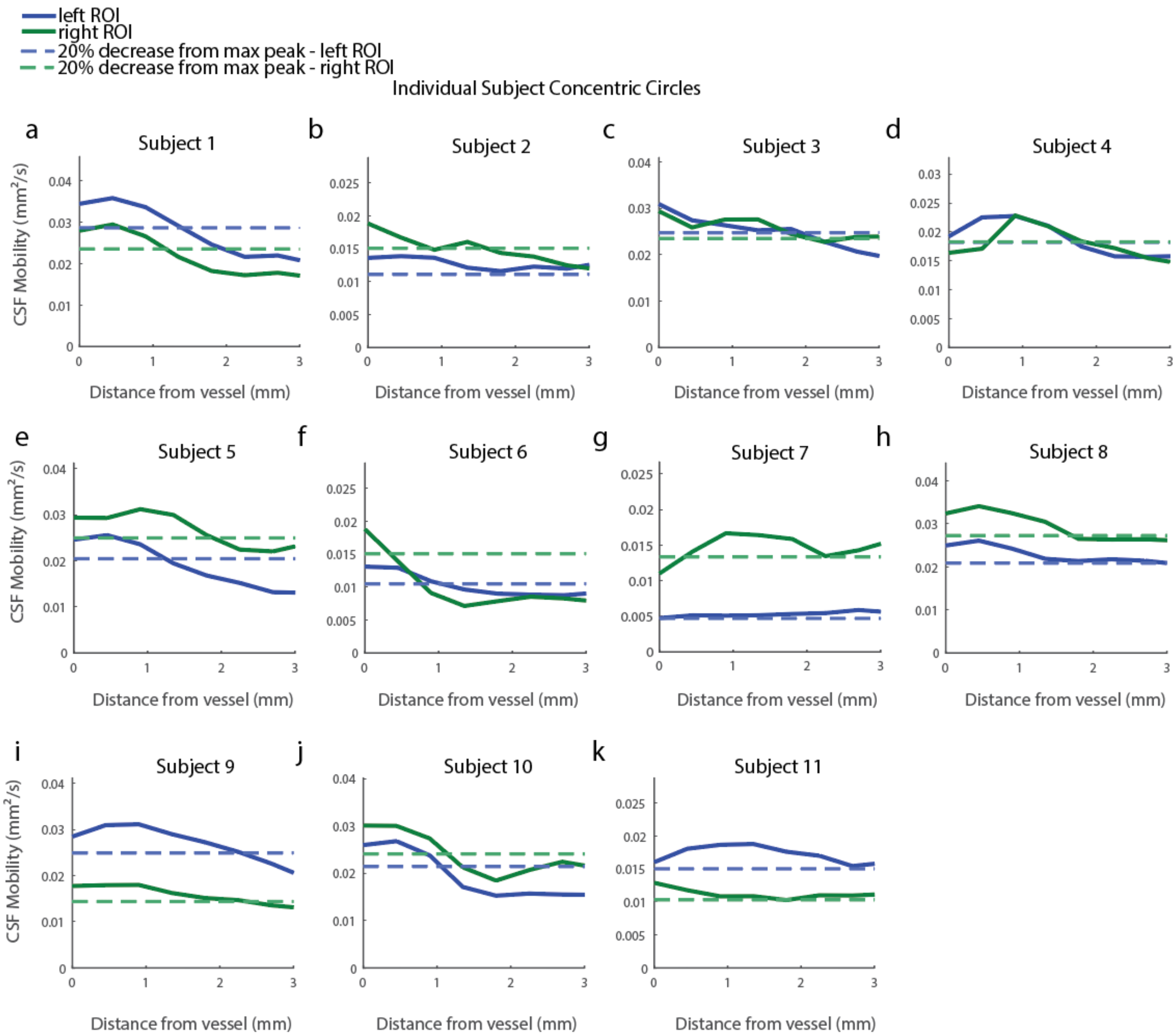

Supplemental Figure 8 | Individual subject concentric circles to visualize the perivascular subarachnoid space (PVSAS) in the post-M1 region. CSF-mobility along concentric circles in the post-M1 PVSAS for individual subjects (a-k). Traces show mean CSF-mobility as a function of distance from the vessel wall for the left (blue) and right (green) hemispheres. Dashed lines indicate the 20% drop threshold from the peak CSF-mobility values for each hemisphere. Source data are provided as a Source Data file.

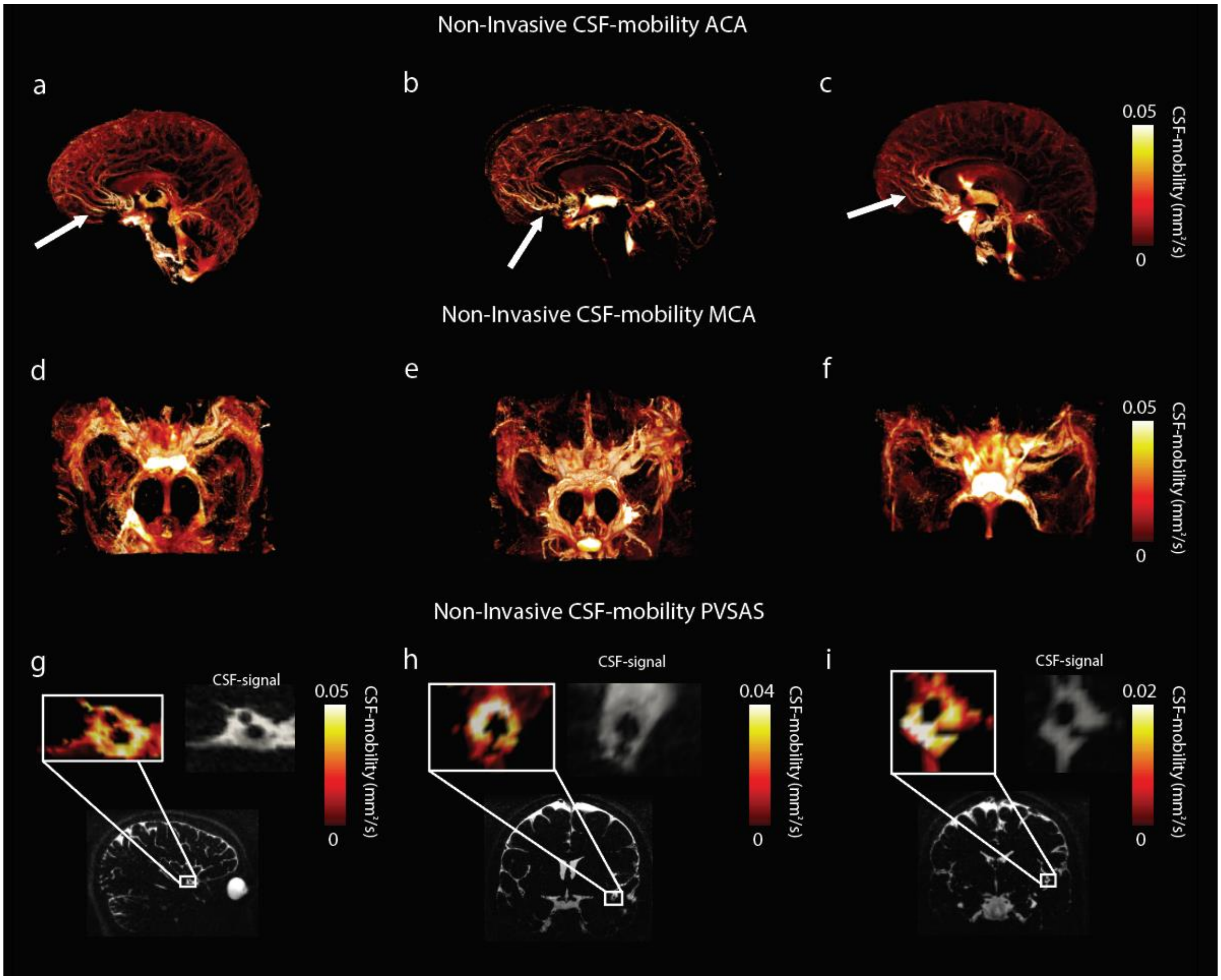

Supplemental Figure 9 | Additional examples of CSF-mobility perivascular subarachnoid space (PVSAS).

a-c) Additional sagittal views of CSF-mobility scan in healthy controls show perivascular subarachnoid space (PVSAS) surrounding the anterior cerebral artery (ACA). White arrows highlight ACA.

d-f) Transverse views of CSF-mobility scan show high CSF-mobility PVSAS around the middle cerebral artery (MCA).

g-i) Examples of full-donut PVSAS with high CSF-mobility surrounding post-M1 arteries, next to matching non-motion-sensitized scans (gray). White boxes mark regions used for PVSAS extraction; dotted lines indicate locations of the corresponding through-plane images in second panel.
