# Supplementary material for: Non-invasive characterization of perivascular subarachnoid spaces
Source: Nat Commun. 2026 Aug 5;17:9496. doi: 10.1038/s41467-026-76306-9 (PMC13542271; doi:10.1038/s41467-026-76306-9)
Supplement: Supplementary file 2 [file 41467_2026_76306_MOESM2_ESM.pdf]

Corresponding author(s): Dr. Lydiane Hirschler, Nina Fultz

Last updated by author(s): Jun 23, 2026

Reporting Summary

Nature Portfolio wishes to improve the reproducibility of the work that we publish. This form provides structure for consistency and transparency in reporting. For further information on Nature Portfolio policies, see our Editorial Policies and the Editorial Policy Checklist.

Statistics

For all statistical analyses, confirm that the following items are present in the figure legend, table legend, main text, or Methods section.

n/a	Confirmed	
☐	☒	The exact sample size (n) for each experimental group/condition, given as a discrete number and unit of measurement
☐	☒	A statement on whether measurements were taken from distinct samples or whether the same sample was measured repeatedly
☐	☒	The statistical test(s) used AND whether they are one- or two-sided<br>*Only common tests should be described solely by name; describe more complex techniques in the Methods section.*
☒	☐	A description of all covariates tested
☐	☒	A description of any assumptions or corrections, such as tests of normality and adjustment for multiple comparisons
☐	☒	A full description of the statistical parameters including central tendency (e.g. means) or other basic estimates (e.g. regression coefficient) AND variation (e.g. standard deviation) or associated estimates of uncertainty (e.g. confidence intervals)
☐	☒	For null hypothesis testing, the test statistic (e.g. F, t, r) with confidence intervals, effect sizes, degrees of freedom and P value noted<br>*Give P values as exact values whenever suitable.*
☒	☐	For Bayesian analysis, information on the choice of priors and Markov chain Monte Carlo settings
☒	☐	For hierarchical and complex designs, identification of the appropriate level for tests and full reporting of outcomes
☒	☐	Estimates of effect sizes (e.g. Cohen's d, Pearson's r), indicating how they were calculated

Our web collection on statistics for biologists contains articles on many of the points above.

Software and code

Policy information about availability of computer code

Data collection	MATLAB 2020b
Data analysis	Elastix version 4.9.043, ParaView 5.13.2, Paraview 6.1.0, ITK-SNAP 4.2.2, MATLAB 2020b. Code can be found on: https://github.com/ninafultz/csf_donuts, and archived via Zenodo (DOI: 10.5281/zenodo.20703876).

For manuscripts utilizing custom algorithms or software that are central to the research but not yet described in published literature, software must be made available to editors and reviewers. We strongly encourage code deposition in a community repository (e.g. GitHub). See the Nature Portfolio guidelines for submitting code & software for further information.

Data

Policy information about availability of data

All manuscripts must include a data availability statement. This statement should provide the following information, where applicable:

- Accession codes, unique identifiers, or web links for publicly available datasets
- A description of any restrictions on data availability
- For clinical datasets or third party data, please ensure that the statement adheres to our policy

Source data are available via Zenodo (DOI: 10.5281/zenodo.20744794), and source data are provided as a Source Data file with this paper.

Research involving human participants, their data, or biological material

Policy information about studies with human participants or human data. See also policy information about sex, gender (identity/presentation), and sexual orientation and race, ethnicity and racism.

Reporting on sex and gender	CSF-STREAM healthy controls: sex: 9 females, 3 males; no gender information was collected. No sex or gender analyses was done because of the small sample size.<br>Intrathecal patient cohort: 8 females, 2 males; no gender information was collected. No sex or gender analyses was done because of the small sample size.
Reporting on race, ethnicity, or other socially relevant groupings	We did not collect information on race, ethnicity or any other socially relevant groupings.
Population characteristics	Because of our small sample size, we did not do any additional analysis on population characteristics.
Recruitment	CSF-STREAM healthy control cohort: Participants were recruited through a hospital database.<br>Intrathecal patient cohort: Consecutive patients referred to the Department of neurosurgery, Oslo university hospital - Rikshospitalet, Oslo, Norway, for various CSF disorders were included. The indication for intrathecal contrast enhanced MRI was made on clinical reasons. The inclusion criteria were clinically suspected cerebrospinal fluid disturbance. Exclusion criteria were evidence of renal dysfunction, allergy towards contrast agents, severe allergy in general, pregnant or breast feeding women, age <18 years /80 years.
Ethics oversight	CSF-STREAM healthy controls: The study was conducted in compliance with the Leiden University Medical Center Institutional Review Board (Leiden, The Netherlands) under authorization number P07.096.<br>Intrathecal patient data: The study was approved by the Institutional Review Board (2015/1868), Regional Ethics Committee (2015/96) and the National Medicines Agency (15/04932-7), and registered in Oslo University Hospital Research Registry (ePhorte 2015/1868). All participants gave informed and written consent, and they received no compensation.

Note that full information on the approval of the study protocol must also be provided in the manuscript.

Field-specific reporting

Please select the one below that is the best fit for your research. If you are not sure, read the appropriate sections before making your selection.

[x] Life sciences [ ] Behavioural & social sciences [ ] Ecological, evolutionary & environmental sciences

For a reference copy of the document with all sections, see nature.com/documents/nr-reporting-summary-flat.pdf

Life sciences study design

All studies must disclose on these points even when the disclosure is negative.

Sample size	CSF-STREAM: No sample size calculation was performed, however, previous studies looking at CSF dynamics have used similar population sizes (Fultz et al., 2019), and so we expected n=11 to be sufficient.<br>Intrathecal patient data: We used n=10 patients, and we expect this population size (Fultz et a., 2019) to be sufficient for look at CSF-dynamics.
Data exclusions	CSF-STREAM: Twelve healthy individuals participated in the study. Data of one participant was excluded because of motion artifacts.<br>Intrathecal patient data: Ten subjects were pulled from a larger cohort of subjects as comparison images.
Replication	We have now reproduced our CSF-STREAM PVSAS findings at 3 Tesla and with follow-up studies. We see the PVSAS consistently in healthy controls. Follow-up studies have shown similar PVSAS structures in a range of healthy controls using CSF-STREAM (presented as abstracts: Engels et al., ISMRM Workshop on Fluids, Flows, and Clearance in the Brain: What Can we Image and How Should It be Interpreted 2026?; Svensson et al., ISMRM 2026).
Randomization	This is not relevant to the study because there was no groups to randomize to.
Blinding	Blinding was not relevant because we were assessing anatomical and functional dynamics in healthy controls, or using intrathecal data from patients, and not comparing disease states.

Reporting for specific materials, systems and methods

We require information from authors about some types of materials, experimental systems and methods used in many studies. Here, indicate whether each material, system or method listed is relevant to your study. If you are not sure if a list item applies to your research, read the appropriate section before selecting a response.

Materials & experimental systems

n/a	Involved in the study
☒	☐ Antibodies
☒	☐ Eukaryotic cell lines
☒	☐ Palaeontology and archaeology
☒	☐ Animals and other organisms
☒	☐ Clinical data
☒	☐ Dual use research of concern
☒	☐ Plants

Methods

n/a	Involved in the study
☒	☐ ChIP-seq
☒	☐ Flow cytometry
☐	☒ MRI-based neuroimaging

Plants

Seed stocks

Report on the source of all seed stocks or other plant material used. If applicable, state the seed stock centre and catalogue number. If plant specimens were collected from the field, describe the collection location, date and sampling procedures.

Novel plant genotypes

Describe the methods by which all novel plant genotypes were produced. This includes those generated by transgenic approaches, gene editing, chemical/radiation-based mutagenesis and hybridization. For transgenic lines, describe the transformation method, the number of independent lines analyzed and the generation upon which experiments were performed. For gene-edited lines, describe the editor used, the endogenous sequence targeted for editing, the targeting guide RNA sequence (if applicable) and how the editor was applied.

Authentication

Describe any authentication procedures for each seed stock used or novel genotype generated. Describe any experiments used to assess the effect of a mutation and, where applicable, how potential secondary effects (e.g. second site T-DNA insertions, mosiacism, off-target gene editing) were examined.

Magnetic resonance imaging

Experimental design

Design type

Resting state

Design specifications

CSF-STREAM healthy controls cohort: One scan was done per person, with each scan being 38 minutes and 30 seconds. Intrathecal patient comparison: Scans were acquired continuously during the first hour following intrathecal injection, then repeated two hours post-injection.

Behavioral performance measures

No task was done.

Acquisition

Imaging type(s)

CSF-STREAM healthy controls cohort: functional; Intrathecal patient comparison: T1 MRI anatomical

Field strength

CSF-STREAM healthy controls cohort: 7 Tesla; Intrathecal patient comparison: 3T

Sequence & imaging parameters

CSF-STREAM healthy controls cohort: High-resolution, whole-brain (0.45 mm isotropic voxel-size, field-of-view = 250×250×190
mm), 3D images were acquired with a TSE sequence (TE = 495 ms, TR = 3.4 s, TSE-factor 146, excitation
& refocusing FA = 90∘)
Intrathecal patient comparison: All scans were acquired on a 3 Tesla MRI scanner (Ingenia, Philips Medical Systems) using a 32-channel head coil. Sagittal 3D T1-weighted volumes were acquired using the following parameters: TR/TE = shortest available (typically 5.1/2.3 ms), flip angle = 8°, FOV = 256 × 256 mm, acquisition matrix = 256 × 256 (reconstructed to 512 × 512). A total of 184 overlapping 1-mm slices were acquired and automatically reconstructed to 368 slices at 0.5-mm thickness.

Area of acquisition

Whole-brain

Diffusion MRI ☐ Used ☒ Not used

Preprocessing

Preprocessing software

CSF-STREAM healthy controls cohort: Reconstructions were done using MATLAB 2018b (The Mathworks, USA), using an in-house built reconstruction pipeline developed within ReconFrame (GyroTools, Zürich, Switzerland) in combination with the open-source Berkeley Advanced Reconstruction
Toolbox (BART)40 version 0.4.03.

Normalization

Not performed.

Normalization template

Not performed.

Noise and artifact removal | CSF-STREAM healthy controls cohort: One subject was removed because of motion.

Volume censoring | Not performed.

Statistical modeling & inference

Model type and settings | No models were used.

Effect(s) tested

To compare the distributions of CSF-mobility shapes around different vessels, we performed Kruskal-Wallis tests to assess whether there were significant differences across multiple shape categories for the ACA, MCA, and ICA. This non-parametric test was chosen due to the ordinal nature of the shape ratings and the potential for non-normal distributions. If the Kruskal-Wallis test indicated a significant difference ($p \leq 0.05$), we conducted post-hoc pairwise Wilcoxon rank-sum tests with Bonferroni correction to identify specific group differences.

Paired two-tailed t-tests with Bonferroni correction were done to compare the mean CSF-mobility and CSF-signal on post-M1 and M1 SAS and PVSAS ROIs as described above. Paired two-tailed t-tests with Bonferroni correction were done to compare the mean CSF-mobility and fractional anisotropy on post-M1 and M1 and ACA PVSAS ROIs as described above.

Paired two-tailed t-tests with Bonferroni correction were done on mean concentric circle values between 0-1.5 mm vs. 1.5-3 mm.

Specify type of analysis: ☐ Whole brain ☒ ROI-based ☐ Both

Anatomical location(s) | Manual CSF-STREAM healthy control ROIs were extracted around major arteries (MCA, ACA).

Statistic type for inference | Not applicable.

(See Eklund et al. 2016)

Correction | Multiple comparisons Bonferonni correction was done for all tests.

Models & analysis

n/a	Involved in the study
☒	☐ Functional and/or effective connectivity
☒	☐ Graph analysis
☒	☐ Multivariate modeling or predictive analysis
