# Supplementary material for: Non-invasive characterization of perivascular subarachnoid spaces
Source: Nat Commun. 2026 Aug 5;17:9496. doi: 10.1038/s41467-026-76306-9 (PMC13542271; doi:10.1038/s41467-026-76306-9)
Supplement: Supplementary file 3 [file 41467_2026_76306_MOESM3_ESM.pdf]

nature portfolio

Peer Review File

Non-invasive Characterization of Perivascular Subarachnoid Spaces

Corresponding Author: Ms Nina Fultz

This file contains all reviewer reports in order by version, followed by all author rebuttals in order by version.

Version 0:

Reviewer comments:

Reviewer #1

(Remarks to the Author)
This is an exciting and impactful study that seeks to compare two recent cutting edge neuroimaging data sets to answer important questions concerning the anatomy and function of paravascular CSF transport in the human brain.

To do this, the authors take an innovative approach of visually assessing the spatial patterns of the CSF motility maps generated from the CSF-STREAM technique. By doing so they observe striking similarities to regions of contrast enhancement (from the invasive contrast-enhanced approach using intrathecal delivery of gad) thought to represent para-arterial spaces.

They then characterise spatial patterns of CSF motility in more downstream para-arterial locations (which cannot be easily visualised with the invasive contrast enhanced approach). As such, this novel approach fulfils the title of the work to better characterize PVS spaces in the human brain using a non-invasive approach. It also provides evidence that the CSF-STREAM approach at 7T can map pararterial CSF spaces in healthy volunteers, potentially representing a novel biomarker for early detection of neurologic conditions. Thus, this work would be a very useful addition to the literature.

I have the following critiques:

Major comments:

Generally, the clarity of the paper could be significantly improved. Despite being familiar with the two papers whose comparison form the central thesis of the work (references 10 and 11), I had to read the paper multiple times to understand what the hypothesis were that were being tested and what data analysis approach was used to investigate them.

Therefore, the authors should clarify what were the precise questions that they were seeking to answer from their analysis and what was the analysis approach that they took to answer these questions. In order to achieve this I recommend having separate sub-headings for each of the questions that match between the aims/ results and methods and figures so that this process is more clear to the reader.

This is important, as when you have two large multi-dimensional data sets as is the case here (CSF-STREAM & dynamic contrast enhanced), there are so many possible metrics that you could extract from the data that the analysis approach should carefully consider subjective bias and multiple comparisons etc. Ideally exploratory analysis should be performed, hypotheses generated and then tested on a separate data set. The reader may be sceptical, for example as often just a single intrathecal data set is shown, to what extent subjective bias has come into the choice of data shown and to what extent the analysis is circular if the same data has been used to generate the hypothesis.

The whole brain comparisons are v interesting and reveal striking similarities. This is shown elegantly in Figure 1 (a,b,d,e). However I am unclear where Figure 1(g,h) fits into this comparison as these are not referenced in the text until much later so it would help clarity if these sections were moved to a later figure. In addition, the whole brain comparison is substantiated by just 1 data set for each modality (and it is not clear how these were chosen). It is fine to have a representative example, but more whole brain comparison data sets should be given in the supplementary material in order to provide more evidence to substantiate the apparent similarity between the whole brain maps.

It is my understanding that a critical component of the work is the comparison of paraterial spaces captured with the contrast enhanced methods vs the CSF motility maps from CSF stream. I believe that the aim of Figure 2(a-c) and Figure 1(c,f) is to make this comparison and in turn to present evidence that the CSF motility maps from the CSF-stream technique can detect the same para-arterial spaces but non-invasively – this is an important finding. However, these data are not presented very clearly or with enough subjects. It would really help the comparison if the data from both techniques is presented in the same format (ie have a zoomed out version from both methods and a zoomed in image with the same FOV showing the equivalent vessel). Thus a lot more space in the Figures should be dedicated to this important comparison. The though plane images (and cross sectional CSF motility plots) from the CSF stream are interesting and could be included as an additional figure/supplementary but there are not equivalent though plane images in the contrast enhanced data so this complicates the comparison. Similar to my comments to figure 1, again as the evidence is primarily based on visual comparisons with n=1, more examples should be given in the supplementary material to substantiate the authors conclusions.

The classification of the CSF mobilty maps into different shapes (ie 'full-donut, tight-CSF, eye-donut, and two-compartment) is very interesting. Can the authors include more of the raw data in the supplementary material so the reader can get a better feel for these data? How was subjective bias minimised in this analysis?

Can the authors comment on whether the FA maps give and insight into pararterial structure and how this compares to the ability of the CSF motility maps to do so. Did the authors also consider applying their PVS classification approach to the CAA patients in reference 11?

Minor comments:

I understand that the authors have referenced their previous paper for detailed methods etc but a few more details to explain what their measure of CSF motility is measuring are necessary as the more general reader will be confused that CSF motility is measured in mm2/s.
'Alternative barriers, such as the subarachnoid lymphatic-like membrane (SLYM) 13,14' – tweak wording to acknowledge current uncertainty in this SLYM (https://www.science.org/doi/10.1126/science.adc8810#elettersSection).

Abstract: Using non-invasive, high-resolution imaging in healthy adults, we observed high CSF-mobility around both proximal and distal cerebral arteries and showing that perivascular subarachnoid spaces (PVSAS) have location-specific structures. – grammar

'These findings suggest that CSF flow may be more spatially distinct than previously thought, providing a foundation for understanding fluid dynamics in health and disease.' – I'm unclear about the rational of this statement as the intratheacle data set which has already been published has already demonstrated that CSF flow is spatially distinct around the paraarterial space vs the subarachnoid space.

' we observed high CSF-mobility around both proximal and distal cerebral arteries and showing that perivascular subarachnoid spaces (PVSAS) have location-specific structures' the data is measures of PVS motility and thus you are not actually measuring structure. Change the wording to clarify this distinction between what you are actually measuring and what you are inferring.

Intro:
'. Based on the previous intrathecal contrast agent work, we hypothesized 86 that the PVSAS would have higher CSF-mobility around branches of the MCA and ACA (M1-M4, A1- 87 A4)' – hypothesis is incomplete – higher then what? What are you comparing to?

Intro: 'However, recent research challenges the traditional view of the subarachnoid space (SAS), which harbors the CSF, as a single continuous space' need a reference to support this statement.
'However, these observations were made in patients undergoing intrathecal injections as part of their diagnostic work-up, i.e. with suspicion of a CSF disorder' -specify that you mean injection of contrast agent.

It may be worth mentioning in the introduction that a key element of the previous study (reference 10) was that the signal in the para-arterial space correlated to tissue delivery.
'Intrathecal contrast agent-based detection of PVSAS is very time-sensitive: if the scan is performed too early, the contrast agent has not yet arrived; if scanned too late, it has already spread throughout the entire SAS.' Slightly disingenuous/confusing statement as this is the idea behind the method – it is a dynamic scan and you can get useful information by analysis the time-dependence of the data. I would rephrase this.

'The presence of distinct, high-mobility perivascular regions in the absence of pathology raises important questions about their role in circulating CSF on the driving forces of the water movement in this compartment as well as throughout the complete brain.' -grammar.

(Remarks on code availability)

Reviewer #2

(Remarks to the Author)
Recent work has suggested that CSF flow is not homogeneous within the SAS, but may instead follow specialized perivascular subarachnoid space (PVSAS) pathways. Building on intrathecal contrast studies, the present work employed CSF-STREAM, a non-invasive, high-resolution MRI technique that isolates CSF signal and maps CSF mobility, to determine whether PVSAS can be visualized in healthy individuals. In 11 participants, CSF-mobility maps revealed consistent high-mobility regions adjacent to major cerebral arteries, particularly along the MCA (M1–M4) and ACA (A1–A4), closely paralleling patterns reported in intrathecal imaging. Cross-sectional analyses demonstrated "donut-like" perivascular profiles, with significantly elevated CSF mobility in the PVSAS relative to surrounding SAS. Distinct morphological patterns ("full-donut," "tight-CSF," "eye-donut," and "two-compartment") were classified across arterial segments, with the full-donut configuration most prominent in distal MCA branches. Additional observations near the ICA bifurcation and superior sagittal sinus suggested further heterogeneity of perivascular compartments. These findings demonstrate that CSF-STREAM can non-invasively detect and characterize PVSAS in vivo, providing evidence for anatomically distinct, high-mobility CSF pathways in the healthy human brain. This study is novel and contributes meaningfully to our understanding. By using a non-invasive, tracer-free MRI approach (CSF-STREAM), it demonstrates for the first time that PVSAS can be visualized and quantified in healthy individuals, not just in patients undergoing intrathecal contrast studies. The identification of distinct morphological patterns of CSF mobility around major cerebral arteries provides new evidence that CSF transport is regionally organized rather than uniform. This advances the concept that perivascular compartments are functional conduits for CSF dynamics, with implications for brain clearance and immune surveillance. Collectively, the findings broaden our mechanistic framework for how CSF flows through the human brain and open opportunities for studying its role in aging and disease. I have no additional comments

(Remarks on code availability)
the GitHub repository cited at the bottom of the first page was not available to view. there was no repository in this account named as "csf_donuts".

Reviewer #3

(Remarks to the Author)
This study examines CSF mobility within the perivascular subarachnoid space (PVSAS) of the human brain, reporting that mobility is higher around major arteries. While the findings are consistent with prior knowledge and physiologically plausible, the analyses remain limited and overly simplified. As such, they primarily confirm known phenomena without adding much new insights into CSF circulation or physiology. The lack of novel mechanistic understanding and insufficient depth of analysis constrain the overall significance and impact of the work. Major comments are outlined below:

1. It is unclear whether CSF mobility measures provide any information about the direction of CSF movement. The observed higher mobility may simply reflect signal fluctuations caused by arterial size changes, rather than genuine CSF/glymphatic flow. Without direct evidence of directional CSF flow along arteries, the current results risk being dismissed as partial volume artifacts or pulsation-induced noise rather than meaningful physiological transport.

2. Because CSF dynamics are already known to be tightly coupled to arterial pulsatility, the finding of higher mobility around arteries is expected and adds little novelty. What would be more compelling is a quantitative analysis relating CSF mobility to arterial size and pulsatility, ideally through systematic mapping and segmentation of both large and small arteries.

3. Figures 2 and 3 present variations in the shapes and appearances of donut-like PVSAS structures, but the physiological significance of these observations is not explained. Do these differences simply reflect structural variation in arterial morphology and surrounding tissues, or are they meant to indicate functional aspects of CSF dynamics? As presented, the motivation, hypothesis, and key message of these results are vague.

(Remarks on code availability)

Reviewer #4

(Remarks to the Author)
I read with interest the study by Fultz and colleagues. It leverages an MRI-method developed by their group to measure CSF motion in the subarachnoid space. They show that CSF flow is predominantly alongside certain neuro-anatomical pathways, also in healthy individuals. The manuscript is well written, has convincing illustrations and is of interest to the CSF community (and beyond). I believe the manuscript could further benefit from amending additional details on methodology and more clearly claiming an exploratory nature. Find my detailed comments below.

I would briefly mention earlier in the main how many participants were included in your study.

Please also briefly mention spatial and time resolution of CSF-STREAM. This is important information to put your findings into context.

"In both modalities, sagittal views show prominent signal in the basilar cistern, indicating fast contrast spreading and high CSF-mobility" – this is a bit confusing. CSF-STREAM does not rely on contrast agent; or do you compare with former

experiments including contrast (in non-healthy individuals)? Please clarify.

“Mean CSF-mobility shows an increase in the PVSAS compared to the SAS (Figure 2e, p=2.0x10-6104 , paired t-test, Bonferroni-corrected).” – please add n per group here as well. Also, I’d recommend that you add the boxplots for these two groups with individual data points to the supplementary data. This also applies to other statistical test values you report. Also, please specify in the methods how many tests you ran.

“When examining the CSF-mobility by visual inspection in eleven healthy subjects, we consistently saw four distinct patterns related to PVSAS that we classified based on spatial distribution and CSF-mobility changes: full-donut, tight-CSF, eye-donut, and two-compartment (Figure 3m,n).” – can you please add some actual imaging examples to strengthen this point further.

Have you performed a sample-size calculation? Please specify. Alongside this, it should be mentioned more clearly that this is an exploratory study, given the relatively small sample size and the absence of an a priori study protocol (I assume? Correct me if I am wrong).

More minor comments:
The text would benefit from subtitles (if the journal allows this) to make it more reader-friendly
Please make sure to make your Matlab code for statistical analysis available alongside your paper so other researchers are able to reproduce your findings (or re-use your code for their studies). I clicked on the link and it led to an error (maybe the repository is not set to public?)

I sign my review: Benjamin Ineichen, Department of Clinical Research, University of Bern, Switzerland

(Remarks on code availability)
Link did not work (repository set to private?)

Version 1:

Reviewer comments:

Reviewer #1

(Remarks to the Author)
Overall, the authors have done a good job addressing my previous concerns. The rationale, methodology and findings are now much clearer. The findings presented here are very interesting and would be a welcome addition to the literature where there is a need for novel and non-invasive methods to study CSF-ISF exchange in the human brain.

Major comment:

For me, the most impactful finding of this work is that the CSF STREAM motility maps spatially agree with the para-arterial channels that preferentially transport the gad tracer identified from the intrathecal contrast-enhanced data sets. An important aspect of this observation is that the CSF STREAM motility maps and not just the CSF images spatially correlate to the DCE data. This concept is well illustrated in Figure 2F vs Figure 2G (where the ‘CSF signal’ images are also shown and do not spatially correlate as well to the DCE images).

If this summation is correct, the CSF STREAM method could be used to map functionally relevant paravascular pathways non-invasively (and not just a long-TE high res scan to map paravascular spaces [ie the ‘CSF signal’ images]).

I can see that the authors have added additional data in support of this conclusion (e.g supplementary Figure 3) but I still feel that showing more comparative data from more subjects using the same format in e.g. Figure 2F vs Figure 2G would help convince readers as to the robustness of this finding. As currently, the reader may be sceptical about this finding if only data from 2-3 subjects of the ~11 in each group are shown in the paper.

Directly related to this, the way the data from the two types of scans are compared could be more consistent. For example, in Figure 2 b it is coronal and in Figure 2 C is sagittal and in Figure 3 the ICA comparison is missing. As I described in my initial review it would be helpful if the CSF STREAM data was compared to the DCE data using the same orientation and field of view in the ‘zoom in’ images so that the reader can more easily visually compare the images.

Minor comments:
The discussion is currently quite brief and could be more detailed in places:
It would be helpful to explain exactly what is measured by the method (ie why is motility reported in mm2/s) as those unfamiliar with the history of the method may be confused by this.
To my understanding the CSF-STREAM is quite a long acquisition. Could the authors recommend an acquisition scheme to quickly capture the motility data that spatially correlates with the paraarterial locations identified on the DCE scans ie if you do not have to precise measure the FA/ tensor and just ‘ADC’ could you make the scan quicker?

‘‘in comparison with the post-M1 PVSAS. Beyond the major vascular tree, the peripheral high-mobility PVSAS were found heterogeneously around both arteries and veins and only seen in a minority of subjects.’ Could the authors reference the appropriate results/Figures that support this statement and/or explain in more detail what evidence led to this summation as, for me, it is currently quite unclear.

For the data depicted in Figure 6, it should be made more clear that this was only observed in 2 of 11 subjects and therefore is not a robust observation. In the discussion could the authors discuss whether they think this was physiological or methodological?

Could the authors discuss the evidence for bulk vs diffusive motion of para-arterial fluid?

(Remarks on code availability)

Reviewer #2

(Remarks to the Author)

(Remarks on code availability)

Reviewer #3

(Remarks to the Author)
Here are some of my remaining comments:
The major claim of the paper is the observation of higher mobility in the PVSAS compared with the surrounding SAS using the CSF stream method, which is stated to be consistent with previous findings from contrast-enhanced studies investigating PVSAS and the PVSAS membrane. However, the comparison between the CSF stream method and the contrast-enhanced approach—the key component supporting this claim—has several limitations: i) the data are derived from different cohorts (CSF stream data from healthy controls vs. contrast enhanced data from patients), and ii) the comparison is primarily based on visual inspection of a small number of example images from limited subjects presented. A more quantitative and systematic comparison beyond visual inspection, would substantially strengthen the support for the main conclusion. These limitations should be addressed and also discussed in the discussion section.

Thank you to the authors for addressing the concern regarding partial volume effects. Showing the principal orientation and the non-motion-sensitized images does help alleviate concerns about this potential confound. However, the argument regarding the impact of respiration and cardiac pulsation on high CSF mobility in PVSAS—and the conclusion that the observed higher mobility is not caused by cardiac or respiratory effects but by the presence of a membrane because PVSAS and SAS are affected similarly—is not yet fully convincing (Figure 8).
Or if the key supporting argument is instead the presence of a sharp drop-off of the high mobility from PVSAS to SAS as mentioned in the response and manuscript, please clarify how “sharp” is defined quantitatively, what threshold defines a sharp transition, and what percentage of vessel segments demonstrate this sharp versus non-sharp pattern? This would support the conclusion beyond just reliance on visual inspection of the sharp drop-off and representative images.

(Remarks on code availability)

Reviewer #4

(Remarks to the Author)
I thank the authors for addressing my concerns.

(Remarks on code availability)
I did not a detailed assessment or reproducibilty check but the code seems to be documented well and links work.

Version 2:

Reviewer comments:

Reviewer #1

(Remarks to the Author)
The authors have addressed my prior concerns in this revision.

(Remarks on code availability)

Open Access This Peer Review File is licensed under a Creative Commons Attribution 4.0 International License, which permits use, sharing, adaptation, distribution and reproduction in any medium or format, as long as you give appropriate credit to the original author(s) and the source, provide a link to the Creative Commons license, and indicate if changes were made.
In cases where reviewers are anonymous, credit should be given to 'Anonymous Referee' and the source.
The images or other third party material in this Peer Review File are included in the article's Creative Commons license, unless indicated otherwise in a credit line to the material. If material is not included in the article's Creative Commons license and your intended use is not permitted by statutory regulation or exceeds the permitted use, you will need to obtain permission directly from the copyright holder.
To view a copy of this license, visit https://creativecommons.org/licenses/by/4.0/

RESPONSE TO THE REVIEWERS' COMMENTS

We really appreciate the thoughtful comments of all reviewers, who noted the innovation of the study and the importance of this work in the broader literature. The reviewers raised several issues that needed to be addressed. We have revised the manuscript with the new text additions written in red, and several new figures to address these issues, as discussed in detail below. Changed texted and figures can be found underneath each corresponding question.

Reviewer #1 (Remarks to the Author):

This is an exciting and impactful study that seeks to compare two recent cutting edge neuroimaging data sets to answer important questions concerning the anatomy and function of paravascular CSF transport in the human brain.

To do this, the authors take an innovative approach of visually assessing the spatial patterns of the CSF motility maps generated from the CSF-STREAM technique. By doing so they observe striking similarities to regions of contrast enhancement (from the invasive contrast-enhanced approach using intrathecal delivery of gad) thought to represent para-arterial spaces.

They then characterise spatial patterns of CSF motility in more downstream para-arterial locations (which cannot be easily visualised with the invasive contrast enhanced approach). As such, this novel approach fulfils the title of the work to better characterize PVS spaces in the human brain using a non-invasive approach. It also provides evidence that the CSF-STREAM approach at 7T can map pararterial CSF spaces in healthy volunteers, potentially representing a novel biomarker for early detection of neurologic conditions. Thus, this work would be a very useful addition to the literature.

I have the following critiques:

Major comments:

1. Generally, the clarity of the paper could be significantly improved. Despite being familiar with the two papers whose comparison form the central thesis of the work (references 10 and 11), I had to read the paper multiple times to understand what the hypothesis were that were being tested and what data analysis approach was used to investigate them.

We appreciate the feedback. We have edited the introduction to have a stronger hypothesis:

We changed:

Old text: "Based on the previous intrathecal contrast agent work, we hypothesized that the PVSAS would have higher CSF-mobility around branches of the MCA and ACA (M1-M4, A1-A4)"

to the following:

New text: "Based on the previous intrathecal contrast agent work, we hypothesize that the CSF-mobility maps would share a high level of similarity in their spatial patterns to early timepoint intrathecal contrast-enhanced scans. Because the intrathecal contrast travelled fastest along the PVSAS, we expect PVSAS in healthy controls to exhibit higher CSF-mobility around branches of the MCA and ACA (M1-M4, A1-A4) compared with the adjacent SAS.

To test these hypotheses exploratorily, we first compare CSF-STREAM CSF-mobility maps in healthy controls to intrathecal contrast-enhanced scans from reference patients. Given that post-M1 (defined as M2-M4) arteries showed the highest incidence of PVSAS in the intrathecal scans, we then examine the characteristics of CSF-mobility around the post-M1 arteries. Specifically, we hypothesize that CSF-mobility would be significantly higher within the PVSAS than in the surrounding SAS. We further hypothesize that CSF-mobility principal orientation will be more homogenous within the PVSAS compared to the SAS, reflecting an anatomical constraint, and that transitions at the ICA bifurcation would mark the emergence of distinct PVSAS compartments. Motivated by recent reports of arachnoid cuff granulation (ACE) points[1], we then examine arteries and veins adjacent to the superior sagittal sinus (SSS) for PVSAS CSF-mobility patterns. Finally, we examine possibly drivers of the CSF-mobility by looking at how cardiac and respiration dynamics modulate CSF-mobility within the PVSAS and SAS."

2. Therefore, the authors should clarify what were the precise questions that they were seeking to answer from their analysis and what was the analysis approach that they took to answer these questions. In order to achieve this I recommend having separate sub-headings for each of the questions that match between the aims/ results and methods and figures so that this process is more clear to the reader.

Thank you for the suggestion. We added section headings and sub-sections as follows to match the aims to add more clarity:

CSF-mobility and contrast-enhanced scans show similar PVSAS

CSF-mobility around arteries show distinct PVSAS characteristics

High CSF-mobility PVSAS near the superior sagittal sinus

Cardiac and respiration dynamics modulate the PVSAS

3. This is important, as when you have two large multi-dimensional data sets as is the case here (CSF-STREAM & dynamic contrast enhanced), there are so many possible metrics that you could extract from the data that the analysis approach should carefully consider subjective bias and multiple comparisons etc. Ideally exploratory analysis should be performed, hypotheses generated and then tested on a separate data set. The reader may be sceptical, for example as often just a single intrathecal data set is shown, to what extent subjective bias has come into the choice of data shown and to what extent the analysis is circular if the same data has been used to generate the hypothesis.

We agree that multidimensional datasets can introduce subjective bias and multiple comparisons issues. The primary goal of this manuscript is to characterize PVSAS in healthy

controls using CSF-STREAM and compare CSF-mobility patterns to intrathecal reference patient data to provide context. To address the concern of overinterpretation of a single dataset, we have now included additional PVSAS examples obtained after both CSF-STREAM and intrathecal MRI (Figure 2j; Supplemental Figure 2; Figure 4). Additionally, we originally did exploratory analysis on one dataset and then defined the rest of the PVSAS based on the definitions derived from the original dataset.

Below are added text to address the exploratory analysis, and additional CSF-STREAM and intrathecal contrast agent scans in multiple subjects that have been added to the manuscript:

New text:

“To minimize subjective bias, we established a predefined set of criteria for classification based on an initial reference subject, and these criteria were then applied consistently to all subsequent cases. Specifically, CSF-mobility patterns were categorized according to predefined morphology with quantitative thresholds.”

New figures:

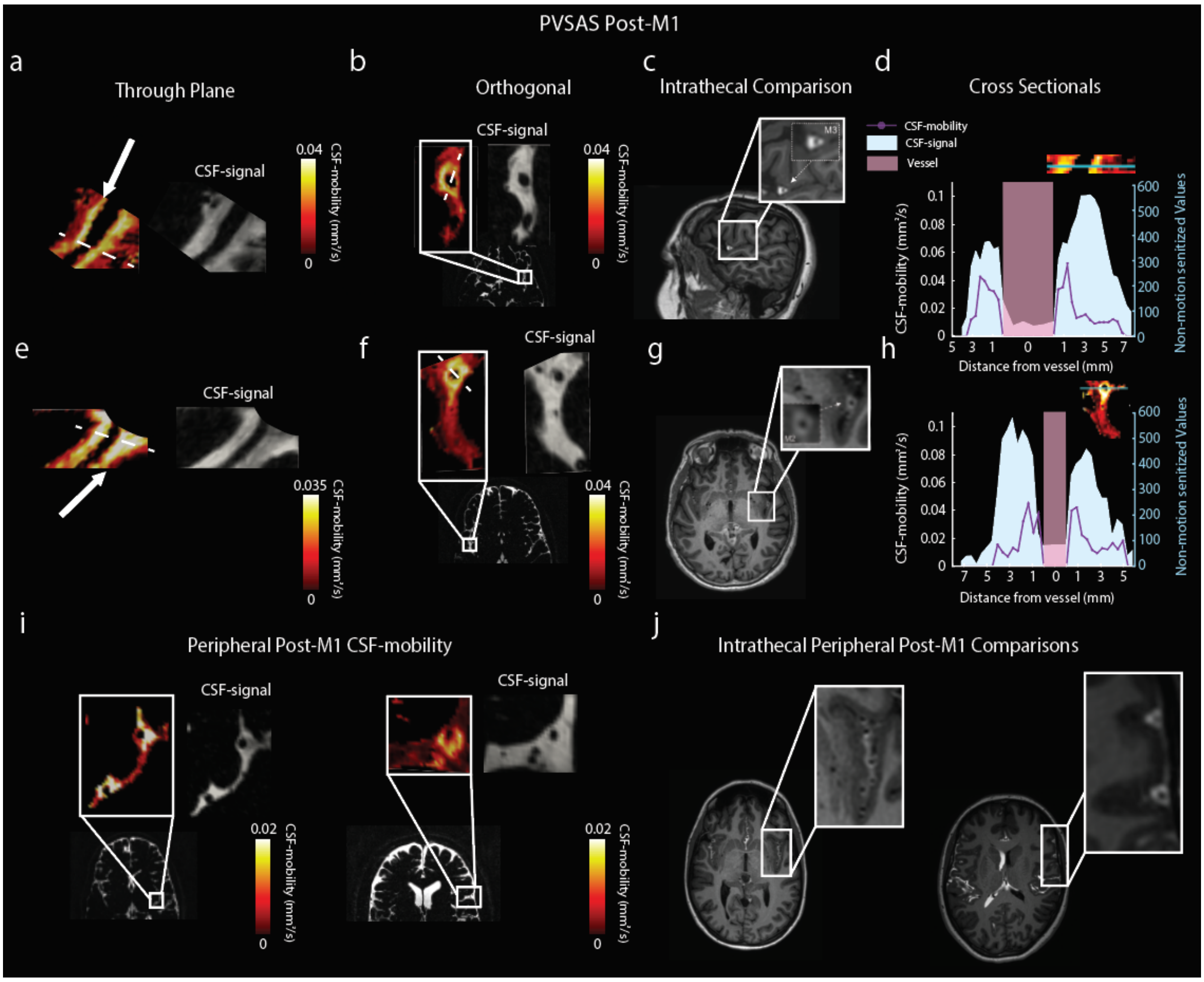

Figure 2 | Perivascular subarachnoid space (PVSAS) in the post-M1 (M2–M4) region.

a/e) *First column* shows through-plane views of orthogonal CSF from the first panel, displaying CSF-mobility. Non-motion-sensitized scans (gray) (referred to as 'CSF-signal') confirm CSF presence in both high- and low-mobility regions. Dotted lines indicate locations of the corresponding orthogonal images in second panel.

b/f) *Second column* shows full-donut PVSAS with high CSF-mobility surrounding post-M1 arteries, with some structural heterogeneity, indicating that the full-donut PVSAS can deviate from perfect circularity (e.g., f), alongside matching non-motion-sensitized scans (gray). White boxes mark regions used for PVSAS extraction; dotted lines indicate locations of the corresponding through-plane images in second panel.

c/g) *Third column* shows post-M1 intrathecal contrast-enhanced PVSAS from patients from similar anatomical locations as in the second column (examples are from Eide & Ringstad, 2024).

d/h) *Fourth column* presents representative cross-sectional plots from similar areas in the first and second columns. The pink bar marks the vessel; the x-axis shows distance from the vessel (mm), with CSF-mobility (purple, left y-axis) and non-motion-sensitized signal (blue, right y-axis). ROI location on the CSF-mobility map is indicated in the top right of the plot.

i) Peripheral CSF-mobility maps with paired non-motion-sensitized scans.

j) Post-M1 intrathecal contrast-enhanced PVSAS acquired from reference patients at anatomical locations matching those in 'i'.

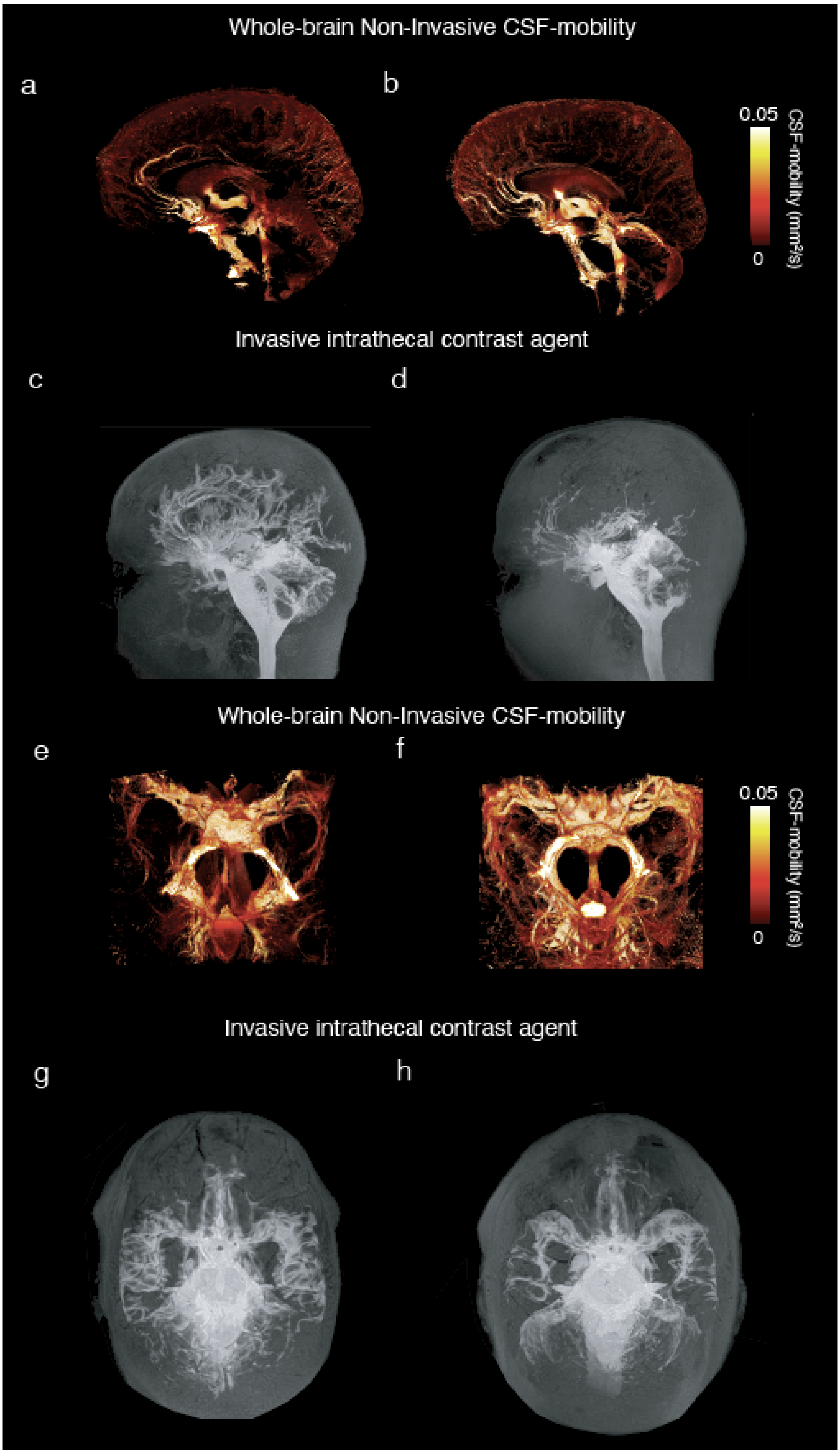

Supplemental Figure 2 | Examples of whole-brain non-invasive CSF-mobility and intrathecal contrast scans.

a/b) Representative sagittal examples of CSF-mobility scans in healthy controls shows perivascular subarachnoid space (PVSAS) surrounding the anterior carotid artery (ACA).

c/d) Sagittal examples of intrathecal contrast scans of reference patients shows contrast in the PVSAS surrounding the ACA.

e/f) Transverse views of a CSF-mobility scans indicate high CSF-mobility PVSAS around the middle cerebral artery (MCA).

g/h) Transverse views of an intrathecal contrast scans of reference patients show contrast filling the PVSAS around the MCA.

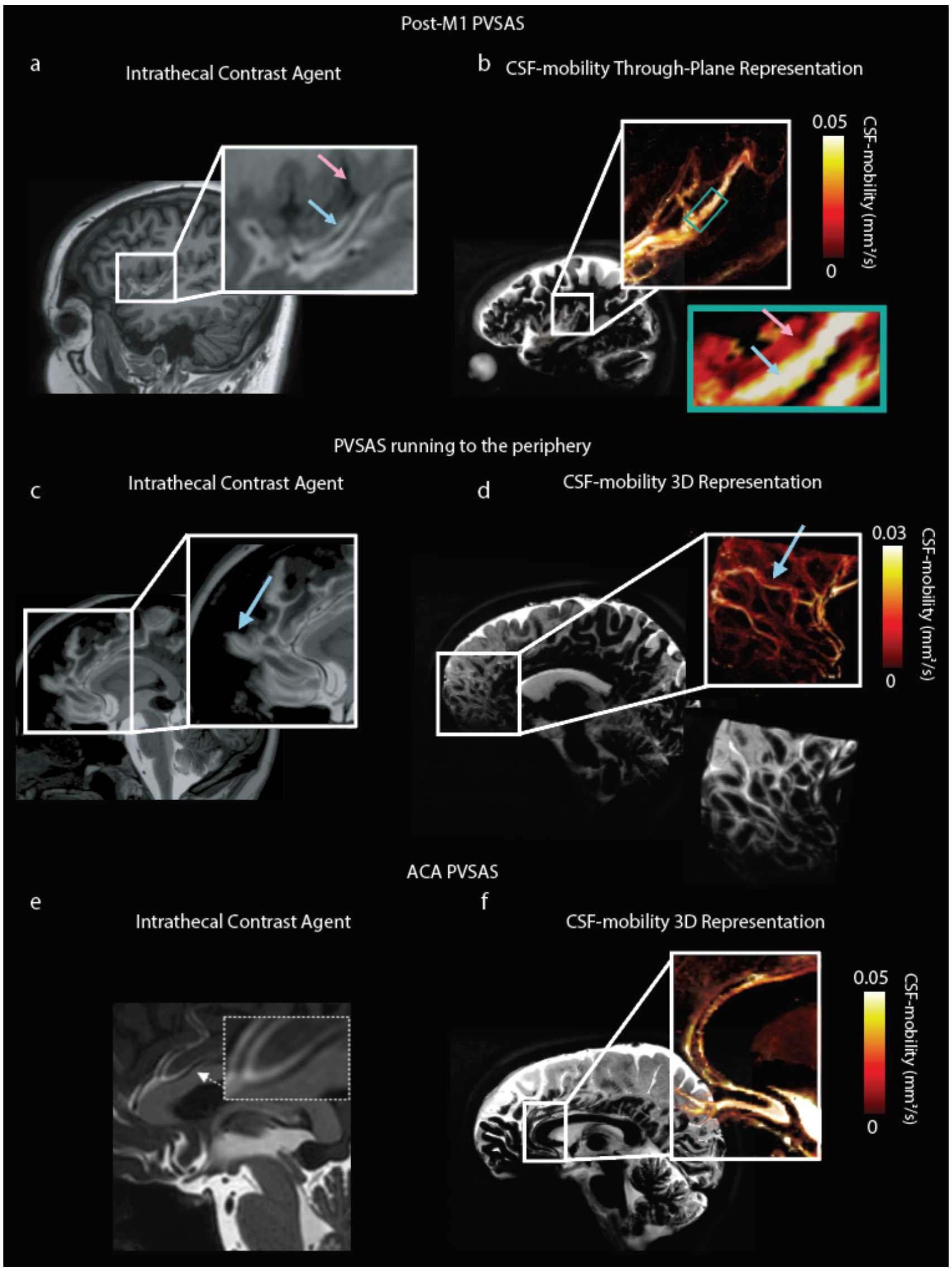

Supplemental Figure 4: Intrathecal and perivascular subarachnoid space (PVSAS) comparisons.

a) Intrathecal contrast agent travelling along the post-M1 shows contrast in the perivascular subarachnoid space (PVSAS) close to the vessel (blue arrow), and no contrast in the subarachnoid space (SAS) (pink arrow).
b) CSF-mobility of the post-M1 PVSAS. Gray is MIP of non-motion sensitized scan, and in the white box is the CSF-mobility, showing MIP of post-M1 PVSAS. Green box shows cross sectional of post-M1 PVSAS show steep drop off between the PVSAS (blue) and the SAS (pink).
c) Intrathecal contrast agent scan shows contrast running to the periphery in the PVSAS (blue arrow).
d) CSF-mobility, showing PVSAS running to the periphery. Gray MIP of non-motion sensitized scan.
e) Intrathecal contrast agent runs along anterior carotid artery (ACA) shows the PVSAS.
f) CSF-mobility 3D-representation of ACA shows limited SAS around the PVSAS.

4. The whole brain comparisons are v interesting and reveal striking similarities. This is shown elegantly in Figure 1 (a,b,d,e). However I am unclear where Figure 1(g,h) fits into this comparison as these are not referenced in the text until much later so it would help clarity if these sections were moved to a later figure. In addition, the whole brain comparison is substantiated by just 1 data set for each modality (and it is not clear how these were chosen). It is fine to have a representative example, but more whole brain comparison data sets should be given in the supplementary material in order to provide more evidence to substantiate the apparent similarity between the whole brain maps.

Thank you for the feedback. We have moved Figure 1(g,h) to a separate figure (Figure 6) for clarity. Additionally, we have added more examples of the CSF-mobility maps and intrathecal brain images to the supplemental materials (Supplemental Figure 2).

Below are changed figures:

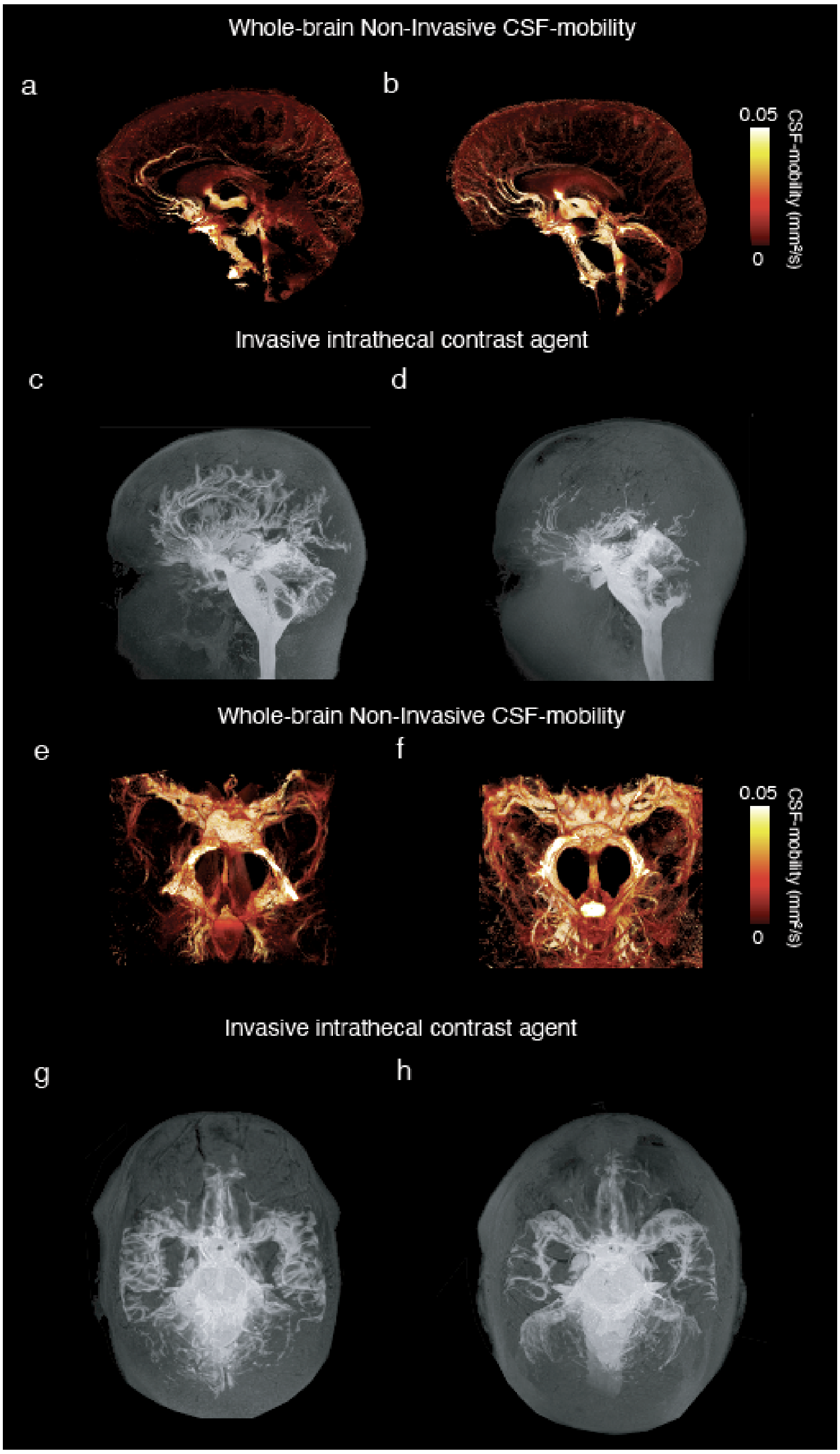

Supplemental Figure 2 | Examples of whole-brain non-invasive CSF-mobility and intrathecal contrast scans.

a/b) Representative sagittal examples of CSF-mobility scans in healthy controls shows perivascular subarachnoid space (PVSAS) surrounding the anterior carotid artery (ACA).

c/d) Sagittal examples of intrathecal contrast scans of reference patients shows contrast in the PVSAS surrounding the ACA.

e/f) Transverse views of a CSF-mobility scans indicate high CSF-mobility PVSAS around the middle cerebral artery (MCA).

g/h) Transverse views of an intrathecal contrast scans of reference patients show contrast filling the PVSAS around the MCA.

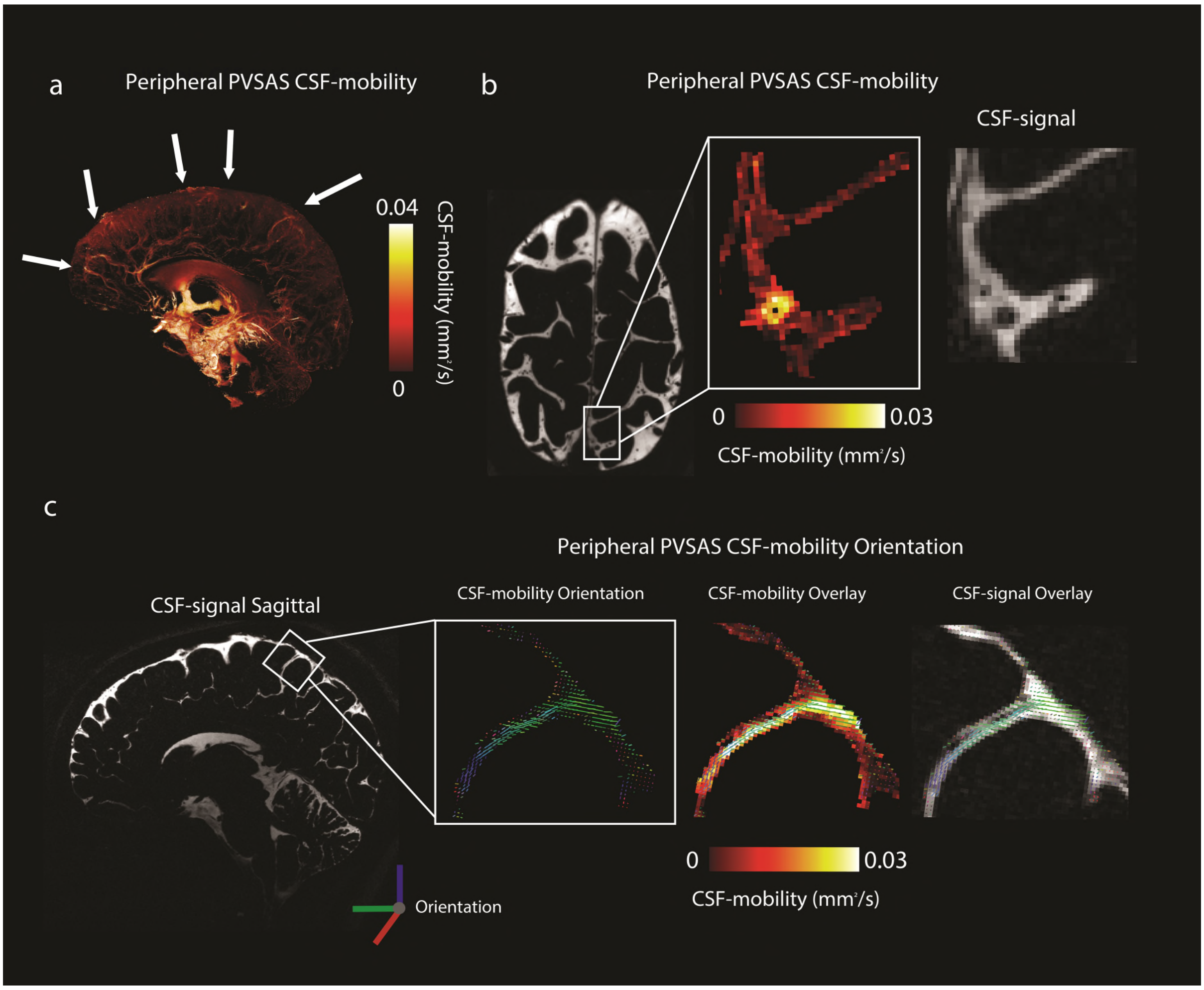

Figure 6 | Perivascular subarachnoid space (PVSAS) near the superior sagittal sinus (SSS).

a) Whole-brain example from a subject exhibiting numerous high CSF-mobility PVSAS near the SSS (white arrows).

b) Peripheral high CSF-mobility PVSAS near the SSS and the non-motion-sensitized scan (gray) confirms the presence of CSF, indicating that reduced mobility is not due to the absence of fluid.

c) Principal orientation of CSF-mobility around peripheral PVSAS. In the peripheral PVSAS, CSF principal orientation travels anterior/posteriorly and then travels around the vessel superior/inferiorly indicated by the green and blue lines. In the SAS, there is lower directional coherence. Lines are overlaid on the CSF-mobility and the non-

motion-sensitized (gray) images. Line colors indicate orientation: red=left-right, green=anterior-posterior, blue=superior-inferior.

5. It is my understanding that a critical component of the work is the comparison of para-arterial spaces captured with the contrast enhanced methods vs the CSF motility maps from CSF stream. I believe that the aim of Figure 2(a-c) and Figure 1(c,f) is to make this comparison and in turn to present evidence that the CSF motility maps from the CSF-stream technique can detect the same para-arterial spaces but non-invasively – this is an important finding. However, these data are not presented very clearly or with enough subjects. It would really help the comparison if the data from both techniques is presented in the same format (ie have a zoomed out version from both methods and a zoomed in image with the same FOV showing the equivalent vessel). Thus a lot more space in the Figures should be dedicated to this important comparison. The through plane images (and cross sectional CSF motility plots) from the CSF stream are interesting and could be included as an additional figure/supplementary but there are not equivalent though plane images in the contrast enhanced data so this complicates the comparison. Similar to my comments to figure 1, again as the evidence is primarily based on visual comparisons with n=1, more examples should be given in the supplementary material to substantiate the authors conclusions.

Based on the reviewer comments we have added additional examples of both the CSF-mobility and intrathecal examples and have presented them with similar formats (Figure 2; Figure 3j,k; Supplemental Figure 2; Supplemental Figure 4). We appreciate the suggestion of removing the through-plane images, but we decided to keep them to show how the PVSAS runs down the vessel to convince the reader that PVSAS is not only observed in single slices.

Here are the figures that have been added/changed:

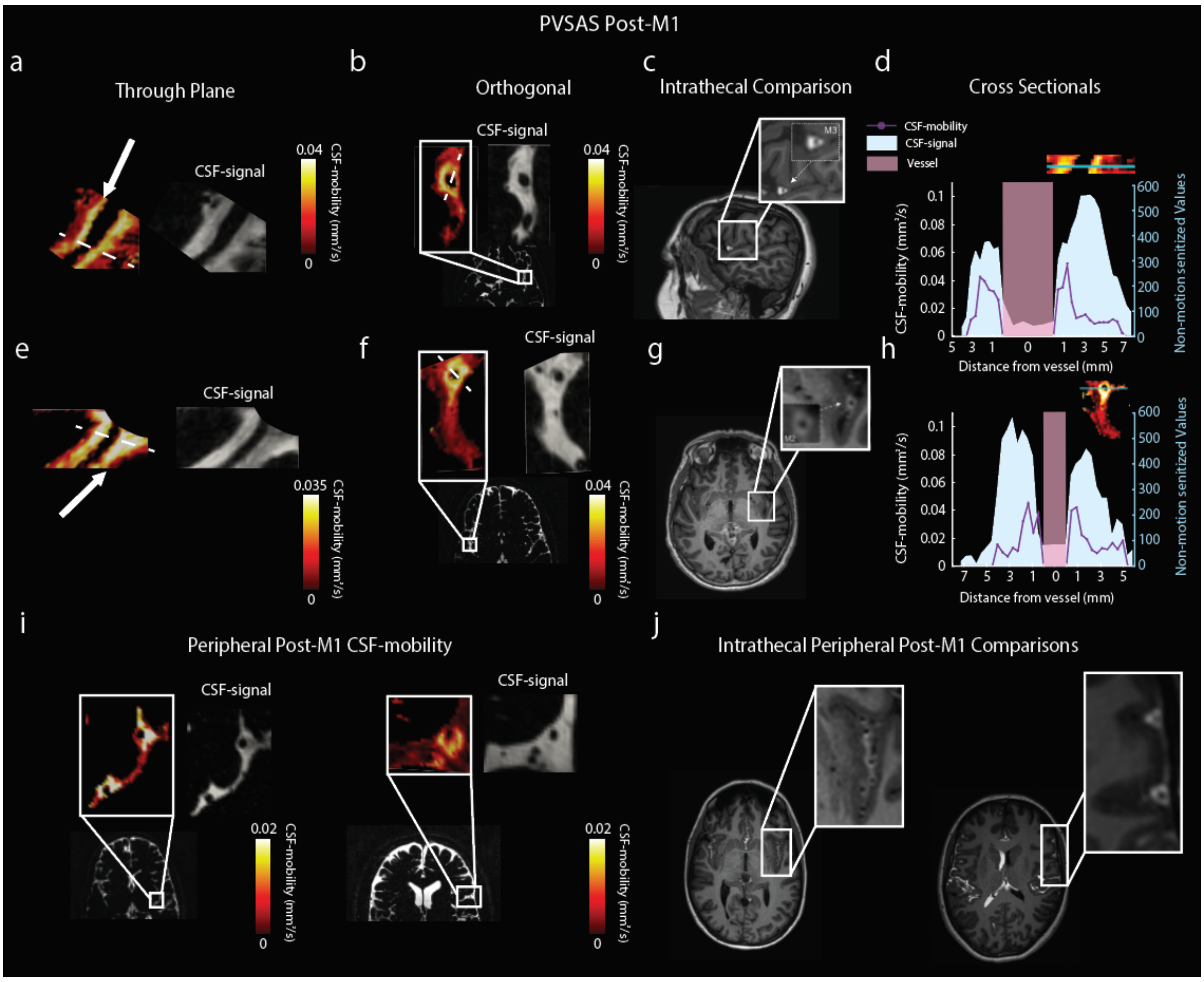

Figure 2 | Perivascular subarachnoid space (PVSAS) in the post-M1 (M2–M4) region.

a/e) *First column* shows through-plane views of orthogonal CSF from the first panel, displaying CSF-mobility. Non-motion-sensitized scans (gray) (referred to as 'CSF-signal') confirm CSF presence in both high- and low-mobility regions. Dotted lines indicate locations of the corresponding orthogonal images in second panel.

b/f) *Second column* shows full-donut PVSAS with high CSF-mobility surrounding post-M1 arteries, with some structural heterogeneity, indicating that the full-donut PVSAS can deviate from perfect circularity (e.g., f), alongside matching non-motion-sensitized scans (gray). White boxes mark regions used for PVSAS extraction; dotted lines indicate locations of the corresponding through-plane images in second panel.

c/g) *Third column* shows post-M1 intrathecal contrast-enhanced PVSAS from patients from similar anatomical locations as in the second column (examples are from Eide & Ringstad, 2024).

d/h) *Fourth column* presents representative cross-sectional plots from similar areas in the first and second columns. The pink bar marks the vessel; the x-axis shows distance from the vessel (mm), with CSF-mobility (purple, left y-axis) and non-motion-sensitized signal (blue, right y-axis). ROI location on the CSF-mobility map is indicated in the top right of the plot.

i) Peripheral CSF-mobility maps with paired non-motion-sensitized scans.

j) Post-M1 intrathecal contrast-enhanced PVSAS acquired from reference patients at anatomical locations matching those in 'i'.

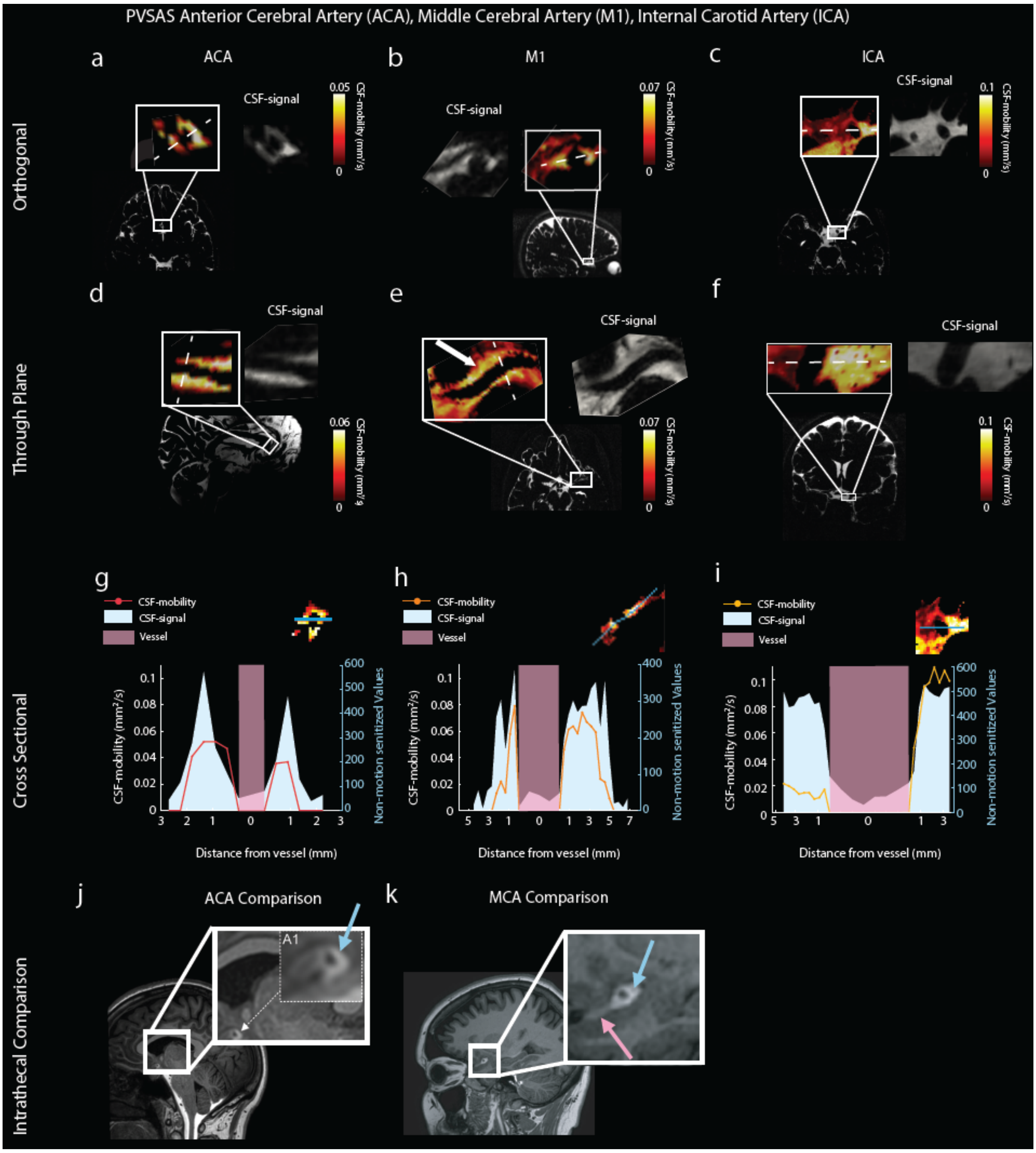

Figure 3 | Perivascular subarachnoid space (PVSAS) across the anterior carotid artery (ACA), M1 and internal carotid artery (ICA).

a) Example of an ACA CSF-mobility PVSAS, displaying tight-CSF in orthogonal image. The non-motion-sensitized scan (gray) (referred to as 'CSF-signal') shows reduction in CSF. The white box marks the extraction site of the PVSAS; the white dotted line indicates the location of the through-plane image seen in 'd'.

b) Example of an M1 CSF-mobility PVSAS showing high CSF-mobility near the artery in a bilateral triangular pattern within a region of lower CSF-mobility. The non-motion-sensitized scan (gray) confirms the presence of CSF in the zoomed-in view, indicating that reduced mobility is not due to absence of CSF. Identical layout to ‘a’.

c) Example of an ICA CSF-mobility two-compartment, showing asymmetric mobility: low CSF-mobility on one side of the artery and high on the other. The non-motion-sensitized scan (gray) confirms CSF presence. White box indicates the extracted region for through-plane image.

d/e/f) Through plane images of ACA, M1, and ICA CSF-mobility. The non-motion-sensitized scan (gray) shows CSF presence. White dotted line shows location of orthogonal cut from ‘a/b/c’.

g/h/i) Representative cross-sectional plots of the PVSAS shown in similar area to orthogonal cut for ACA, M1, and ICA above. Pink bar represents the vessel; the x-axis indicates distance from the vessel (mm), the left y-axis shows CSF-mobility, and the right y-axis shows non-motion-sensitized signal. ROI location is indicated in the corner.

j) ACA (A1) intrathecal contrast scan showing tight contrast-enhancement near the vessel (blue arrow) and can be compared to the ACA PVSAS in ‘a’.

k) MCA intrathecal contrast scan shows high contrast-enhancement next to the vessel (blue arrow), within a lower contrast area (pink arrow), and can be compared to M1 PVSAS in ‘b’.

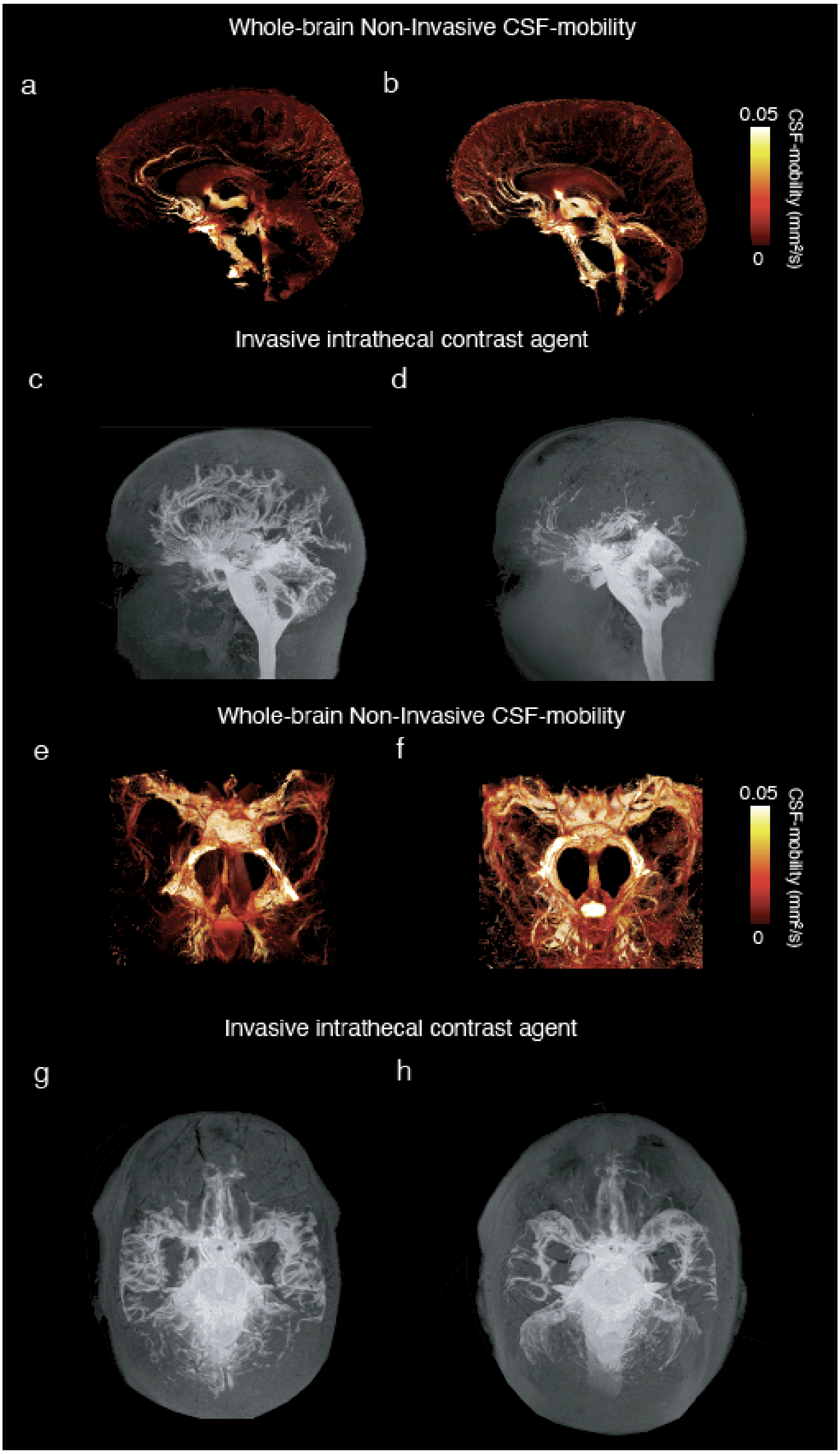

Supplemental Figure 2 | Examples of whole-brain non-invasive CSF-mobility and intrathecal contrast scans.

a/b) Representative sagittal examples of CSF-mobility scans in healthy controls shows perivascular subarachnoid space (PVSAS) surrounding the anterior carotid artery (ACA).

c/d) Sagittal examples of intrathecal contrast scans of reference patients shows contrast in the PVSAS surrounding the ACA.

e/f) Transverse views of a CSF-mobility scans indicate high CSF-mobility PVSAS around the middle cerebral artery (MCA).

g/h) Transverse views of an intrathecal contrast scans of reference patients show contrast filling the PVSAS around the MCA.

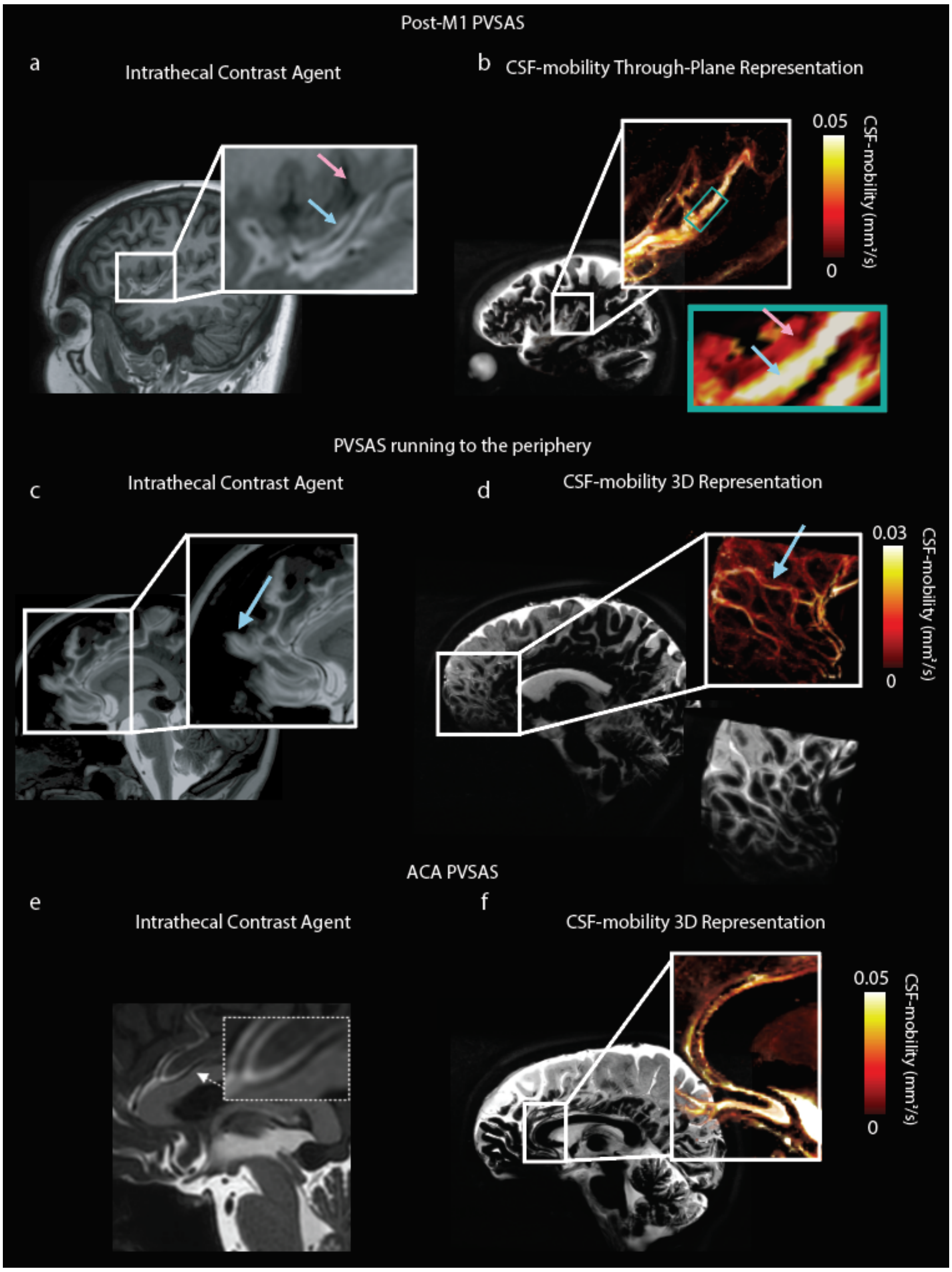

Supplemental Figure 4: Intrathecal and perivascular subarachnoid space (PVSAS) comparisons.

a) Intrathecal contrast agent travelling along the post-M1 shows contrast in the perivascular subarachnoid space (PVSAS) close to the vessel (blue arrow), and no contrast in the subarachnoid space (SAS) (pink arrow).
b) CSF-mobility of the post-M1 PVSAS. Gray is MIP of non-motion sensitized scan, and in the white box is the CSF-mobility, showing MIP of post-M1 PVSAS. Green box shows cross sectional of post-M1 PVSAS show steep drop off between the PVSAS (blue) and the SAS (pink).
c) Intrathecal contrast agent scan shows contrast running to the periphery in the PVSAS (blue arrow).

d) CSF-mobility, showing PVSAS running to the periphery. Gray MIP of non-motion sensitized scan.
e) Intrathecal contrast agent runs along anterior carotid artery (ACA) shows the PVSAS.
f) CSF-mobility 3D-representation of ACA shows limited SAS around the PVSAS.

6. The classification of the CSF-mobility maps into different shapes (ie 'full-donut, tight-CSF, eye-donut, and two-compartment) is very interesting. Can the authors include more of the raw data in the supplementary material so the reader can get a better feel for these data? How was subjective bias minimised in this analysis?

We appreciate the reviewer's suggestion. We have added additional data in the supplemental material (Supplemental Figure 3a-d) of the different shapes. We have also reformatted the descriptions to list-format in the methods for clarity.

We added the following text:

"To minimize subjective bias, we established a predefined set of criteria for classification based on an initial reference subject, and these criteria were then applied consistently to all subsequent cases. Specifically, CSF-mobility patterns were categorized according to predefined morphology with quantitative thresholds.

The categories were defined as follows:

- **Full-donut:** high CSF-mobility around ≥50% of the vessel circumference.
- **Eye-CSF:** high CSF-mobility visible on both lateral sides of the vessel within a lower CSF-mobility area.
- **Tight-CSF:** high CSF-mobility visible around at least 50% of the vessel with less than a two-voxel circumference of low CSF-mobility. Additionally, to be classified as tight-CSF the mobility had to be higher than 0.02 mm^2/s. The lower bound was chosen based on prior reports indicating this value marks the approximate boundary between higher-mobility around the MCA and lower-mobility CSF in the perivascular spaces, and SAS near the sulci[11].
- **Two-Compartment:** high-CSF-mobility with adjacent low-CSF-mobility next to the vessel.
- **No donut:** no marked change in CSF-mobility around the vessel relative to the surrounding SAS, or less than 50% of the vessel circumference.

Quantitative thresholds were applied to further reduce rater bias:

- **High CSF-mobility:** a circumference of ≥2 voxels and ≥20% change relative to SAS.

These definitions were consistently applied to all datasets, and classification was based on the thresholds."

Additional PVSAS figures:

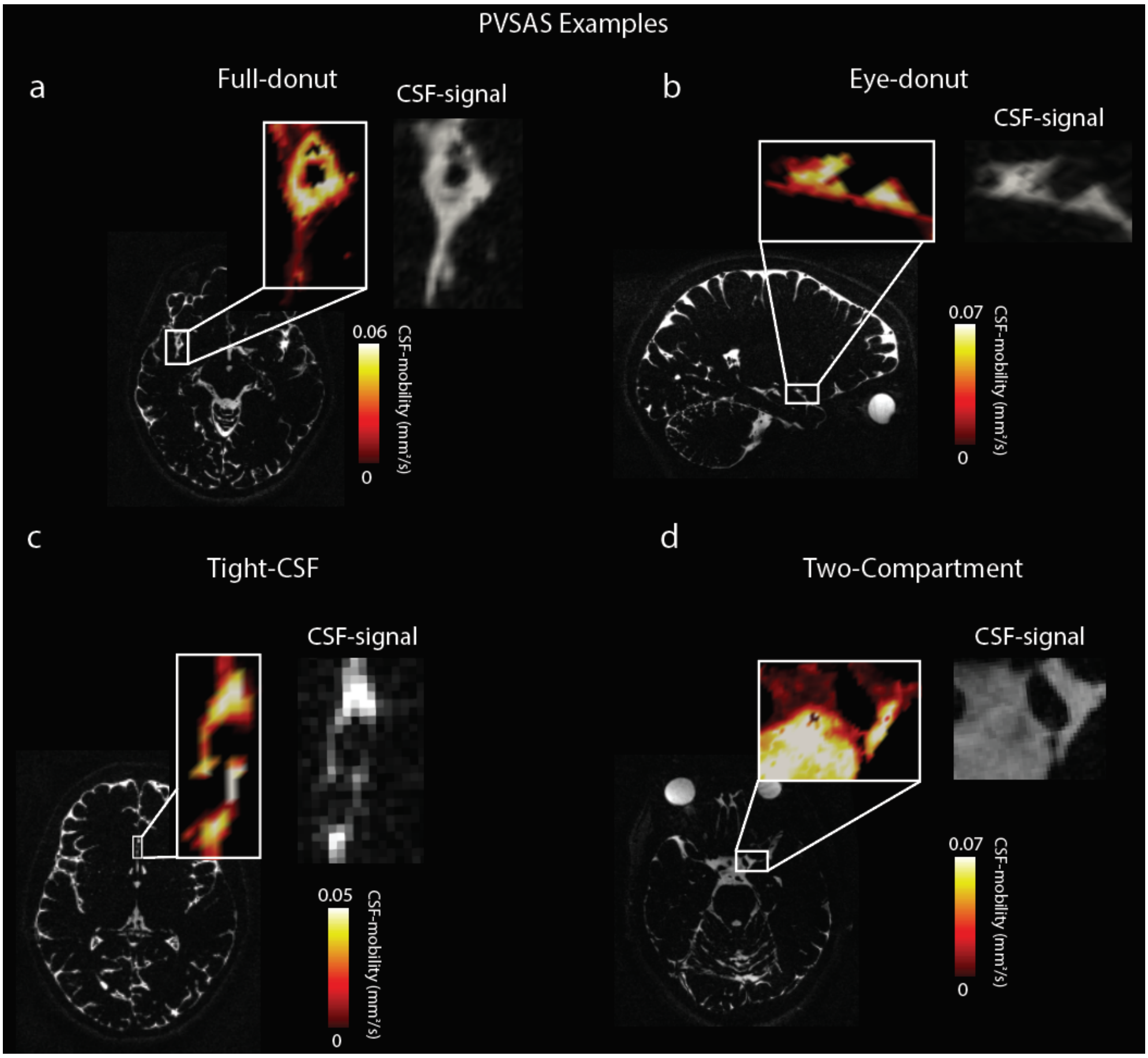

Supplemental Figure 3 | Examples of CSF structure across the anterior carotid artery (ACA), M1 and internal carotid artery (ICA).

a) Full-donut perivascular subarachnoid space (PVSAS).
b) Eye-donut PVSAS.
c) Tight-CSF.
d) Two-compartment.

7. Can the authors comment on whether the FA maps give and insight into pararterial structure and how this compares to the ability of the CSF motility maps to do so.

Thank you for this suggestion, we agree that directionality is an important factor to look at. FA does provide information in this context, but unfortunately FA is not the most stable measure with limited encoding directions[2,3]. Moreover, the important question for this paper is to see whether the direction is consistent within a compartment for efficient transport. Therefore, we have decided to focus in the main document on images showing voxel wise the principal directions, allowing to see whether neighboring voxels show consistent directions. Secondly,

we added visual and quantitative information on FA in the supplemental (Supplemental Figure 5).

We found a statistically significant lower FA in the PVSAS compared to the SAS around the M1 branch (22 segments, 11 subjects, $p=0.0012$, paired t-test, Bonferroni-corrected, 2 tests) and around the post-M1 branch (22 segments, 11 subjects, $p=2.73 \times 10^{-5}$, paired t-test, Bonferroni-corrected, 2 tests) (Supplemental Figure 5c,d). In peripheral PVSAS areas, the FA did not drop relative to the SAS (Supplemental Figure 5).

We found that the principal orientation was consistently more homogeneous in high CSF-mobility PVSAS regions compared to lower CSF-mobility SAS regions (Figure 5; Figure 6).

The new text and figures on the principal orientation are below:

New text:

Post M1 Arteries

> "Principal orientation of the post-M1 CSF-mobility shows more homogenous directionality within the PVSAS compared to the SAS (Figure 5a). This steep drop-off in CSF-mobility from the PVSAS to SAS and increased PVSAS directionality is reflected in the intrathecal contrast agent scans, where the contrast progresses fastest close to the vessel (Figure 2c,g,j)."

ACA

> "The principal orientation of CSF-mobility shows how CSF around the ACA moves along the vessel, i.e. first anterior-posteriorly, and then, as the ACA turns around the genu of the corpus callosum, superior-inferiorly (Figure 5b). Around the ACA, there is often only a single tight-CSF compartment."

M1

> "The principal orientation of the M1 PVSAS CSF-mobility shows that there is homogeneous directionality in the right-left direction, and the SAS has more heterogenous directionality (Figure 5c)."

ICA

> "The ICA principal orientation indicates more directionality in the high CSF-mobility compartment compared to the low CSF-mobility area (Figure 5d)."

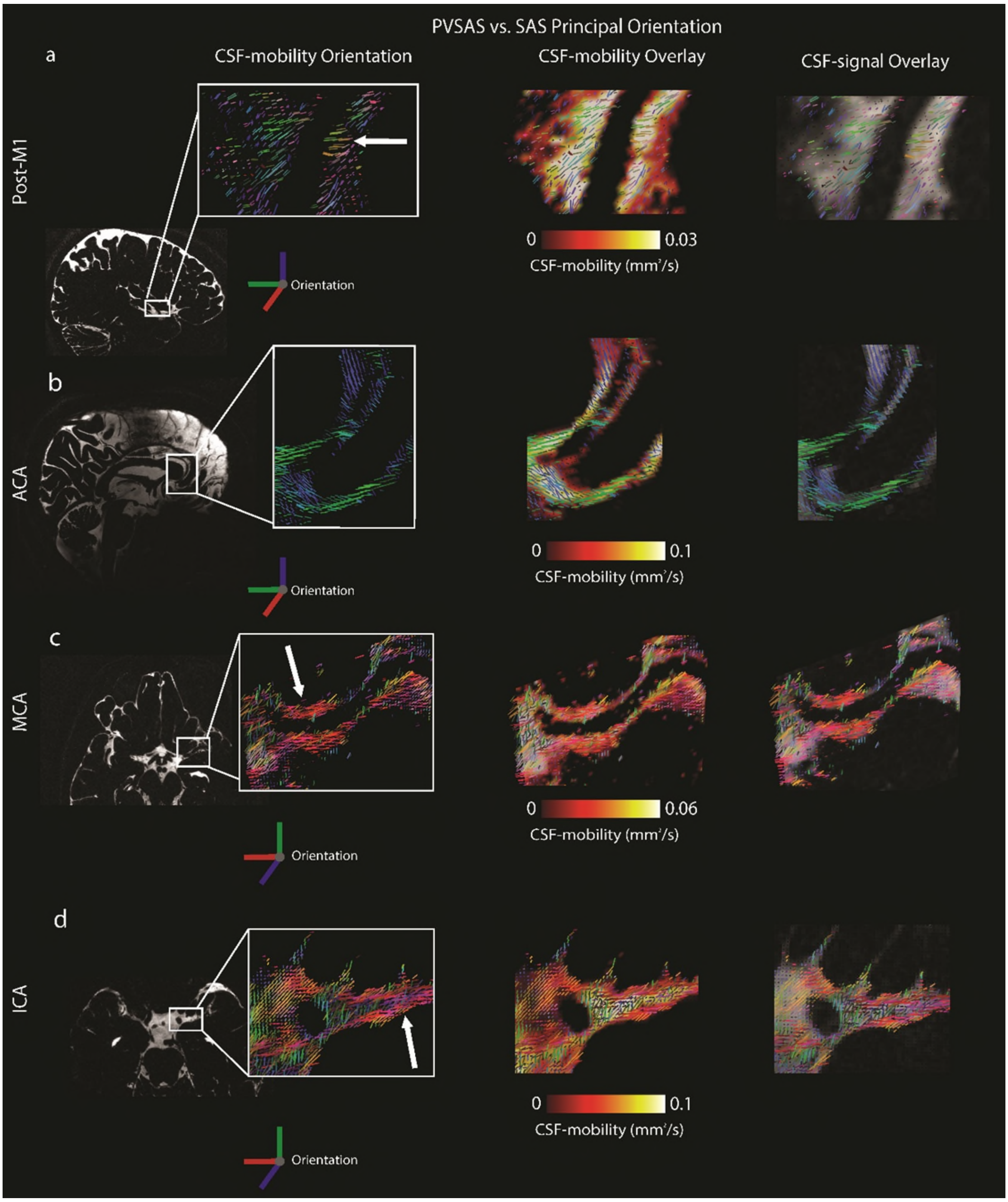

Figure 5 | Principal orientations within the perivascular subarachnoid space (PVSAS).

Principal orientations are overlaid on a through-plane of the PVSAS, shown on both the CSF-mobility and the non-motion-sensitized (gray) images. Line colors indicate orientation: red=left-right, green=anterior-posterior, blue=superior-inferior. White arrows point to PVSAS.

a) In the post-M1 PVSAS, CSF principal orientation shows greater directional coherence, indicated by the blue and green lines. In the SAS, CSF principal orientation shows lower directional coherence, reflected by more heterogeneous lines.

b) In the ACA PVSAS, CSF principal orientation travels anterior-posteriorly and then travels around the vessel superior-inferiorly indicated by the green and blue lines; there is limited SAS present.

c) Around the M1, large red lines within the PVSAS indicate more directionality in the left/right direction. In the SAS near the M1, there is more heterogenous color distribution, meaning less directional coherence.

d) The ICA shows two-compartments in the CSF-mobility, which is reflected with the principal orientation: in the high CSF-mobility region, there is more directional coherence, and lower directional coherence in the lower CSF-mobility region.

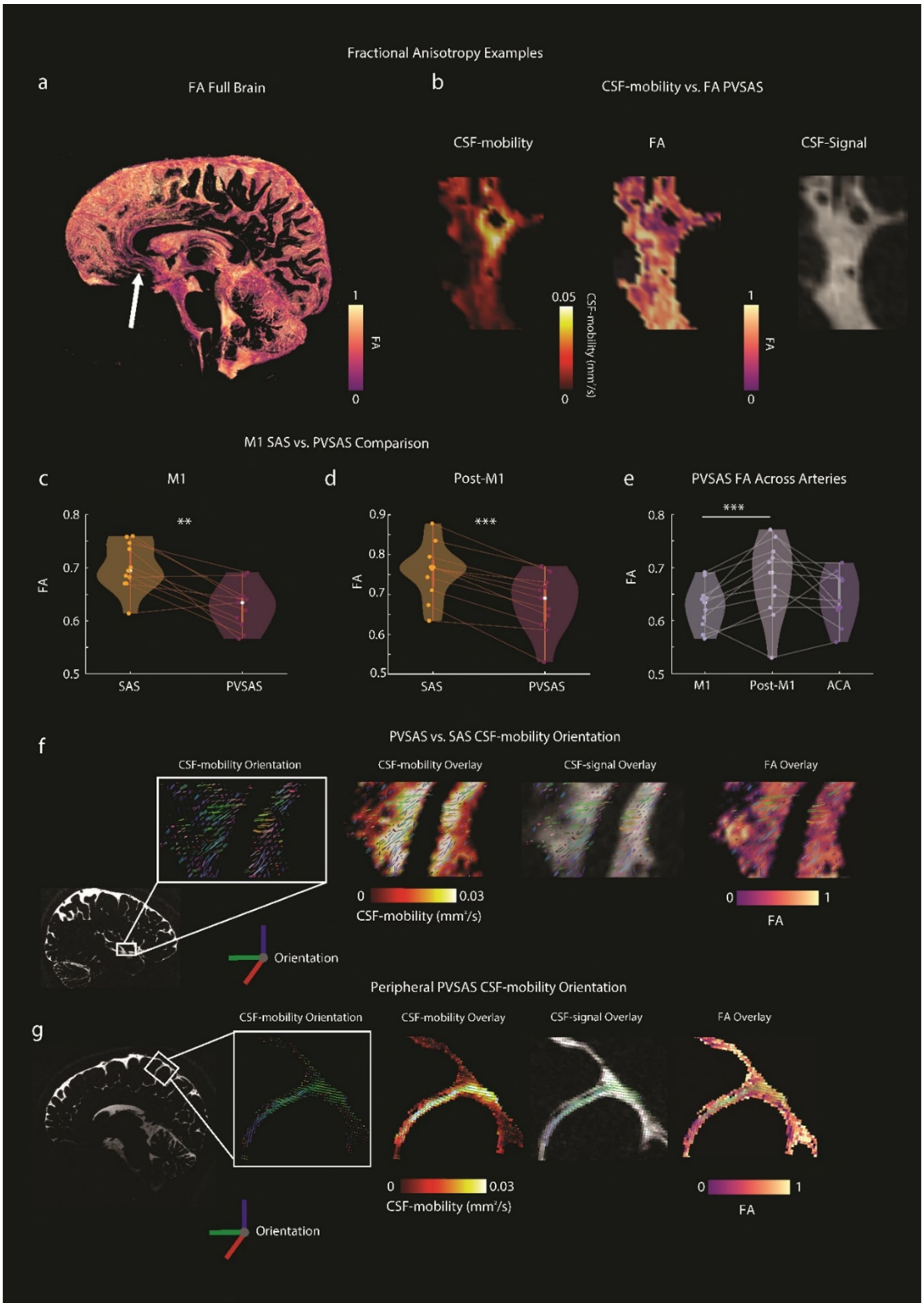

Supplemental Figure 5 | Fractional anisotropy (FA) in perivascular subarachnoid space (PVSAS).

a) Sagittal full brain example of the fractional anisotropy (FA) shows decreased FA around the anterior carotid artery (ACA).

b) Example of post-M1 PVSAS with CSF-mobility and matching FA maps. Decreased FA can be seen within the PVSAS with surrounding higher FA.

c/d) Violin plot comparing SAS vs. PVSAS FA values show statistically significant decrease in the PVSAS in the M1 (n=22 segments, 11 subjects, p=1.2x10^{-3}, paired t-test, Bonferroni-corrected, 2 tests) and post-M1 (n=22 segments, 11 subjects, p=2.73x10^{-5}, paired t-test, Bonferroni-corrected, 2 tests).

e) Mean FA of PVSAS in the M1, post-M1, and ACA, indicate that there is a statistically significant increase in FA in the post-M1 compared to both the M1 and the ACA (n=22 segments, 11 subjects, M1 vs. post-M1 p=6.75x10^{-3}, M1 vs. ACA p=0.37, post-M1 vs. ACA=0.13, paired t-test, Bonferroni-corrected, 3 tests).

f/g) Comparison between principal orientation of CSF-mobility, overlaid on the CSF-mobility, CSF-signal, and FA show that there is inconsistent FA in the PVSAS depending on if the FA is measured around the MCA or ACA (f), and peripheral PVSAS (g). Line colors indicate orientation: red=left-right, green=anterior-posterior, blue=superior-inferior.

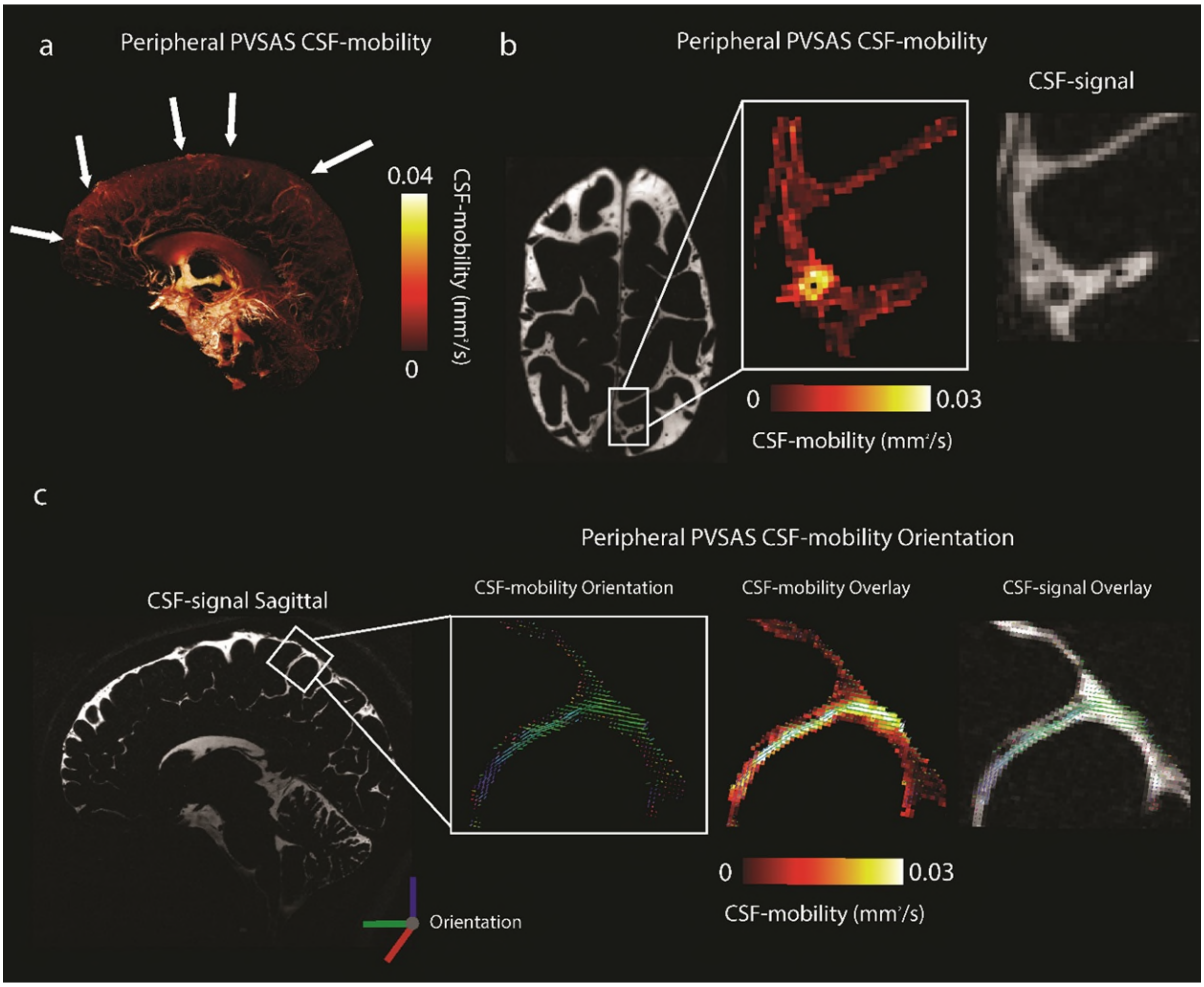

Figure 6 | Perivascular subarachnoid space (PVSAS) near the superior sagittal sinus (SSS).

a) Whole-brain example from a subject exhibiting numerous high CSF-mobility PVSAS near the SSS (white arrows).

b) Peripheral high CSF-mobility PVSAS near the SSS and the non-motion-sensitized scan (gray) confirms the presence of CSF, indicating that reduced mobility is not due to the absence of fluid.

c) Principal orientation of CSF-mobility around peripheral PVSAS. In the peripheral PVSAS, CSF principal orientation travels anterior/posteriorly and then travels around the vessel superior/inferiorly indicated by the green and blue lines. In the SAS, there is lower directional coherence. Lines are overlaid on the CSF-mobility and the non-motion-sensitized (gray) images. Line colors indicate orientation: red=left-right, green=anterior-posterior, blue=superior-inferior.

8. Did the authors also consider applying their PVS classification approach to the CAA patients in reference 11?

We are indeed planning to apply our PVSAS classification approach to patients with CAA as part of a different manuscript (also based on practical considerations, as those data are owned by another group and the current analysis was not part of our original data sharing agreement with that group; a new data-sharing agreement is being prepared, but would take too much time for the current analysis).

Minor comments:

1. I understand that the authors have referenced their previous paper for detailed methods etc but a few more details to explain what their measure of CSF motility is measuring are necessary as the more general reader will be confused that CSF motility is measured in mm2/s.

We have added more information on CSF-mobility to the methods:

Old text: "Detailed methods for image acquisition, reconstruction, and CSF-mobility quantification are available in earlier work".

New text: "Detailed methods for image acquisition, reconstruction, and CSF-mobility quantification are available in earlier work[4]. In short, CSF-STREAM consists in seven subscans: one without motion encoding and six with motion-sensitizing gradients (3.5 mm/s) applied in different orthogonal directions. Importantly, the CSF signal is isolated while suppressing blood and brain tissue contributions thanks to a T2-preparation followed by a long echo-time turbo-spin-echo readout (0.45mm isotropic resolution, FOV = 250 x 250 x 190 mm, TE = 495ms, TR = 3.4s, TSE-factor = 146)."

[...]

"T1 and CSF-STREAM images were registered using Elastix version 4.9.043. From these measurements, CSF-mobility, the principal orientation, and fractional anisotropy (FA) maps

were calculated in MATLAB as the mean eigenvalue of a rank-two positive-definite tensor, similar to diffusion tensor imaging (DTI). CSF-mobility is more sensitive to flow than to diffusion because it is acquired at a very low b-value (motion encoding gradients of 3.5mm/s equivalent to a b-value of 13 s2/mm). The CSF-mobility maps quantify average CSF motion over the acquisition period (38 minutes and 30 seconds)."

2. 'Alternative barriers, such as the subarachnoid lymphatic-like membrane (SLYM) 13,14' – tweak wording to acknowledge current uncertainty in this SLYM (https://www.science.org/doi/10.1126/science.adc8810#elettersSection).

Thank you for the note.

We have changed:

Old text: "Alternative barriers, such as the subarachnoid lymphatic-like membrane (SLYM)[5,6]"

to

New text: "Alternative barriers, such as the debated subarachnoid lymphatic-like membrane (SLYM)[5,6]" to capture the uncertainty.

3. Abstract: Using non-invasive, high-resolution imaging in healthy adults, we observed high CSF-mobility around both proximal and distal cerebral arteries and showing that perivascular subarachnoid spaces (PVSAS) have location-specific structures. – grammar

Thank you for the suggestion.

We have changed the sentence from:

Old text: "Using non-invasive, high-resolution imaging in healthy adults, we observed high CSF-mobility around both proximal and distal cerebral arteries and showing that perivascular subarachnoid spaces (PVSAS) have location-specific structures"

to

New text: "Using non-invasive, high-resolution imaging in healthy adults, we observed high CSF-mobility around both proximal and distal cerebral arteries and show that perivascular subarachnoid spaces (PVSAS) are location-specific.

4. 'These findings suggest that CSF flow may be more spatially distinct than previously thought, providing a foundation for understanding fluid dynamics in health and disease.' – I'm unclear about the rational of this statement as the intrathecal data set which has already been published has already demonstrated that CSF flow is spatially distinct around the paraarterial space vs the subarachnoid space.

Thank you for allowing us to clarify. While previous intrathecal datasets have indeed demonstrated that CSF flow is spatially distinct between the para-arterial space and the subarachnoid space, one of the novelties of the CSF-STREAM dataset is its much higher spatial and temporal resolution. This higher resolution allows us to visualize regions that were previously difficult or impossible to assess. For example:

- Around the M1, the intrathecal contrast agent moves too quickly to reliably capture the detailed shape of the PVSAS, whereas CSF-STREAM provides sufficient temporal and spatial resolution to do so.
- The peripheral PVSAS near the superior sagittal sinus have not been consistently visualized in intrathecal imaging, likely due to the longer time required for the tracer to reach these areas.
- We can now parse the PVSAS into four distinct categories; eye-donut, full-donut, tight-CSF, and two-compartment.

Thus, CSF-STREAM provides new insights into CSF dynamics that complement and extend prior intrathecal findings in patients. Moreover, the fact that CSF-STREAM is completely non-invasive opens many more application opportunities.

5. 'we observed high CSF-mobility around both proximal and distal cerebral arteries and showing that perivascular subarachnoid spaces (PVSAS) have location-specific structures' the data is measures of PVS motility and thus you are not actually measuring structure. Change the wording to clarify this distinction between what you are actually measuring and what you are inferring.

Thank you for the suggestion. We have changed this sentence from:

Old text: "We observed high CSF-mobility around both proximal and distal cerebral arteries and showing that perivascular subarachnoid spaces (PVSAS) have location-specific structures"

to

New text: "We observed high CSF-mobility around both proximal and distal cerebral arteries, indicating that perivascular subarachnoid spaces (PVSAS) may have location-specific characteristics."

6. Intro: 'Based on the previous intrathecal contrast agent work, we hypothesized 86 that the PVSAS would have higher CSF-mobility around branches of the MCA and ACA (M1-M4, A1- 87 A4)' – hypothesis is incomplete – higher then what? What are you comparing to?

We have changed this line from:

Old text: "Based on the previous intrathecal contrast agent work, we hypothesized that the PVSAS would have higher CSF-mobility around branches of the MCA and ACA (M1-M4, A1- 87 A4)"

to

New text: “Based on the previous intrathecal contrast agent work, we hypothesize that the CSF-mobility maps would share a high level of similarity in their spatial patterns to early timepoint intrathecal contrast-enhanced scans. Because the intrathecal contrast travelled fastest along the PVSAS, we expect PVSAS in healthy controls to exhibit higher CSF-mobility around branches of the MCA and ACA (M1-M4, A1-A4) compared with the adjacent SAS.

To test these hypotheses exploratorily, we first compare CSF-STREAM CSF-mobility maps in healthy controls to intrathecal contrast-enhanced scans from reference patients. Given that post-M1 (defined as M2-M4) arteries showed the highest incidence of PVSAS in the intrathecal scans, we then examine the characteristics of CSF-mobility around the post-M1 arteries. Specifically, we hypothesize that CSF-mobility would be significantly higher within the PVSAS than in the surrounding SAS. We further hypothesize that CSF-mobility principal orientation will be more homogenous within the PVSAS compared to the SAS, reflecting an anatomical constraint, and that transitions at the ICA bifurcation would mark the emergence of distinct PVSAS compartments. Motivated by recent reports of arachnoid cuff granulation (ACE) points[1], we then examine arteries and veins adjacent to the superior sagittal sinus (SSS) for PVSAS CSF-mobility patterns. Finally, we examine possibly drivers of the CSF-mobility by looking at how cardiac and respiration dynamics modulate CSF-mobility within the PVSAS and SAS.”

7. Intro: ‘However, recent research challenges the traditional view of the subarachnoid space (SAS), which harbors the CSF, as a single continuous space’ need a reference to support this statement.

Thank you – we have added a reference to support “recent research challenges the traditional view of the subarachnoid space (SAS)[7]” and two references to support “CSF, as a single continuous space[8,9]”:

8. ‘However, these observations were made in patients undergoing intrathecal injections as part of their diagnostic work-up, i.e. with suspicion of a CSF disorder’ -specify that you mean injection of contrast agent.

Thank you for the suggestion.

We have changed the sentence from:

Old text: “However, these observations were made in patients undergoing intrathecal injections as part of their diagnostic work-up, i.e. with suspicion of a CSF disorder”

to

New text: “However, these observations were made in patients undergoing intrathecal contrast agent injections as part of their diagnostic work-up, i.e. with suspicion of a CSF disorder.”

9. It may be worth mentioning in the introduction that a key element of the previous study (reference 10) was that the signal in the para-arterial space correlated to tissue delivery.

'Intrathecal contrast agent-based detection of PVSAS is very time-sensitive: if the scan is performed too early, the contrast agent has not yet arrived; if scanned too late, it has already spread throughout the entire SAS.'

'Intrathecal contrast agent-based detection of PVSAS is very time-sensitive: if the scan is performed too early, the contrast agent has not yet arrived; if scanned too late, it has already spread throughout the entire SAS.' Slightly disingenuous/confusing statement as this is the idea behind the method – it is a dynamic scan and you can get useful information by analysis the time-dependence of the data. I would rephrase this.

We have added additional clarification.

We have changed:

Old text: "Intrathecal contrast agent-based detection of PVSAS is very time-sensitive: if the scan is performed too early, the contrast agent has not yet arrived; if scanned too late, it has already spread throughout the entire SAS"

to

New text: "Intrathecal contrast agent-based detection of PVSAS is very time-sensitive: if a measurement is performed too early, the contrast agent has not yet arrived; if measured too late, it has already spread throughout the entire SAS. While this approach is useful for tracking overall contrast distribution to the brain tissue, it will only provide snapshots of this dynamic process in CSF."

10. 'The presence of distinct, high-mobility perivascular regions in the absence of pathology raises important questions about their role in circulating CSF on the driving forces of the water movement in this compartment as well as throughout the complete brain.' -grammar.

Thank you for the note.

We have changed from sentence from:

Old text: "The presence of distinct, high-mobility perivascular regions in the absence of pathology raises important questions about their role in circulating CSF on the driving forces of the water movement in this compartment as well as throughout the complete brain"

to

New text: "The presence of distinct, high-mobility perivascular regions in the absence of pathology raises important questions: The presence of distinct, high-mobility PVSAS in the absence of pathology raises important questions: how do these compartments circulate CSF, and do they exhibit unidirectional CSF movement?"

Reviewer #2 (Remarks to the Author):

1. Recent work has suggested that CSF flow is not homogeneous within the SAS, but may

instead follow specialized perivascular subarachnoid space (PVSAS) pathways. Building on intrathecal contrast studies, the present work employed CSF-STREAM, a non-invasive, high-resolution MRI technique that isolates CSF signal and maps CSF-mobility, to determine whether PVSAS can be visualized in healthy individuals. In 11 participants, CSF-mobility maps revealed consistent high-mobility regions adjacent to major cerebral arteries, particularly along the MCA (M1–M4) and ACA (A1–A4), closely paralleling patterns reported in intrathecal imaging. Cross-sectional analyses demonstrated "donut-like" perivascular profiles, with significantly elevated CSF-mobility in the PVSAS relative to surrounding SAS. Distinct morphological patterns ("full-donut," "tight-CSF," "eye-donut," and "two-compartment") were classified across arterial segments, with the full-donut configuration most prominent in distal MCA branches. Additional observations near the ICA bifurcation and superior sagittal sinus suggested further heterogeneity of perivascular compartments. These findings demonstrate that CSF-STREAM can non-invasively detect and characterize PVSAS in vivo, providing evidence for anatomically distinct, high-mobility CSF pathways in the healthy human brain. This study is novel and contributes meaningfully to our understanding. By using a non-invasive, tracer-free MRI approach (CSF-STREAM), it demonstrates for the first time that PVSAS can be visualized and quantified in healthy individuals, not just in patients undergoing intrathecal contrast studies. The identification of distinct morphological patterns of CSF-mobility around major cerebral arteries provides new evidence that CSF transport is regionally organized rather than uniform. This advances the concept that perivascular compartments are functional conduits for CSF dynamics, with implications for brain clearance and immune surveillance. Collectively, the findings broaden our mechanistic framework for how CSF flows through the human brain and open opportunities for studying its role in aging and disease. I have no additional comments.

Thank you for your comments.

Reviewer #2 (Remarks on code availability):

2. the GitHub repository cited at the bottom of the first page was not available to view. there was no repository in this account named as "csf_donuts".

Thank you for pointing out the lack of accessibility to the GitHub repository. We have made the repository public:

https://github.com/ninafultz/csf_donuts

Reviewer #3 (Remarks to the Author):

This study examines CSF-mobility within the perivascular subarachnoid space (PVSAS) of the human brain, reporting that mobility is higher around major arteries. While the findings are consistent with prior knowledge and physiologically plausible, the analyses remain limited and overly simplified. As such, they primarily confirm known phenomena without adding much new insights into CSF circulation or physiology. The lack of novel mechanistic understanding and insufficient depth of analysis constrain the overall significance and impact of the work. Major comments are outlined below:

1. It is unclear whether CSF-mobility measures provide any information about the direction of CSF movement. The observed higher mobility may simply reflect signal fluctuations caused by arterial size changes, rather than genuine CSF/glymphatic flow. Without direct evidence of directional CSF flow along arteries, the current results risk being dismissed as partial volume artifacts or pulsation-induced noise rather than meaningful physiological transport.

We appreciate the feedback and the ability to provide clarification. CSF-STREAM provides information about the principal orientation of the CSF-mobility, which we added to the manuscript (Figures 5, Figure 6c): we found that in the PVSAS the CSF was moving along the arteries, whereas in the SAS the orientation of the CSF-mobility was more random.

We found that the principal orientation was consistently more homogeneous in high CSF-mobility PVSAS regions compared to lower CSF-mobility SAS regions (Figure 5; Figure 6).

We are confident that our findings are not influenced by partial volume effects with e.g. the vessel wall or brain tissue. This is because CSF-mobility was specifically measured by isolating the CSF signal from blood and tissue using T_2-preparation and a long echo-time (500 ms), which effectively suppresses blood and tissue components. To further confirm this, we measured the signal intensity in the PVSAS and SAS in non-motion-sensitized scans (Figure 4d,e). If the CSF-mobility changes observed in SAS vs PVSAS were due to partial volume artefacts or pulsation-induced noise, signal intensity differences between the SAS and PVSAS would be expected. Importantly, we found no change in signal intensity in the SAS vs PVSAS, demonstrating that the observed changes in CSF-mobility were not due to differences in CSF presence within both the PVSAS around the M1 (Figure 4d; paired t-test, p=0.32) and the PVSAS around the post-M1 (Figure 4e; paired t-test, p=0.32). We could not measure next to the PVSAS of the ACA because of the lack of SAS.

Additionally, partial volume effects due to vessel pulsation would be in the sub-voxel order (~0.2mm change in diameter), which cannot explain why we see high CSF-mobility in more than one voxel (with each voxel being 0.45mm isotropic). This calculation is based on a mean M1 arterial diameter of 2.55 mm[10] and average distensions from diastole to systole of 2.58%[11], which would lead to a diameter increase of approximately 0.07 mm, well below the 0.45 mm voxel used to image the CSF-mobility.

We acknowledge that attenuation of CSF-STREAM could be due to multiple different flow scenarios, including bulk flow, parabolic flow, oscillatory flow, random motion, or a combination of these flow factors[4]. Additionally, CSF is known to be driven by cardiac, respiration, and vasomotor activity which has been shown on global scale[12,13]. Because of this, we examined both CSF-mobility dynamics with retrospective cardiac and respiration binning in both the SAS and the PVSAS (Figure 8a-b) and we show that relative mobility-fluctuations within the SAS and the PVSAS are comparable, both being equally modulated by the cardiac and respiratory cycles. As the CSF-mobility is higher in PVSAS, the equal relative fluctuations translate still into larger excursions. The origin of the higher CSF-mobility might be more easily explained by the presence of a membrane, although this has not yet been proven to exist.

The sharp drop-off that can be seen in the CSF-mobility (e.g. Figure 2) is an indication that there may be a membrane present. If there was no membrane, and if the observed higher CSF-

mobility was only due to cardiac/respiration pulsations, then a much more gradual drop-off in mobility would be expected as one stepped away from the vessel. Additionally, the intrathecal work that has been done[7] shows that the contrast stays very close to vessel and then diffuses, also pointing to the presence of a membrane.

Below are new text and figures to address the above concerns:

New text to address principal orientation:

Post M1 Arteries

> "Principal orientation of the post-M1 CSF-mobility shows more homogenous directionality within the PVSAS compared to the SAS (Figure 5a). This steep drop-off in CSF-mobility from the PVSAS to SAS and increased PVSAS directionality is reflected in the intrathecal contrast agent scans, where the contrast progresses fastest close to the vessel (Figure 2c,g,j)."

ACA

> "The principal orientation of CSF-mobility shows how CSF around the ACA moves along the vessel, i.e. first anterior-posteriorly, and then, as the ACA turns around the genu of the corpus callosum, superior-inferiorly (Figure 5b). Around the ACA, there is often only a single tight-CSF compartment."

M1

> "The principal orientation of the M1 PVSAS CSF-mobility shows that there is homogeneous directionality in the right-left direction, and the SAS has more heterogenous directionality (Figure 5c)."

ICA

> "The ICA principal orientation indicates more directionality in the high CSF-mobility compartment compared to the low CSF-mobility area (Figure 5d)."

New text to address partial volume effects:

"CSF-mobility is specifically measured by isolating the CSF signal from blood and tissue using T2-preparation and a long echo-time (500 ms), thereby limiting partial volume effects. To further confirm this, we measured the signal intensity in the PVSAS and SAS in non-motion-sensitized scans (Figure 4d,e). If the CSF-mobility changes observed in SAS vs PVSAS were due to partial volume artifacts or pulsation-induced noise, signal intensity differences between the SAS and PVSAS would be expected. Importantly, we found no change in signal intensity in the SAS vs PVSAS, demonstrating that the observed changes in CSF-mobility were not due to differences in CSF presence."

New text to address impact of cardiac and respiration:

"CSF is thought to be driven by cardiac, respiration, and vasomotor activity[12,13]. Because of this, we examined whether the PVSAS would have distinct cardiac and respiration dynamics in comparison with the SAS. We found that overall, CSF-mobility PVSAS and SAS are modulated most with the cardiac cycle (Figure 8a) and are minorly modulated with respiration (Figure 8b) in the M1, post-M1, and ACA PVSAS. In the PVSAS, the absolute cardiac and respiratory CSF-mobility pulsatility is higher than the SAS. However, the relative mobility-fluctuations within the SAS and the PVSAS are comparable, both being equally modulated by the cardiac and respiratory cycles."

[…]

"Finally, CSF-mobility is similarly modulated in PVSAS and SAS by cardiac and respiration dynamics. The cardiac cycle was found to have more influence than the respiratory cycle, in line with previous work[4]. As the CSF-mobility is higher in PVSAS, the equal relative fluctuations translate still into larger excursions. The origin of the higher CSF-mobility might be more easily explained by the presence of a membrane."

New/changed figures to address concerns above:

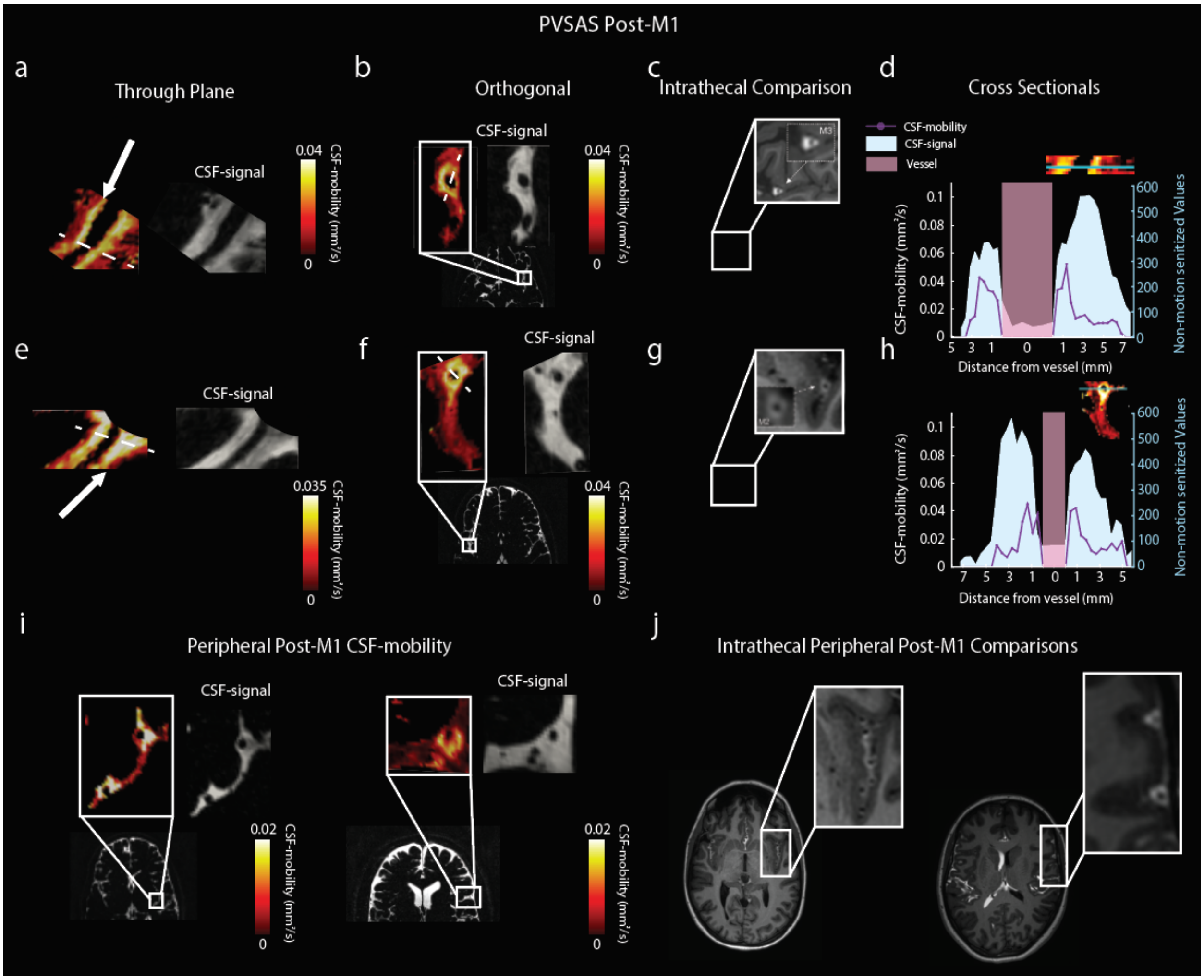

Figure 2 | Perivascular subarachnoid space (PVSAS) in the post-M1 (M2–M4) region.

a/e) *First column* shows through-plane views of orthogonal CSF from the first panel, displaying CSF-mobility. Non-motion-sensitized scans (gray) (referred to as 'CSF-signal') confirm CSF presence in both high- and low-mobility regions. Dotted lines indicate locations of the corresponding orthogonal images in second panel.

b/f) *Second column* shows full-donut PVSAS with high CSF-mobility surrounding post-M1 arteries, with some structural heterogeneity, indicating that the full-donut PVSAS can deviate from perfect circularity (e.g., f), alongside matching non-motion-sensitized scans (gray). White boxes mark regions used for PVSAS extraction; dotted lines indicate locations of the corresponding through-plane images in second panel.

c/g) *Third column* shows post-M1 intrathecal contrast-enhanced PVSAS from patients from similar anatomical locations as in the second column (examples are from Eide & Ringstad, 2024).

d/h) *Fourth column* presents representative cross-sectional plots from similar areas in the first and second columns. The pink bar marks the vessel; the x-axis shows distance from the vessel (mm), with CSF-mobility (purple, left y-axis) and non-motion-sensitized signal (blue, right y-axis). ROI location on the CSF-mobility map is indicated in the top right of the plot.

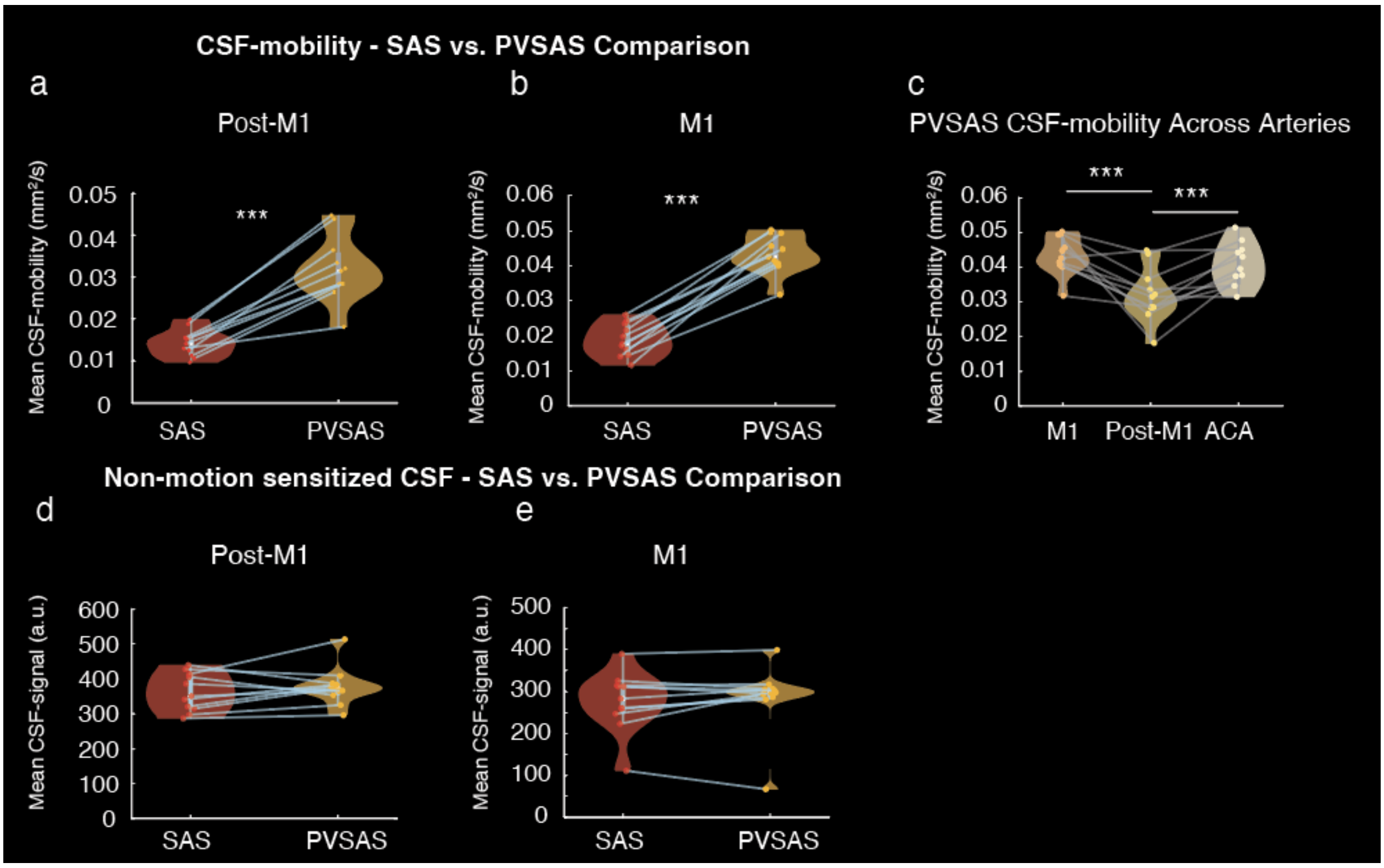

Figure 4 | Elevated CSF-mobility in the perivascular subarachnoid space (PVSAS) relative to the subarachnoid space (SAS).

a) Violin plot of post-M1 mean CSF-mobility in two subsections of the SAS and PVSAS (n=22 segments, 11 subjects, $p=2.0\times10^{-6}$, paired t-test, Bonferroni-corrected, 2 tests).

b) Violin plot of M1 mean CSF-mobility in two subsections of PVSAS and SAS (22 segments, 11 subjects, $p=6.5 \times 10^{-8}$, paired t-test, Bonferroni-corrected, 2 tests).

c) PVSAS CSF-mobility values across M1, post-M1, and ACA, shows significantly more CSF-mobility in the M1 and ACA PVSAS than the post-M1 PVSAS (11 subjects, M1 vs. post-M1: $p=1.49 \times 10^{-3}$; M1 vs. ACA: $p=0.192$; post-M1 vs. ACA: $p = 1.24 \times 10^{-3}$, paired t-test, Bonferroni-corrected, 3 tests).

d/e) SAS and PVSAS ROIs of the post-M1 and M1 on non-motion-sensitized images show similar CSF signal intensity, indicating that differences in CSF-mobility between SAS and PVSAS is not due to change in CSF presence (11 subjects, post-M1 p=0.32, M1 p=0.32, paired t-test, Bonferroni-corrected).

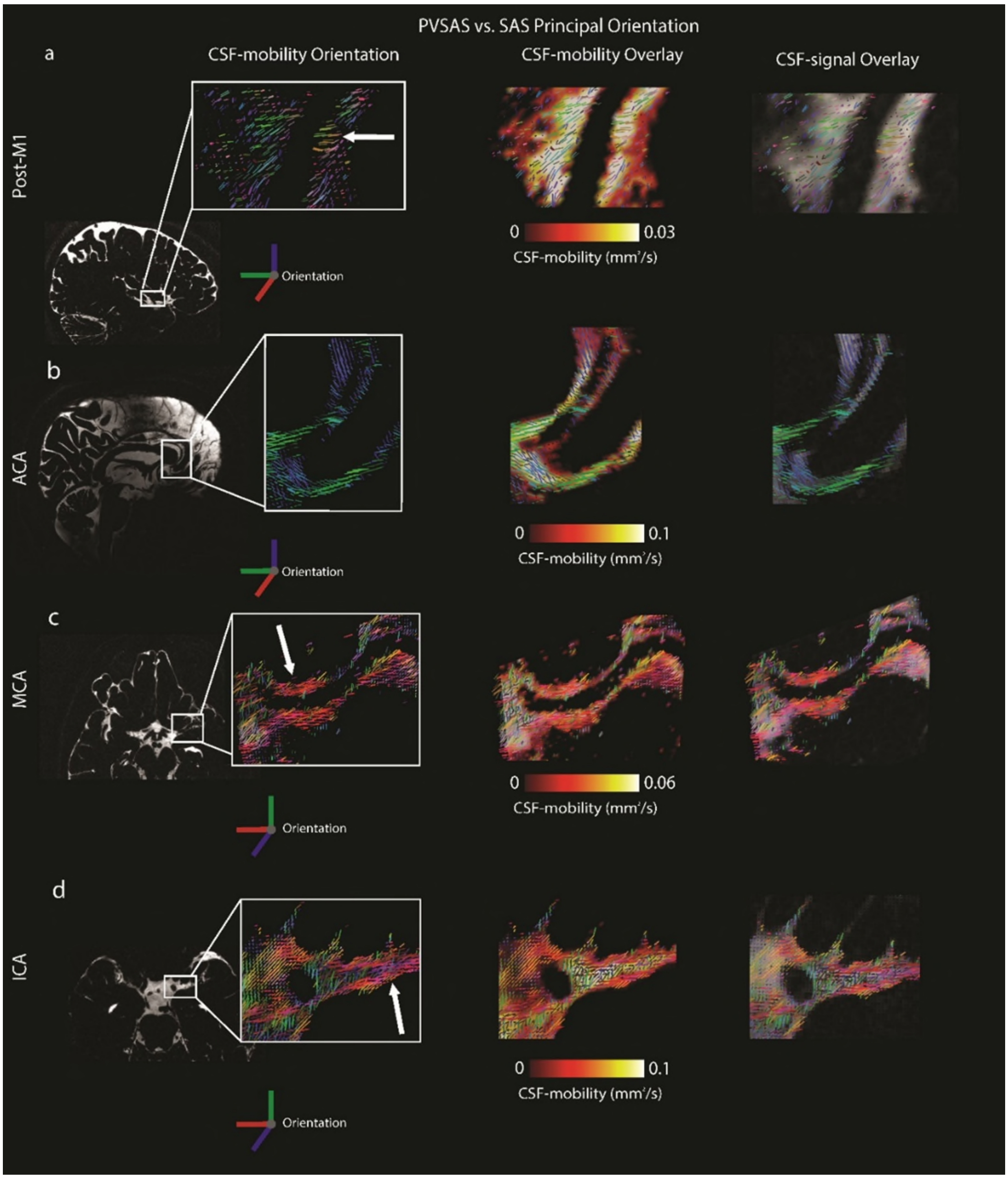

Figure 5 | Principal orientations within the perivascular subarachnoid space (PVSAS).

Principal orientations are overlaid on a through-plane of the PVSAS, shown on both the CSF-mobility and the non-motion-sensitized (gray) images. Line colors indicate orientation: red=left-right, green=anterior-posterior, blue=superior-inferior. White arrows point to PVSAS.

a) In the post-M1 PVSAS, CSF principal orientation shows greater directional coherence, indicated by the blue and green lines. In the SAS, CSF principal orientation shows lower directional coherence, reflected by more heterogeneous lines.

b) In the ACA PVSAS, CSF principal orientation travels anterior-posteriorly and then travels around the vessel superior-inferiorly indicated by the green and blue lines; there is limited SAS present.

c) Around the M1, large red lines within the PVSAS indicate more directionality in the left/right direction. In the SAS near the M1, there is more heterogenous color distribution, meaning less directional coherence.

d) The ICA shows two-compartments in the CSF-mobility, which is reflected with the principal orientation: in the high CSF-mobility region, there is more directional coherence, and lower directional coherence in the lower CSF-mobility region.

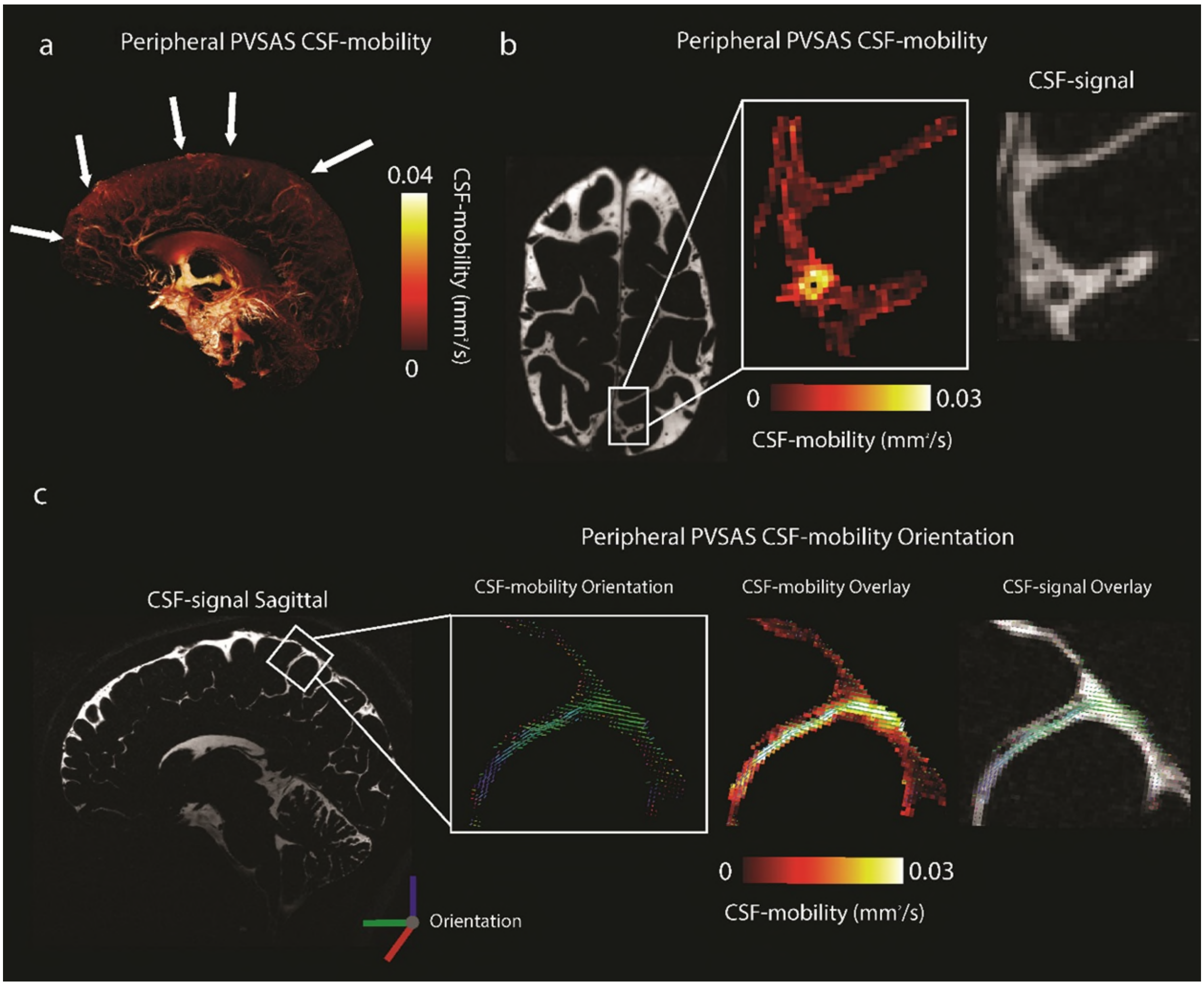

Figure 6 | Perivascular subarachnoid space (PVSAS) near the superior sagittal sinus (SSS).

a) Whole-brain example from a subject exhibiting numerous high CSF-mobility PVSAS near the SSS (white arrows).

b) Peripheral high CSF-mobility PVSAS near the SSS and the non-motion-sensitized scan (gray) confirms the presence of CSF, indicating that reduced mobility is not due to the absence of fluid.

c) Principal orientation of CSF-mobility around peripheral PVSAS. In the peripheral PVSAS, CSF principal orientation travels anterior/posteriorly and then travels around the vessel superior/inferiorly indicated by the green and blue lines. In the SAS, there is lower directional coherence. Lines are overlaid on the CSF-mobility and the non-motion-sensitized (gray) images. Line colors indicate orientation: red=left-right, green=anterior-posterior, blue=superior-inferior.

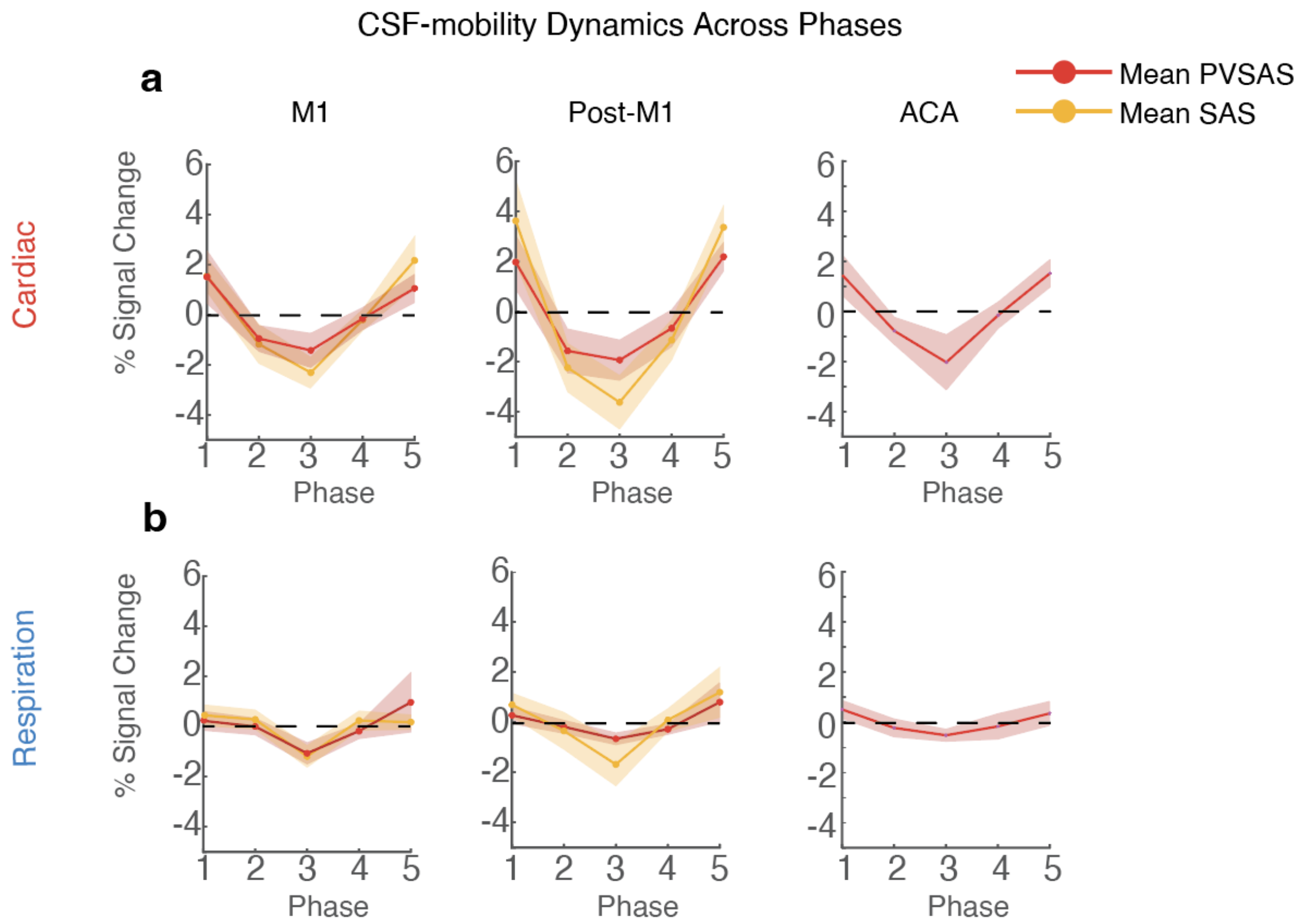

Figure 8 | Cardiac and respiration modulate CSF-mobility across phases in the perivascular subarachnoid space (PVSAS) and the subarachnoid space (SAS). CSF-mobility signal change from the mean value over phases (in %). Each line shows the mean across individuals and the shaded error areas represent confidence intervals of SD×1.96/√n (n=11).

a) Cardiac dynamics modulate PVSAS signal across CSF-mobility phases in M1, post-M1, and ACA. However, the relative percent signal change does not differ significantly between PVSAS and SAS in the M1 and post-M1.

b) Respiratory dynamics produce smaller modulations across phases in M1, post-M1, and ACA, with no significant differences in relative percent signal change between PVSAS and SAS in the M1 and post-M1.

2. Because CSF dynamics are already known to be tightly coupled to arterial pulsatility, the finding of higher mobility around arteries is expected and adds little novelty. What would be more compelling is a quantitative analysis relating CSF-mobility to arterial size and pulsatility, ideally through systematic mapping and segmentation of both large and small arteries.

We absolutely agree that CSF dynamics are influenced by arterial pulsatility, and we appreciate the reviewer's suggestion to relate CSF-mobility more directly to arterial size and pulsatility. We agree that a systematic, quantitative understanding CSF-mobility in the PVSAS vs the SAS is valuable, and we have therefore implemented quantitative measurements (Figure 4) in order to demonstrate quantitative differences between the PVSAS and SAS along the M1 and post-M1.

The literature indicates that the ICA, M1, M2, and ACA exhibit different diameters[14]. We now explicitly examined the CSF-mobility around the M1, post-M1, and ACA and did a quantitative comparison (Figure 4c). PVSAS CSF-mobility changes depending on the vasculature. Around the M1 and ACA, there is high CSF-mobility (M1 mean±SD=0.043±0.005 mm^2/s, ACA mean±SD =0.04±0.006 mm^2/s). Stepping down the vasculature tree to the post-M1 PVSAS, with a mean value of 0.03±0.008 mm^2/s there is a significant decrease in CSF-mobility (M1 vs. post-M1 p=0.0015, ACA vs. post-M1 p=0.0012, paired t-test, Bonferroni corrected, 3 tests) (Figure 4c).

However, the novelty of our findings lies not only in the general observation that CSF motion occurs near arteries, but in the spatial precision and temporal resolution with which CSF-STREAM can capture these dynamics. Especially, the observation of a sharp, stepwise decrease in mobility provides further evidence to the presence of PVSAS and a possible membrane.

Specifically, CSF-STREAM allowed us to quantify CSF-mobility at multiple PVSAS locations, including both proximal and distal segments of major cerebral arteries, where intrathecal and traditional MRI methods have limited sensitivity. This enables visualization of distinct mobility PVSAS patterns along different portions of the MCA and ACA (Figure 7), which has not been previously demonstrated. Additionally, we agree that developing additional tools to understand the relationship between CSF-mobility, arterial diameter, and pulsatility would be valuable in the future.

New text:

"When comparing the PVSAS across vessels, there is higher CSF-mobility around the M1 and ACA (M1 mean=0.043±0.005 mm^2/s, ACA mean=0.04± 0.006 mm^2/s) compared to the post-M1 PVSAS (post-M1 mean=0.03±0.008 mm^2/s) (M1 vs. post-M1 $p=1.49x10^{-3}$, ACA vs. post-M1 $p=1.12x10^{-3}$, paired t-test, Bonferroni corrected, 3 tests) (Figure 4c)."

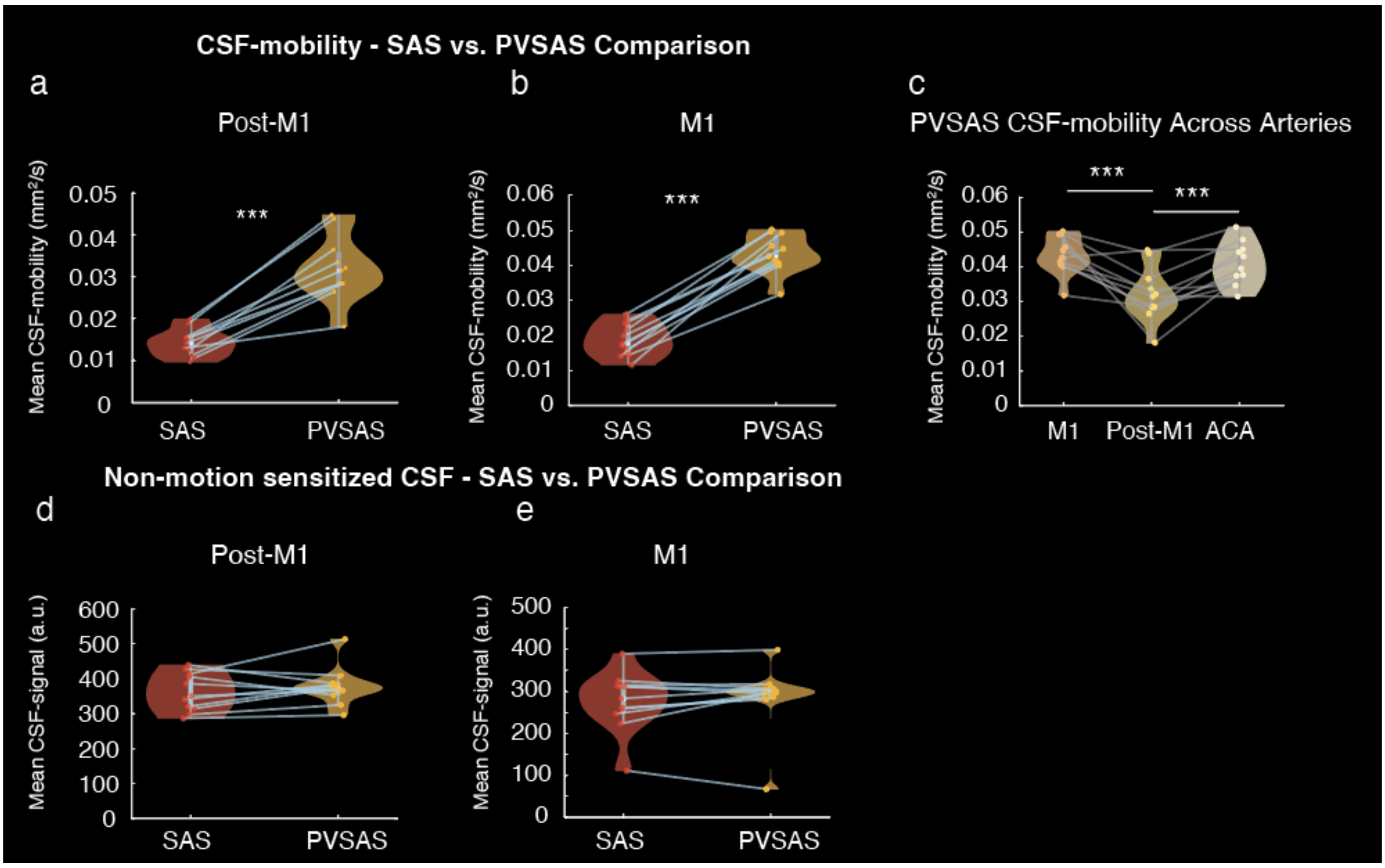

Figure 4 | Elevated CSF-mobility in the perivascular subarachnoid space (PVSAS) relative to the subarachnoid space (SAS).

a) Violin plot of post-M1 mean CSF-mobility in two subsections of the SAS and PVSAS (n=22 segments, 11 subjects, $p=2.0\times10^{-6}$, paired t-test, Bonferroni-corrected, 2 tests).

b) Violin plot of M1 mean CSF-mobility in two subsections of PVSAS and SAS (22 segments, 11 subjects, $p=6.5\times10^{-8}$, paired t-test, Bonferroni-corrected, 2 tests).

c) PVSAS CSF-mobility values across M1, post-M1, and ACA, shows significantly more CSF-mobility in the M1 and ACA PVSAS than the post-M1 PVSAS (11 subjects, M1 vs. post-M1: $p=1.49\times10^{-3}$; M1 vs. ACA: p=0.192; post-M1 vs. ACA: $p = 1.24\times10^{-3}$, paired t-test, Bonferroni-corrected, 3 tests).

d/e) SAS and PVSAS ROIs of the post-M1 and M1 on non-motion-sensitized images show similar CSF signal intensity, indicating that differences in CSF-mobility between SAS and PVSAS is not due to change in CSF presence (11 subjects, post-M1 p=0.32, M1 p=0.32, paired t-test, Bonferroni-corrected).

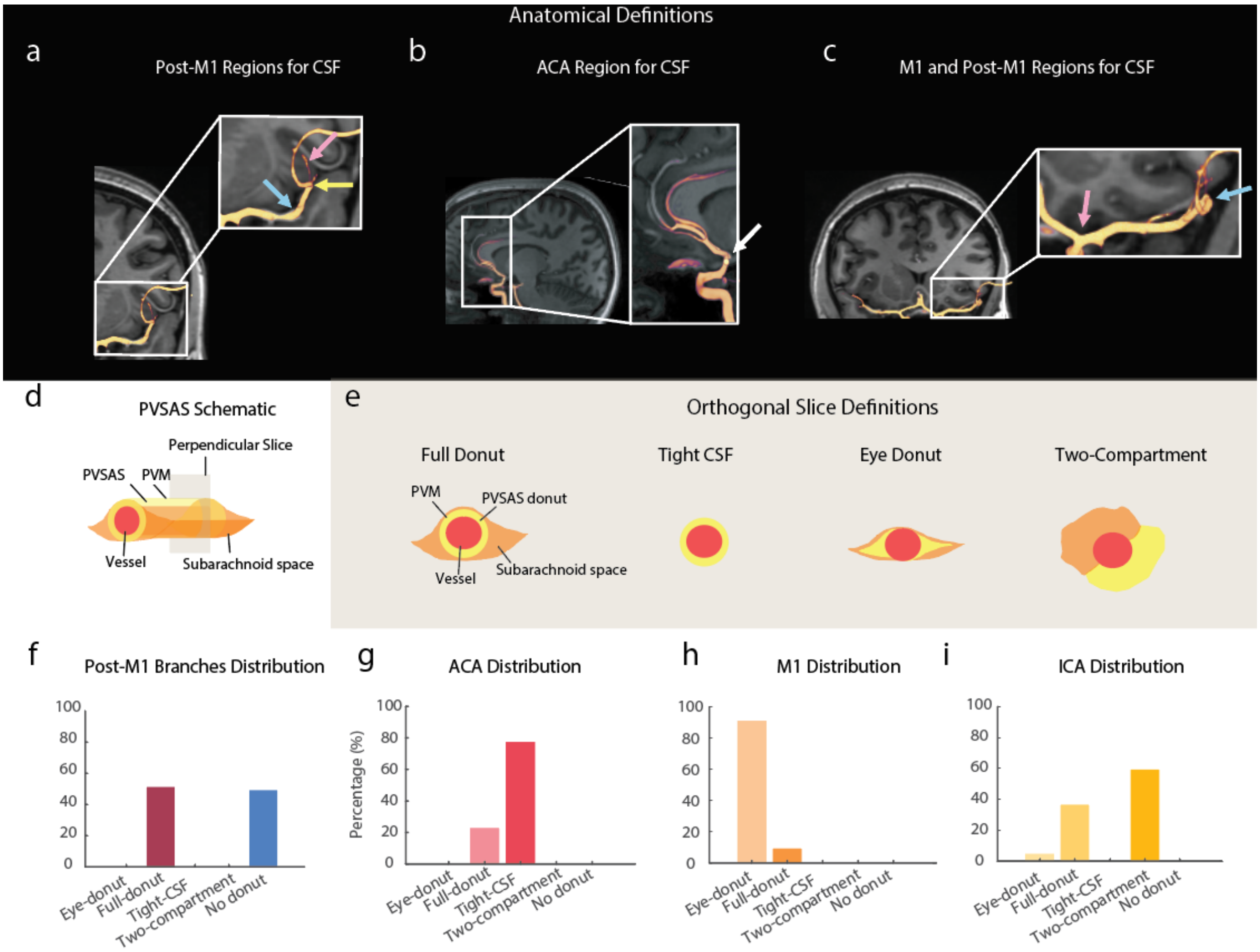

Figure 7 | Characterizing perivascular subarachnoid space (PVSAS).

a) 3D representation of arterial tree on top of a T1 anatomical transverse slice shows example of arteries that were rated in post-M1 distribution (pink arrow points to smaller artery). Blue arrow points to turn from M1 to M2. Yellow arrow points to turn from M2 to M3.
b) 3D representation of anterior carotid artery (ACA) on top of T1 anatomical sagittal image defines the ICA-to-ACA transition (white arrow).
c) 3D representation of middle cerebral artery (MCA) on top of T1 anatomical coronal image defines arterial transition ((internal carotid artery (ICA)-to-M1)) transition (pink), and M1-to-M2 transition (blue)).
d) Schematic illustration of perivascular subarachnoid space (PVSAS).
e) Cross-sectional schematics of CSF shapes.

f) Proportion of PVSAS located within the post-M1 arterial segments indicate 51% of visible arteries branching off the MCA have full-donuts (n=93 segments, 11 subjects).

g/h/i) Distributions of observed CSF shapes around ACA, M1, and ICA arteries (22 segments, 11 subjects).

3. Figures 2 and 3 present variations in the shapes and appearances of donut-like PVSAS structures, but the physiological significance of these observations is not explained. Do these differences simply reflect structural variation in arterial morphology and surrounding tissues, or are they meant to indicate functional aspects of CSF dynamics?

We think that the observed CSF differences reflect both structural variations in arterial morphology and corresponding functional aspects of CSF dynamics. Around the M1 segment, we generally observe less CSF and more frequent "eye-donut" configurations. Post-M1, where the SAS is larger and CSF presence is greater, we tend to observe "full-donut" structures, likely because there is more space for the PVSAS to expand. In the ACA, we typically observe smaller CSF regions and primarily high CSF-mobility without a surrounding SAS. We therefore hypothesize that PVSAS architecture arises from the interaction of two factors: (1) a perivascular membrane (PVM) and (2) local anatomical constraints, including the available SAS.

4. As presented, the motivation, hypothesis, and key message of these results are vague.

We appreciate the feedback. We have expanded the current text to extend upon our motivation and hypotheses.

We have changed:

Old text: "Based on the previous intrathecal contrast agent work, we hypothesized that the PVSAS would have higher CSF-mobility around branches of the MCA and ACA (M1-M4, A1- 87 A4)"

to

New text: "Based on the previous intrathecal contrast agent work, we hypothesize that the CSF-mobility maps would share a high level of similarity in their spatial patterns to early timepoint intrathecal contrast-enhanced scans. Because the intrathecal contrast travelled fastest along the PVSAS, we expect PVSAS in healthy controls to exhibit higher CSF-mobility around branches of the MCA and ACA (M1-M4, A1-A4) compared with the adjacent SAS.

To test these hypotheses exploratorily, we first compare CSF-STREAM CSF-mobility maps in healthy controls to intrathecal contrast-enhanced scans from reference patients. Given that post-M1 (defined as M2-M4) arteries showed the highest incidence of PVSAS in the intrathecal scans, we then examine the characteristics of CSF-mobility around the post-M1 arteries. Specifically, we hypothesize that CSF-mobility would be significantly higher within the PVSAS than in the surrounding SAS. We further hypothesize that CSF-mobility principal orientation will be more homogenous within the PVSAS compared to the SAS, reflecting an anatomical constraint, and that transitions at the ICA bifurcation would mark the emergence of distinct PVSAS compartments. Motivated by recent reports of arachnoid cuff granulation (ACE) points[1], we then examine arteries and veins adjacent to the superior sagittal sinus (SSS) for PVSAS CSF-

mobility patterns. Finally, we examine possibly drivers of the CSF-mobility by looking at how cardiac and respiration dynamics modulate CSF-mobility within the PVSAS and SAS."

Reviewer #4 (Remarks to the Author):

I read with interest the study by Fultz and colleagues. It leverages an MRI-method developed by their group to measure CSF motion in the subarachnoid space. They show that CSF flow is predominantly alongside certain neuro-anatomical pathways, also in healthy individuals. The manuscript is well written, has convincing illustrations and is of interest to the CSF community (and beyond). I believe the manuscript could further benefit from amending additional details on methodology and more clearly claiming an exploratory nature. Find my detailed comments below.

1. I would briefly mention earlier in the main how many participants were included in your study.

Thank you for the suggestion. We have added the number of participants early in the text as follows as follows in bold:

"**In eleven healthy controls**, whole-brain comparisons revealed that CSF-mobility patterns closely resembled those reported in the intrathecal scans."

2. Please also briefly mention spatial and time resolution of CSF-STREAM. This is important information to put your findings into context.

We have added the spatial resolution (0.45mm isotropic) as follows in bold:

"To investigate this, we employed a novel neuroimaging technique called CSF-STREAM that assesses high resolution **(0.45mm isotropic)** CSF-mobility"

We added the time resolution that can be extracted from CSF-STREAM into the methods section:

"The CSF-mobility maps quantify average CSF motion over the acquisition period (38 minutes and 30 seconds)."

3. "In both modalities, sagittal views show prominent signal in the basilar cistern, indicating fast contrast spreading and high CSF-mobility" – this is a bit confusing. CSF-STREAM does not rely on contrast agent; or do you compare with former experiments including contrast (in non-healthy individuals)? Please clarify.

Thank you for pointing out the lack of clarity. Indeed, CSF-STREAM does not rely on a contrast agent but instead uses a long echo time (500 ms) to selectively isolate CSF signal from tissue and blood. Our intention with the original sentence was to highlight that CSF-STREAM produces

patterns of CSF motion comparable to those previously observed in intrathecal contrast studies in reference patients.

We have changed the sentence from:

Old text: “In both modalities, sagittal views show prominent signal in the basilar cistern, indicating fast contrast spreading and high CSF-mobility”

to the following for clarity:

New text: “In intrathecal CSF scans, sagittal views show prominent signal in the basilar cisterns, reflecting rapid contrast transport. A similar pattern is observed in the CSF-mobility scans, which, without using contrast, show elevated CSF-mobility (~0.03-0.06 mm^2/s) in the basilar cisterns (Figure 1a,d; Supplemental Figure 2).”

4. “Mean CSF-mobility shows an increase in the PVSAS compared to the SAS (Figure 2e, p=2.0x10-6104 , paired t-test, Bonferroni-corrected).” – please add n per group here as well. Also, I’d recommend that you add the boxplots for these two groups with individual data points to the supplementary data. This also applies to other statistical test values you report. Also, please specify in the methods how many tests you ran.

Thank you for the suggestion. We have added the sample size to the text and figure legends. We also clarified the number of tests and opted to keep the violin plots that include individual data points for consistency with the rest of the manuscript. However, the violin plots have now been moved to their own figure; and within the figure legend is an example of the added sample size and number of tests:

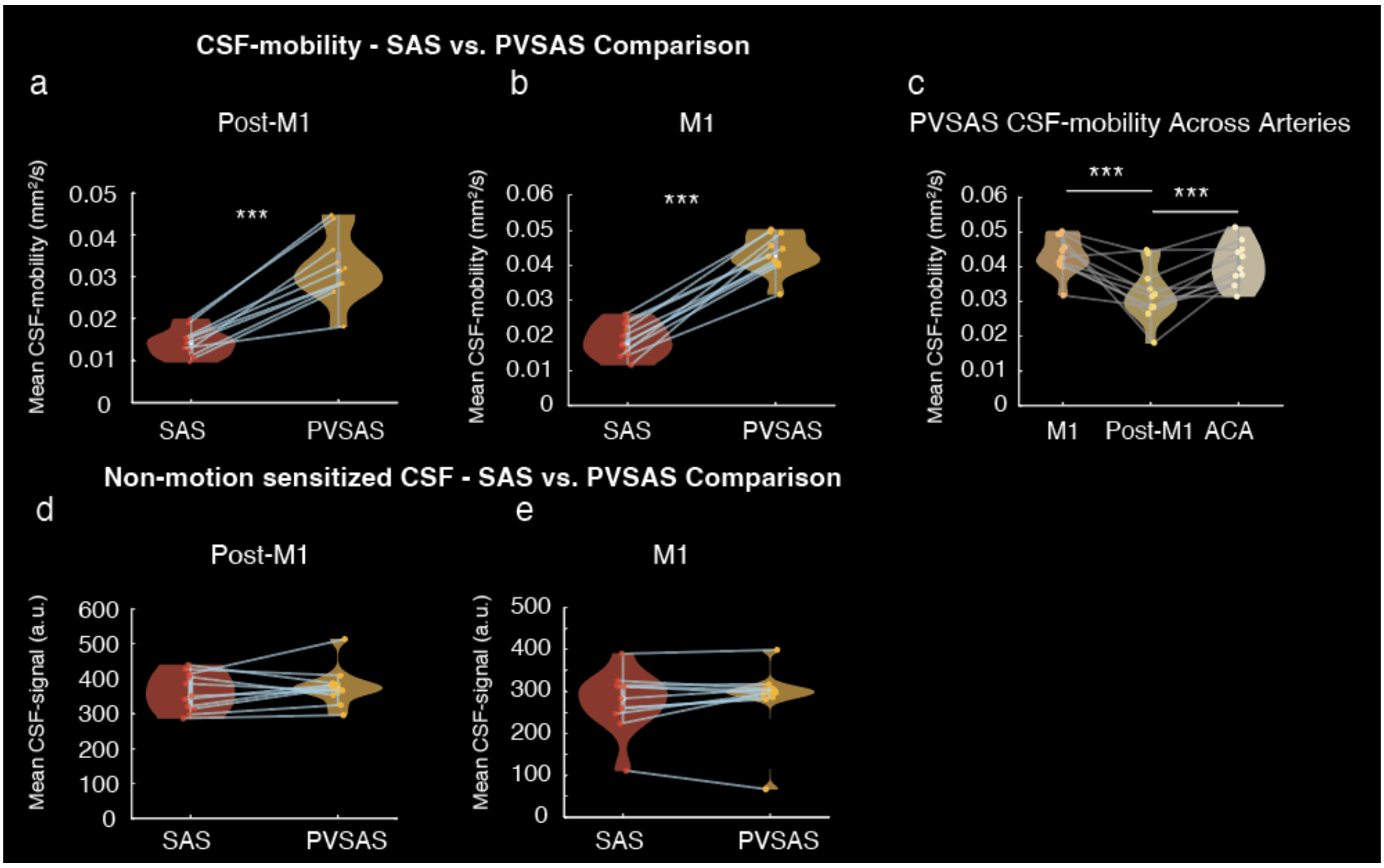

Figure 4 | Elevated CSF-mobility in the perivascular subarachnoid space (PVSAS) relative to the subarachnoid space (SAS).

a) Violin plot of post-M1 mean CSF-mobility in two subsections of the SAS and PVSAS (n=22 segments, 11 subjects, $p=2.0 \times 10^{-6}$, paired t-test, Bonferroni-corrected, 2 tests).

b) Violin plot of M1 mean CSF-mobility in two subsections of PVSAS and SAS (22 segments, 11 subjects, $p=6.5 \times 10^{-8}$, paired t-test, Bonferroni-corrected, 2 tests).

c) PVSAS CSF-mobility values across M1, post-M1, and ACA, shows significantly more CSF-mobility in the M1 and ACA PVSAS than the post-M1 PVSAS (11 subjects, M1 vs. post-M1: $p=1.49 \times 10^{-3}$; M1 vs. ACA: p=0.192; post-M1 vs. ACA: $p=1.24 \times 10^{-3}$, paired t-test, Bonferroni-corrected, 3 tests).

d/e) SAS and PVSAS ROIs of the post-M1 and M1 on non-motion-sensitized images show similar CSF signal intensity, indicating that differences in CSF-mobility between SAS and PVSAS is not due to change in CSF presence (11 subjects, post-M1 p=0.32, M1 p=0.32, paired t-test, Bonferroni-corrected).

5. "When examining the CSF-mobility by visual inspection in eleven healthy subjects, we consistently saw four distinct patterns related to PVSAS that we classified based on spatial distribution and CSF-mobility changes: full-donut, tight-CSF, eye-donut, and two-compartment (Figure 3m,n)." – can you please add some actual imaging examples to strengthen this point further.

Thank you for your feedback. We have added additional examples to the supplemental for clarity (Supplemental Figure 3).

Additional PVSAS examples:

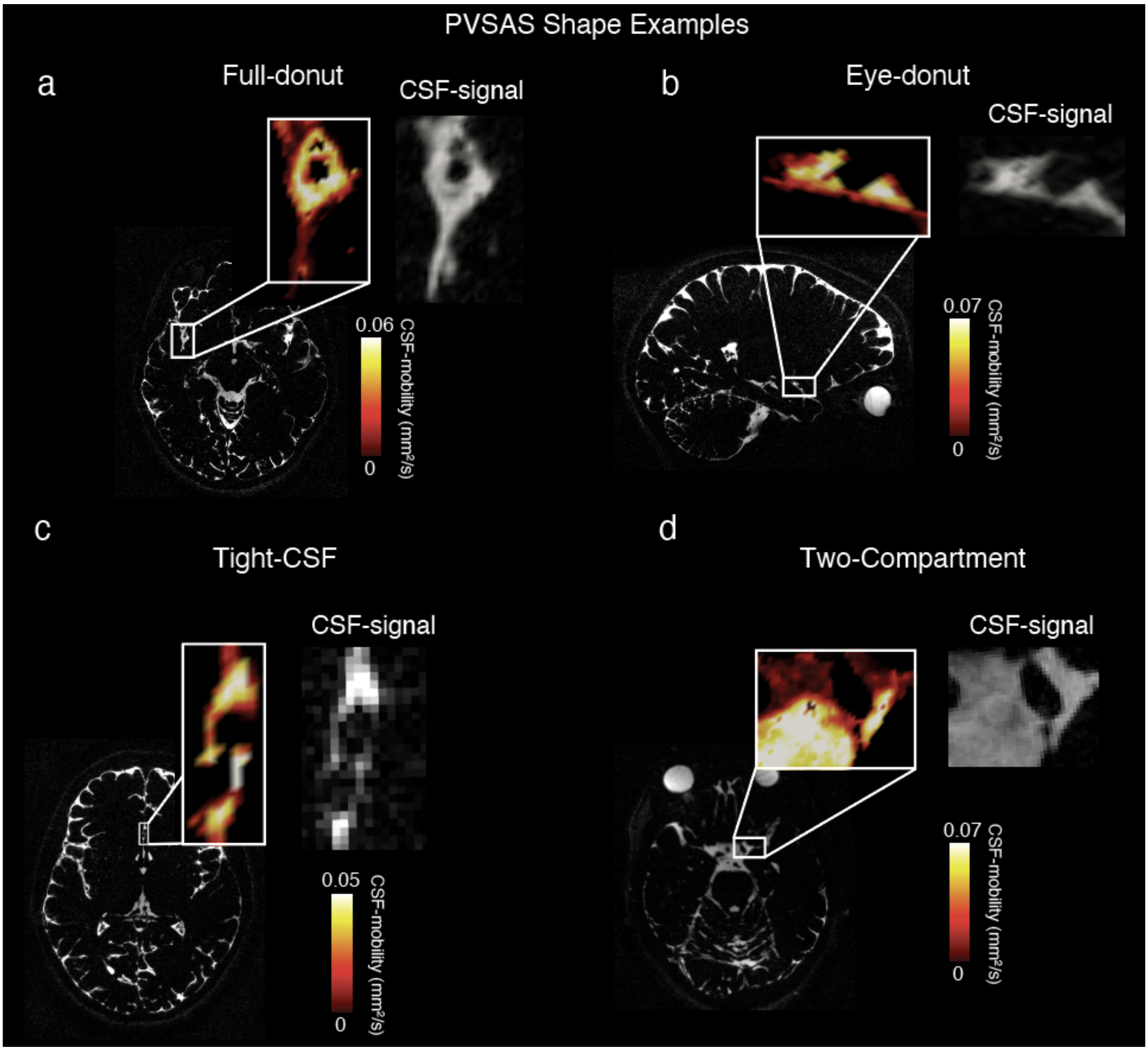

Supplemental Figure 3 | Examples of CSF structure across the ACA, M1 and ICA.

a) Full-donut PVSAS.
b) Eye-donut PVSAS.
c) Tight-CSF.
d) Two-compartment.

6. Have you performed a sample-size calculation? Please specify. Alongside this, it should be mentioned more clearly that this is an exploratory study, given the relatively small sample size and the absence of an a priori study protocol (I assume? Correct me if I am wrong).

Thank you for your comments and allowing us to add more clarity.

With the advice of our statistician, we did not perform an a priori power calculation because, at the time of study design, there was insufficient prior information to make a meaningful estimate of the expected effect size[15]. Nevertheless, we considered the research question important

enough to pursue empirically. Below we provide a power calculation that we would have done with the knowledge we now have.

We performed a sensitivity analysis for a paired design, using a range of conservative within-subject standard deviations (SD of differences) for CSF-mobility changes and calculated the number of subjects required. The table below shows the required number of subjects to detect a mean within-subject change of 0.02 mm^2/s^4, which showed the MCA SAS having a mean CSF-mobility of ~0.04 mm^2/s and the other regions having CSF-mobility mean values of less than 0.02 mm^2/s. With an expected 80% power and $\alpha = 0.05$.

Standard Deviation of Differences	Required Number of Subjects
0.0075	2
0.01	2
0.0125	4
0.015	5
0.0175	7
0.02	8

Which would make the twelve subjects provide adequate power while accounting for possible dropouts.

We have added to the text that this is an exploratory study. There was no a-priori protocol.

New text:
To test these hypotheses exploratorily, we compared CSF-STREAM derived CSF-mobility maps in healthy controls with intrathecal contrast-enhanced scans from reference patients.

More minor comments:
7. The text would benefit from subtitles (if the journal allows this) to make it more reader-friendly

We thank you for your feedback; We added the regular subtitles (introduction / results / discussion / methods) and implemented the following sections and sub-sections to make the text more reader-friendly:

CSF-mobility and contrast-enhanced scans show similar PVSAS

CSF-mobility around arteries show distinct PVSAS characteristics

High CSF-mobility PVSAS near the superior sagittal sinus

Cardiac and respiration dynamics modulate the PVSAS

8. Please make sure to make your Matlab code for statistical analysis available alongside your paper so other researchers are able to reproduce your findings (or re-use your code for their studies). I clicked on the link and it led to an error (maybe the repository is not set to public?)

Thank you for the feedback. We now have set the repository to public. There is a README file that shows what code makes what figure. The Matlab code for statistical analysis can be found in the following path:

https://github.com/ninafultz/csf_donuts/tree/master/figures

9. I sign my review: Benjamin Ineichen, Department of Clinical Research, University of Bern, Switzerland

Thank you for your comments and feedback!

Reviewer #4 (Remarks on code availability):

10. Link did not work (repository set to private?)

We thank you for pointing out the private repository. We now have set the repository to public.

References

1. Smyth, L. C. D. *et al.* Identification of direct connections between the dura and the brain. *Nature* **627**, 165–173 (2024).
2. Farrell, J. A. D. *et al.* Effects of SNR on the Accuracy and Reproducibility of DTI-derived Fractional Anisotropy, Mean Diffusivity, and Principal Eigenvector Measurements at 1.5T. *J Magn Reson Imaging* **26**, 756–767 (2007).
3. Giannelli, M. *et al.* Dependence of brain DTI maps of fractional anisotropy and mean diffusivity on the number of diffusion weighting directions. *J Appl Clin Med Phys* **11**, 176–190 (2009).
4. Hirschler, L. *et al.* Region-specific drivers of CSF mobility measured with MRI in humans. *Nat Neurosci* 1–10 (2025) doi:10.1038/s41593-025-02073-3.
5. Plá, V. *et al.* Structural characterization of SLYM—a 4th meningeal membrane. *Fluids Barriers CNS* **20**, 93 (2023).

6. Møllgård, K. *et al.* A mesothelium divides the subarachnoid space into functional compartments. *Science* **379**, 84–88 (2023).

7. Eide, P. K. & Ringstad, G. Functional analysis of the human perivascular subarachnoid space. *Nat Commun* **15**, 2001 (2024).

8. Weed, L. H. Studies on Cerebro-Spinal Fluid. No. III. *J Med Res* **31**, 51–91 (1914).

9. Gray, H. *Anatomy of the Human Body*. (Lea & Febiger, 1924).

10. Mirza, M. *et al.* Variability in Intracranial Vessel Diameters and Considerations for Neurovascular Models: A Systematic Review and Meta-Analysis. *SVIN* **4**, e001177 (2024).

11. Warnert, E. A. H., Verbree, J., Wise, R. G. & van Osch, M. J. P. Using High-Field Magnetic Resonance Imaging to Estimate Distensibility of the Middle Cerebral Artery. *Neurodegener Dis* **16**, 407–410 (2016).

12. Yildiz, S. *et al.* Quantifying the influence of respiration and cardiac pulsations on cerebrospinal fluid dynamics using real-time phase-contrast MRI. *Journal of Magnetic Resonance Imaging* **46**, 431–439 (2017).

13. Fultz, N. E. *et al.* Coupled electrophysiological, hemodynamic, and cerebrospinal fluid oscillations in human sleep. *Science* **366**, 628–631 (2019).

14. Huck, J. *et al.* Accurate and fully automated diameter measurements of Circle of Willis arteries on MRA imaging. *J Cereb Blood Flow Metab* **45**, 1774–1784 (2025).

15. Dziak, J. J., Dierker, L. C. & Abar, B. The Interpretation of Statistical Power after the Data have been Gathered. *Curr Psychol* **39**, 870–877 (2020).

RESPONSE TO THE REVIEWERS' COMMENTS

We appreciate the additional thoughtful reviewer comments. The reviewers raised several comments that helped to improve the manuscript, and we have addressed them. We have revised the manuscript with the new text additions written in red, as discussed in detail below. Relevant figures and any new text added to the manuscript are also included beneath each reviewer section.

REVIEWER COMMENTS

Reviewer #1 (Remarks to the Author):

Overall, the authors have done a good job addressing my previous concerns. The rationale, methodology and findings are now much clearer. The findings presented here are very interesting and would be a welcome addition to the literature where there is a need for novel and non-invasive methods to study CSF-ISF exchange in the human brain.

Thank you for your feedback.

Major comment:

1. For me, the most impactful finding of this work is that the CSF STREAM motility maps spatially agree with the para-arterial channels that preferentially transport the gad tracer identified from the intrathecal contrast-enhanced data sets. An important aspect of this observation is that the CSF STREAM motility maps and not just the CSF images spatially correlate to the DCE data. This concept is well illustrated in Figure 2F vs Figure 2G (where the 'CSF signal' images are also shown and do not spatially correlate as well to the DCE images). If this summation is correct, the CSF STREAM method could be used to map functionally relevant paravascular pathways non-invasively (and not just a long-TE high res scan to map paravascular spaces [ie the 'CSF signal' images]). I can see that the authors have added additional data in support of this conclusion (e.g supplementary Figure 3) but I still feel that showing more comparative data from more subjects using the same format in e.g. Figure 2F vs Figure 2G would help convince readers as to the robustness of this finding. As currently, the reader may be sceptical about this finding if only data from 2-3 subjects of the ~11 in each group are shown in the paper.

We are thankful for your input. We now have changed Figure 2d,h and Figure 3j,k to have intrathecal data with the same orientation and field-of-view as the CSF-STREAM data (orthogonal and through plane), using the same visualization program (Paraview). We have also added an additionally intrathecal example with the same format as the CSF-mobility (Figure 2i-l).

In order to show that the ACA and MCA PVSAS examples are representative of the whole cohort, we have now added additional ACA, MCA, and post-M1 PVSAS examples

(Supplemental Figure 9). We now show all subjects between the paper and supplemental. Additionally, we show examples of the full-donut PVSAS from sagittal view (Supplemental Figure 9i) and coronal view (Supplemental Figure 9j,k) to show that the PVSAS can be visualized from different orientations.

Text added in the methods to address the above concerns:

"All subjects are represented in the visualizations between the main text and supplemental."

2. Directly related to this, the way the data from the two types of scans are compared could be more consistent. For example, in Figure 2 b it is coronal and in Figure 2 C is sagittal and in Figure 3 the ICA comparison is missing. As I described in my initial review it would be helpful if the CSF STREAM data was compared to the DCE data using the same orientation and field of view in the 'zoom in' images so that the reader can more easily visually compare the images.

We greatly appreciate your input. We now have changed Figure 2d,h and Figure 3j,k to add intrathecal data with the same orientation and field-of-view as the CSF-STREAM data, including both orthogonal and through plane views. Additional intrathecal and CSF-mobility scans have also been added (Figure 2i-l; Supplemental Figure 4e,f).

Thank you for this suggestion, we agree the comparison of the CSF-STREAM with the intrathecal contrast scans is now much clearer to see. Additionally, we also added full-donut PVSAS from sagittal view (Supplemental Figure 9i) and coronal view (Supplemental Figure 9j,k) to show that the PVSAS can be visualized from different orientations.

For Figure 3c/f – the ICA CSF-STREAM example – there is no intrathecal contrast comparison because intrathecal contrast reaches the ICA too quickly (less than 10 minutes) and fills the whole compartment, precluding spatial assessment of PVSAS with contrast at the ICA level.

We have added the following text to describe why there is no ICA comparison:

"Finally, we were unable to directly compare CSF-mobility with intrathecal imaging for the ICA, because intrathecal contrast reaches it too rapidly and fills the compartment globally, precluding spatial assessment of PVSAS with contrast at the ICA level."

Minor comments:

3. The discussion is currently quite brief and could be more detailed in places: It would be helpful to explain exactly what is measured by the method (ie why is motility reported in mm2/s) as those unfamiliar with the history of the method may be confused by this.

We have added the additional text to the discussion to clarify what CSF-STREAM is, and what some of the limitations of the study were (also in response to further suggestions below):

"The CSF-mobility metric measured using CSF-STREAM is computed in a similar manner to an apparent diffusion coefficient acquired at a very low b-value[1]. This low b-value motion sensitizing strategy primarily encodes for CSF flow (whether bulk, parabolic, oscillatory or random) rather than diffusion[2,3]. This is also reflected in the CSF-mobility values measured in SAS/PVSAS (~10-50×10^{-3} mm^2 s^{-1}) which are more than ten times higher than brain tissue water diffusion (~1 × $10^{-3}$$mm^2$ s^{-1})[4]. However, CSF-STREAM does not measure phase accrual and therefore cannot determine flow directionality (i.e. it cannot differentiate right-to-left from left-to-right flow, but it can show increased mobility in the right-left direction versus e.g. feet-head direction)[1]. Previous intrathecal scans showing contrast travelling up along the PVSAS, with rapid arrival in the ACA and MCA (~23 minutes to A1; ~38 minutes to M1)[5], also hint at possible net flow. Cardiac and respiratory cycles modulate CSF-mobility in both the SAS and PVS. If the measured CSF-mobility were purely diffusion-derived, cardiac and respiratory dynamics would be expected to have no effect[6–8].

In terms of limitations, beyond the lack of ability to detect net flow, it should be mentioned that we have a small cohort. However, this cohort size has been used previously to assess CSF dynamics[9,10]. Furthermore, the intrathecal images and non-invasive CSF-STREAM data were obtained from separate cohorts. Though a limitation, the consistency of PVSAS across both modalities supports the robustness of these findings. Although we implemented a quantitative pipeline, future work should automate the characterization of the PVSAS on vasculature across the brain. Moreover, we have a long scan time that could lead to motion artifacts but were careful to exclude any participants with too much motion[1]. Importantly, although the overall scan time is ~38.5 minutes, each sub-scan lasts ~5.5 minutes, making standard motion correction appropriate. Ongoing work aims at reducing the scan time to ~20 minutes through further acceleration[11], and this shorter protocol will be implemented in future clinical studies[12–14]. Additionally, we have low temporal resolution when parsing the cardiac and respiration dynamics, but it has been shown previously that this temporal resolution is sufficient to detect physiological modulation above noise levels[1]. Finally, we were unable to directly compare ICA CSF-mobility with intrathecal imaging[5], because intrathecal contrast reaches the ICA too rapidly and fills the compartment globally, precluding spatial assessment of CSF with contrast at the ICA level."

4. To my understanding the CSF-STREAM is quite a long acquisition. Could the authors recommend an acquisition scheme to quickly capture the motility data that spatially correlates with the paraarterial locations identified on the DCE scans ie if you do not have to precise measure the FA/ tensor and just 'ADC' could you make the scan quicker?

We have been able to shorten the sequence by further accelerating the original CSF-STREAM sequence (from the original 15x acceleration to 32x acceleration) and find that we can shorten the scan to approximately 20 minutes with minimal changes in image

quality[11]. This newly accelerated sequence is being applied to new cohorts[12–14]. Additionally, the scan time could also be reduced by decreasing the resolution. Because the PVSAS is quite large, a (reasonable) reduction in resolution should not affect the ability to visualize the PVSAS around the MCA and ACA.

We have added some additional text in the discussion to address this comment:

"Ongoing work aims at reducing the scan time to ~20 minutes through further acceleration[11], and this shorter protocol will be implemented in future clinical studies[12–14]."

5. "in comparison with the post-M1 PVSAS. Beyond the major vascular tree, the peripheral high-mobility PVSAS were found heterogeneously around both arteries and veins and only seen in a minority of subjects.' Could the authors reference the appropriate results/Figures that support this statement and/or explain in more detail what evidence led to this summation as, for me, it is currently quite unclear.

These peripheral PVSAS can be seen in Figure 6a,b,c. There is high-CSF mobility that either is derived directly from around a major artery (as seen in the anterior part of the Figure 6a) or appear de novo around the superior sagittal sinus (posterior area in Figure 6a/b). In order to assess this, we marked the peripheral PVSAS on the CSF-mobility scans and then had two experienced neuroradiologists examine if these peripheral PVSAS were around veins, arteries, or near arachnoid granulations.

We have changed the wording to clarify what figures show the peripheral PVSAS:

Results: "Beyond the major vascular tree, the peripheral high-mobility PVSAS were found heterogeneously around both arteries and veins and often near arachnoid granulations, albeit only seen in a minority of subjects (Figure 6a,b,c). These peripheral high-mobility PVSAS often run from around a major artery to the periphery, or they appear de novo near the SSS."

6. For the data depicted in Figure 6, it should be made more clear that this was only observed in 2 of 11 subjects and therefore is not a robust observation. In the discussion could the authors discuss whether they think this was physiological or methodological?

We have added text in the figure title to note that we saw these results in a minority of subjects:

"Figure 6 | Perivascular subarachnoid space (PVSAS) near the superior sagittal sinus (SSS) observed in two out of eleven subjects."

We have changed the following text to address whether these peripheral PVSAS are physiological or methodological:

Old text:

“Beyond the major vascular tree, the peripheral high-mobility PVSAS were found heterogeneously around both arteries and veins and only seen in a minority of subjects. The heterogeneity of the high-mobility PVSAS near the SSS may correspond to a recently described leptomeningeal perivascular network involved in fluid shunting, CSF drainage, and immune surveillance[16].”

New text:

“Beyond the major vascular tree, the peripheral high-mobility PVSAS were found heterogeneously around both arteries and veins and often near arachnoid granulations, albeit only seen in a minority of subjects (Figure 6a,b,c). These peripheral high-mobility PVSAS often run from around a major artery to the periphery, or they appear de novo near the SSS. Interestingly, the example shown in Figure 6b shows the presence of several vessels close to each other (see CSF-signal insert in Fig. 6b), but higher CSF-mobility is only observed around one of the vessels, pointing towards a physiological explanation rather than methodological. The observations of peripheral PVSAS were sparse, but do not appear to reflect methodological artifacts. Signal changes in the non-motion-sensitized scan, head motion, and partial volume effects from adjacent vessels were each considered to exclude the possibility of an artifactual observation. Though they cannot be ruled out entirely, these effects would not be expected to act selectively on signal at the brain surface or around individual vessels. We applied conservative criteria for rating peripheral PVSAS (see methods). Importantly, image quality in both subjects was high, with minimal noise or artifacts. We therefore think these peripheral PVSAS may reflect true physiological CSF dynamics and could correspond to a recently described leptomeningeal perivascular network involved in fluid shunting, CSF drainage, and immune surveillance[15].”

7. Could the authors discuss the evidence for bulk vs diffusive motion of para-arterial fluid?

We have added the additional text to talk about bulk vs. diffusion:

“The CSF-mobility metric measured using CSF-STREAM is computed in a similar manner to an apparent diffusion coefficient acquired at a very low b-value. This low b-value motion sensitizing strategy primarily encodes for CSF flow (whether bulk, parabolic, oscillatory or random) rather than diffusion[2,3]. This is also reflected in the CSF-mobility values measured in SAS/PVSAS (~$10\text{-}50 \times 10^{-3}$ mm^2 s^{-1}) which are more than ten times higher than brain tissue water diffusion (~$1 \times 10{-}3 mm^2$ s^{-1})[4]. However, CSF-STREAM does not measure phase accrual and therefore cannot determine flow directionality (i.e. it cannot differentiate right-to-left from left-to-right flow, but it can show increased mobility in the right-left direction versus e.g. feet-head direction)[1]. Previous intrathecal scans showing contrast travelling up along the PVSAS, with rapid arrival in the ACA and MCA (~23 minutes to A1; ~38 minutes to M1)[5], also hint at possible net flow. Cardiac and respiratory cycles modulate CSF mobility in both the SAS and PVS. If the measured CSF mobility were purely diffusion-derived, cardiac and respiratory dynamics would be expected to have no effect[6–8].”

Below are all figures modified or mentioned above:

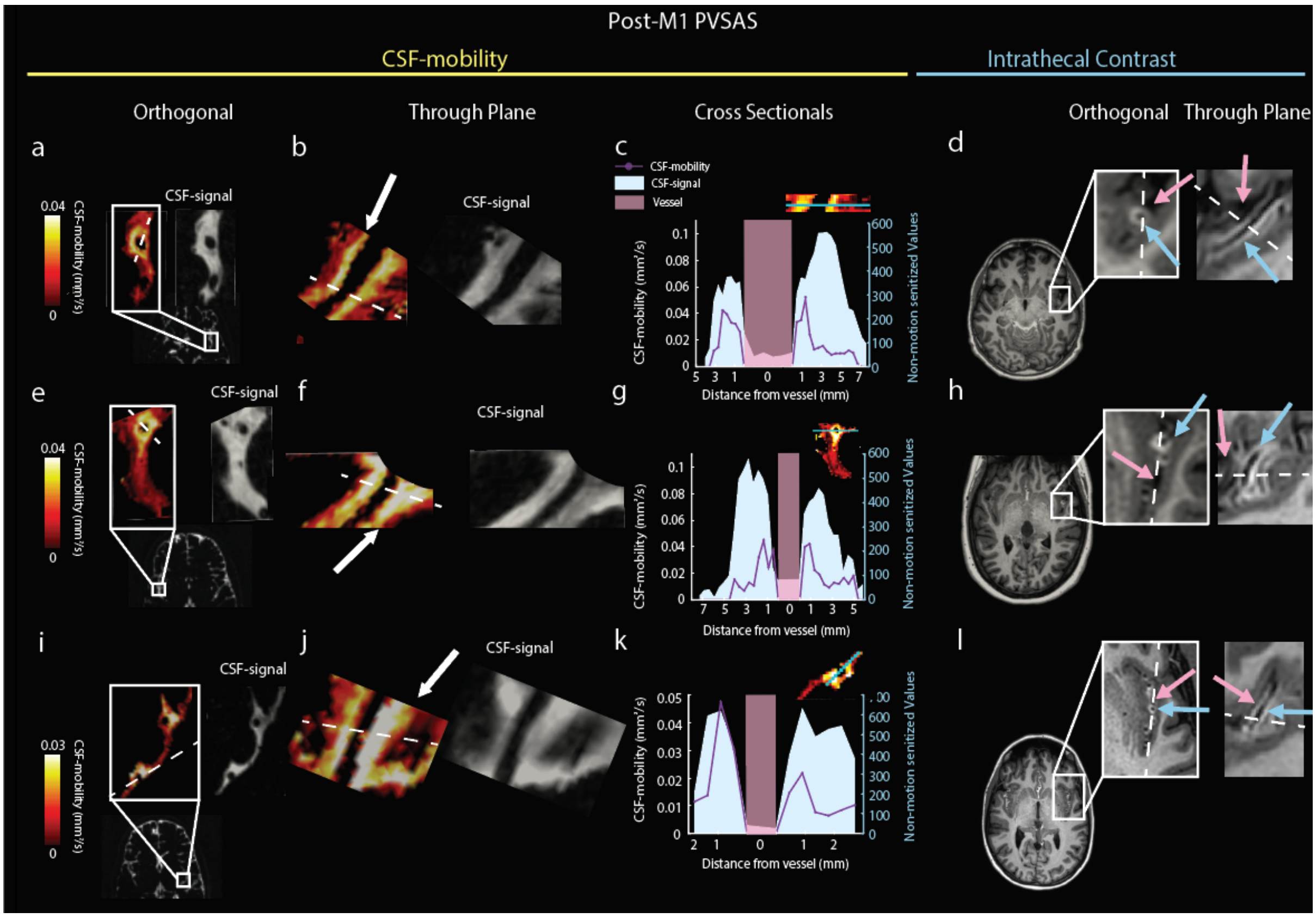

Figure 2 | Perivascular subarachnoid space (PVSAS) in the post-M1 (M2-M4) region.

a/e/i) *First column* shows full-donut PVSAS with high CSF-mobility surrounding post-M1 arteries. Non-motion-sensitized scans (gray) (referred to as 'CSF-signal') confirm equal CSF presence in both high- and low-mobility regions. Some of the PVSAS show some structural heterogeneity, indicating that the full-donut PVSAS can be asymmetric and deviate from perfect circularity (e.g., e, i). White boxes mark regions used for PVSAS extraction; dotted lines indicate locations of the corresponding through-plane images in second column.

b/f/j) *Second column* shows through-plane views of orthogonal CSF from the first column, displaying CSF-mobility, alongside matching non-motion-sensitized scans (gray). Dotted lines indicate locations of the corresponding orthogonal images in first column.

c/g/k) *Third column* presents representative cross-sectional plots from similar areas in the first and second columns. The pink bar marks the vessel; the x-axis shows distance from the vessel (mm), with CSF-mobility (purple, left y-axis) and non-motion-sensitized signal (blue, right y-axis). ROI location on the CSF-mobility map is indicated in the top right of the plot.

d/h/l) *Fourth column* shows post-M1 intrathecal contrast-enhanced PVSAS from patients from similar anatomical locations as in the first and second column (examples are from data used in Eide & Ringstad, 2024). Dotted white lines indicate intersecting locations. Pink arrow highlights the subarachnoid space (SAS), and the blue arrows show the contrast within the PVSAS.

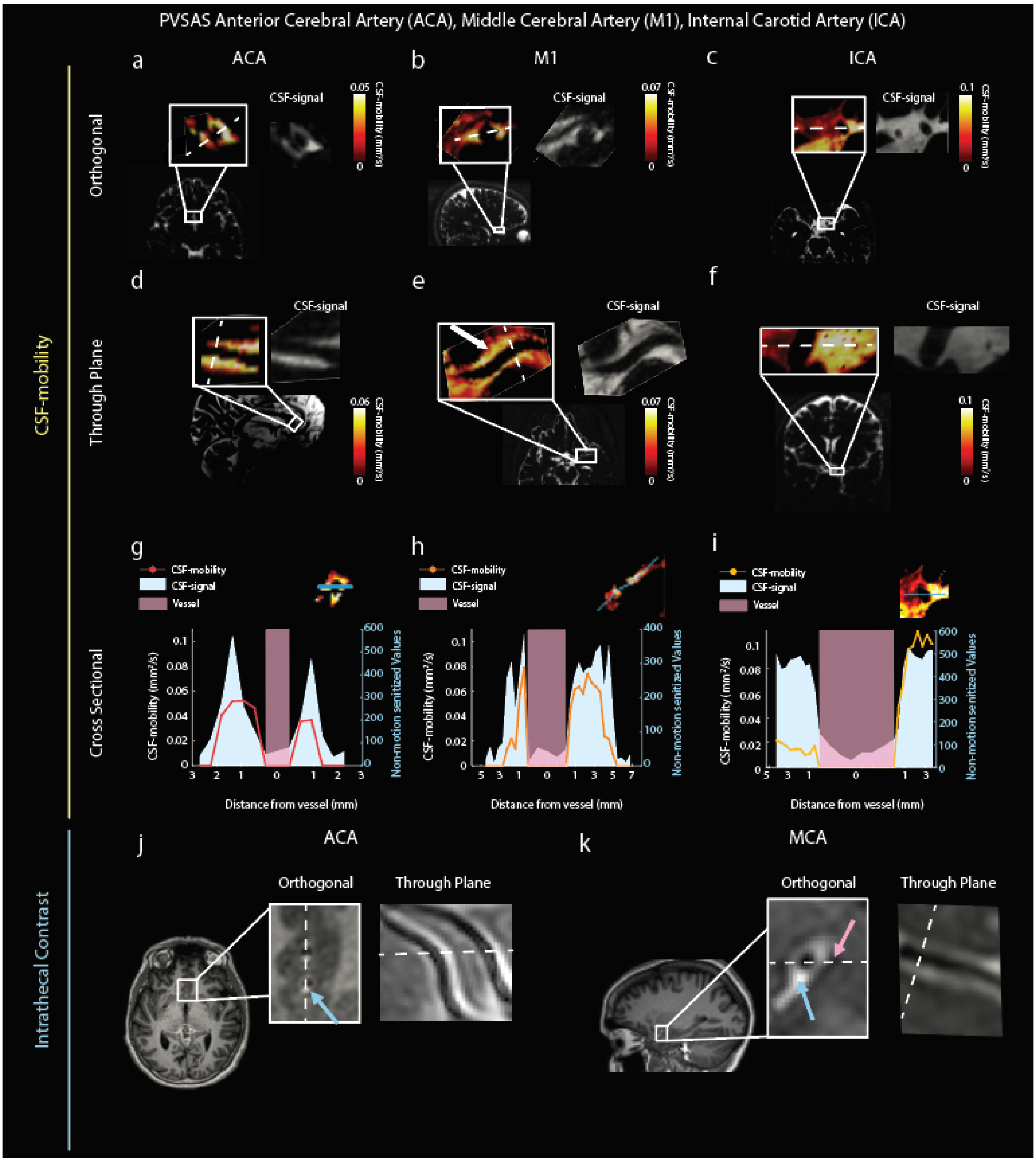

Figure 3 | Perivascular subarachnoid space (PVSAS) across the anterior carotid artery (ACA), M1 and internal carotid artery (ICA).

a) Example of an ACA CSF-mobility PVSAS, displaying tight-CSF in orthogonal image. The non-motion-sensitized scan (gray) (referred to as 'CSF-signal') shows a narrower CSF

space around the vessel compared to other locations, for example around the MCA. The white box marks the extraction site of the PVSAS; the white dotted line indicates the location of the through-plane image seen in ‘d’.

b) Example of an M1 CSF-mobility PVSAS showing high CSF-mobility near the artery in a bilateral triangular pattern within a region of lower CSF-mobility. The non-motion-sensitized scan (gray) confirms the presence of CSF in the zoomed-in view, indicating that reduced mobility is not due to a reduction in CSF signal. Identical layout to ‘a’.

c) Example of an ICA CSF-mobility two-compartment, showing asymmetric mobility: low CSF-mobility on one side of the artery and high on the other. The non-motion-sensitized scan (gray) confirms similar CSF signal on both sides of the artery. White box indicates the extracted region for through-plane image.

d/e/f) Through plane images of ACA, M1, and ICA CSF-mobility. The non-motion-sensitized scan (gray) shows CSF presence. White dotted line shows location of orthogonal cut from ‘a/b/c’.

g/h/i) Representative cross-sectional plots of the PVSAS shown in similar area to orthogonal cut for ACA, M1, and ICA above. Pink bar represents the vessel; the x-axis indicates distance from the vessel (mm), the left y-axis shows CSF-mobility, and the right y-axis shows non-motion-sensitized signal. ROI location is indicated in the corner.

j) ACA (A1) intrathecal contrast scan showing tight contrast-enhancement[5] near the vessel (blue arrow), and can be compared to the ACA PVSAS in ‘a’ and ‘d’. White dotted line indicates where orthogonal and through-plane images were extracted.

k) MCA intrathecal contrast scan shows high eye-shaped contrast-enhancement[5] next to the vessel (blue arrow), within a lower contrast area (pink arrow), and can be compared to M1 PVSAS in ‘b’ and ‘e’. White dotted line shows where the orthogonal and through-plane images were extracted.

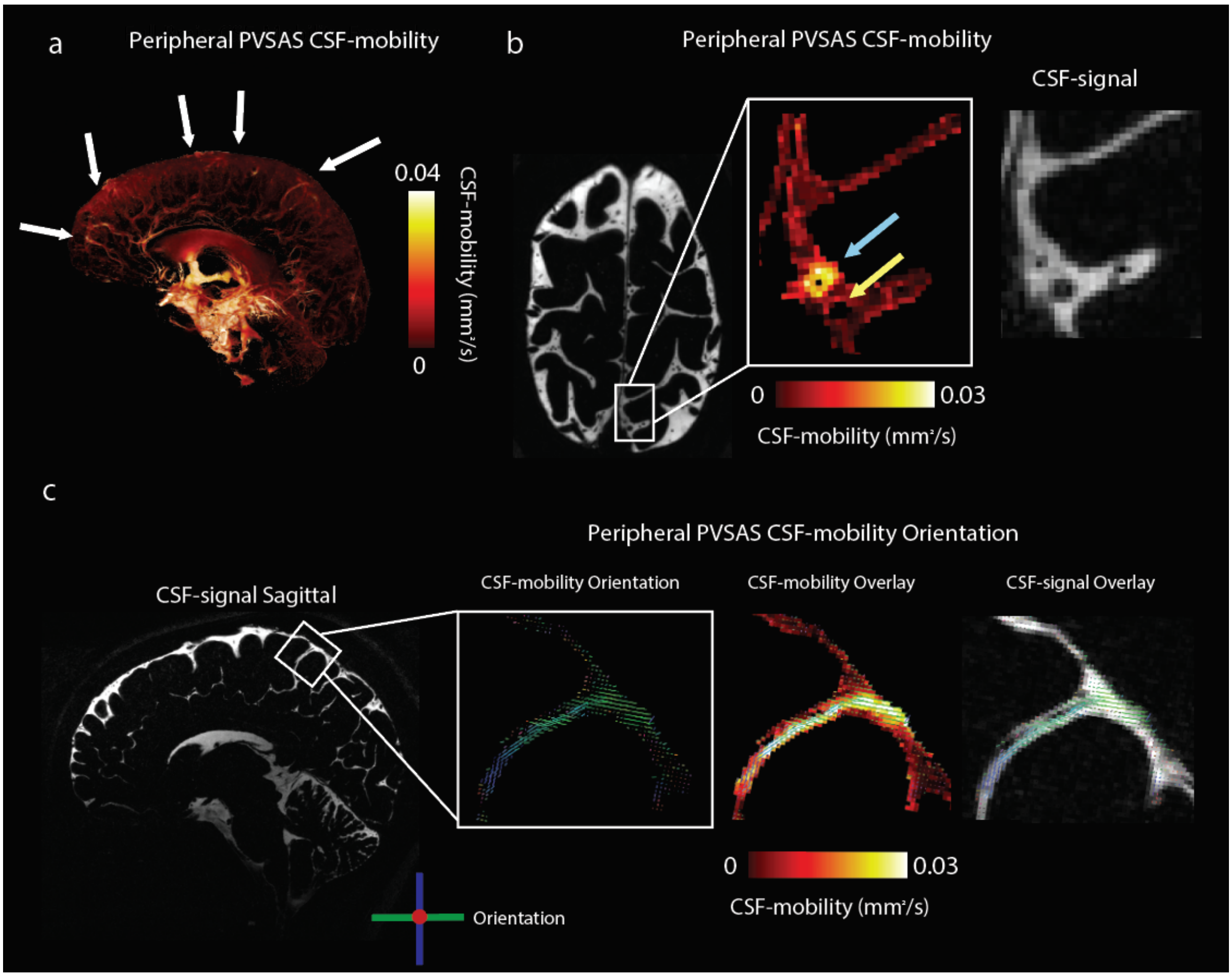

Figure 6 | Perivascular subarachnoid space (PVSAS) near the superior sagittal sinus (SSS) observed in two out of eleven subjects.

a) Whole-brain example from a subject exhibiting numerous high CSF-mobility PVSAS near the SSS (white arrows).

b) Peripheral high CSF-mobility PVSAS near the SSS and the non-motion-sensitized scan (gray) confirms the presence of CSF, indicating that reduced mobility is not due to reduced CSF-signal. Two adjacent vessels show that not all peripheral PVSAS have high CSF-mobility (blue arrow indicates vessel with high CSF-mobility; yellow indicates vessel with visible high CSF-mobility PVSAS).

c) Principal orientation of CSF-mobility around peripheral PVSAS. In the peripheral PVSAS, CSF mobility is first orientated anterior/posteriorly and then oriented around the vessel superior/inferiorly indicated by the green and blue lines. In the SAS, there is lower directional coherence. Lines are overlaid on the CSF-mobility and the non-motion-sensitized (gray) images. Line colors indicate orientation: red=left-right, green=anterior-posterior, blue=superior-inferior.

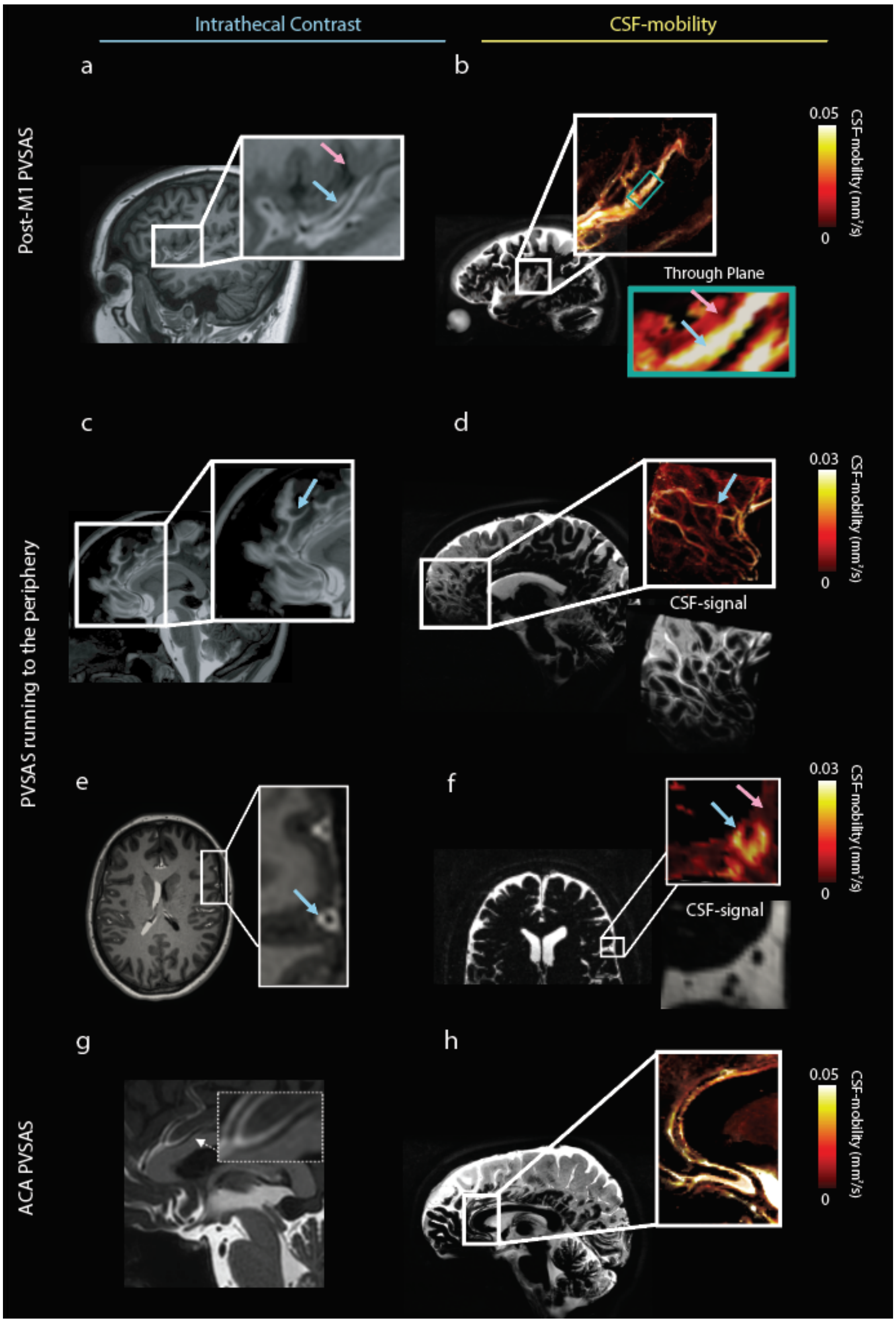

Supplemental Figure 4 | Intrathecal and perivascular subarachnoid space (PVSAS) comparisons.

a) Intrathecal contrast agent travelling along the post-M1 shows contrast in the perivascular subarachnoid space (PVSAS) close to the vessel (blue arrow), and no contrast in the subarachnoid space (SAS) (pink arrow).
b) CSF-mobility of the post-M1 PVSAS. Gray is MIP of non-motion sensitized scan, and in the white box is the CSF-mobility, showing MIP of post-M1 PVSAS. Green box shows cross sectional of post-M1 PVSAS show steep drop off between the PVSAS (blue) and the SAS (pink).

c) Intrathecal contrast agent scan shows contrast running to the periphery in the PVSAS (blue arrow).
d) CSF-mobility, showing PVSAS running to the periphery. Gray MIP of non-motion sensitized scan.
e) Intrathecal contrast agent PVSAS running to the periphery (blue arrow).
f) CSF-mobility PVSAS at the periphery (blue arrow) surrounded by SAS (pink arrow).
g) Intrathecal contrast agent runs along anterior carotid artery (ACA) shows the PVSAS.
h) CSF-mobility 3D-representation of ACA shows limited SAS around the PVSAS.

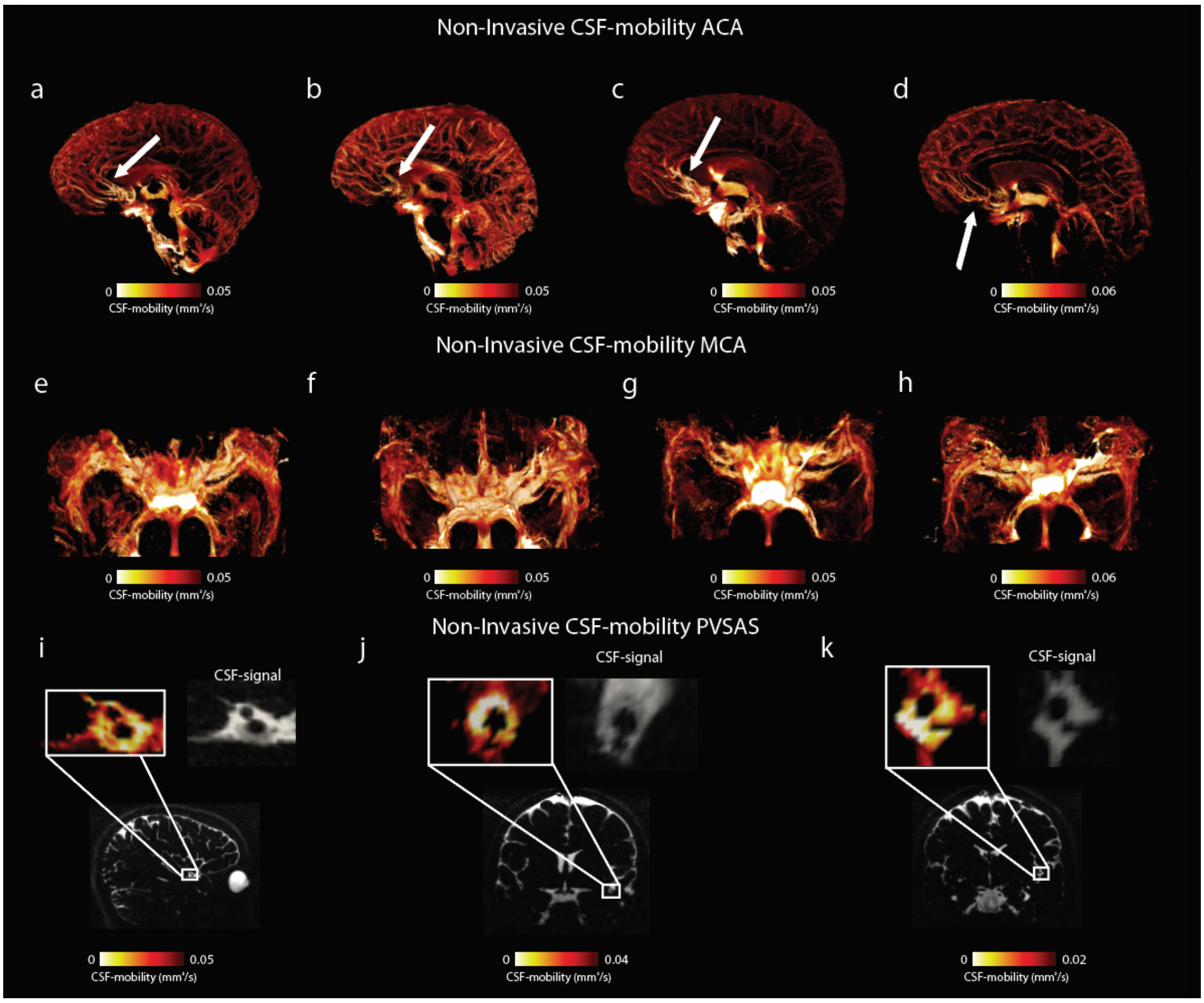

Supplemental Figure 9 | Additional examples of CSF-mobility perivascular subarachnoid space (PVSAS).

a-d) Additional sagittal views of CSF-mobility scan in healthy controls show perivascular subarachnoid space (PVSAS) surrounding the anterior carotid artery (ACA). White arrows highlight ACA.

e-h) Transverse views of CSF-mobility scan show high CSF-mobility PVSAS around the middle cerebral artery (MCA).

i-k) Examples of full-donut PVSAS with high CSF-mobility surrounding post-M1 arteries, next to matching non-motion-sensitized scans (gray). White boxes mark regions used for

PVSAS extraction; dotted lines indicate locations of the corresponding through-plane images in second panel.

Reviewer #3 (Remarks to the Author):

Here are some of my remaining comments:

1. The major claim of the paper is the observation of higher mobility in the PVSAS compared with the surrounding SAS using the CSF stream method, which is stated to be consistent with previous findings from contrast-enhanced studies investigating PVSAS and the PVSAS membrane. However, the comparison between the CSF stream method and the contrast-enhanced approach—the key component supporting this claim—has several limitations: i) the data are derived from different cohorts (CSF stream data from healthy controls vs. contrast enhanced data from patients), and ii) the comparison is primarily based on visual inspection of a small number of example images from limited subjects presented. A more quantitative and systematic comparison beyond visual inspection, would substantially strengthen the support for the main conclusion. These limitations should be addressed and also discussed in the discussion section.

We appreciate your feedback. We have now added more CSF-mobility and intrathecal data so that all subjects are represented visually between the manuscript and the supplemental (Figure 2i-l; Supplemental Figure 4; Supplemental Figure 9).

We have now done both a quantitative and systematic comparison of the PVSAS vs SAS in the CSF-mobility scans as follows:

1) We have implemented a semi-automatic pipeline to extract the CSF-mobility in concentric circles around the post-M1 vasculature (Supplemental Figure 7; Supplemental Figure 8). Using this analysis pipeline, we found similar results to our visual inspection, with higher CSF-mobility in the PVSAS than in the surrounding SAS.
2) As previously shown in the first revision, we have done cross-sectional plots that show the change in CSF-mobility around different vessels (Figure 2c,g,h; Figure 3g,h,i). We have also shown a quantitative comparison of CSF-mobility values in SAS and PVSAS for the M1 and post-M1 regions (Figure 4a,b,d,e). Finally, we have compared the PVSAS CSF-mobility values around the different vessels (M1 vs. post-M1 vs. ACA CSF-mobility values) (Figure 4c).

We have added additional texts in the results, discussion, and methods section on the concentric circles analysis:

Text added to address the number of visualizations of subjects:

"All subjects are represented in the visualizations between the main text and supplemental."

We have added this additional text to the results section:

“Semi-automated concentric circles around the post-M1 vasculature mirror these results, showing high CSF-mobility with a steep drop-off at ~1.5 mm distance from the vessel wall (Supplemental Figure 7f; 11 subjects, post-M1 p=6.0x10-4, paired t-test, Bonferroni-corrected, 2 tests; see also Supplemental Figures 7f, 8). Mean non-motion sensitized CSF-signal show no significant change between where the PVSAS is expected (0-1.5 mm) and the SAS (1.5-3 mm) (Supplemental Figure 7h; 11 subjects, post-M1 p=0.74, paired t-test; see also Supplemental Figures 7g and 8).”

We have explained how we made the concentric circles around the vessels in the methods:

“Vessel masks were first created by thresholding the registered T1 anatomical image, in which we can take advantage of the inflow effect which will make the major arteries (MCA, ACA) have bright signal. A seed point was placed at the internal carotid artery (ICA) and allowed to grow across the thresholded T1 volume, yielding an initial vessel mask. Any voxels within the initial vessel mask that overlapped with CSF signal, due to registration imperfections in the non-motion-sensitized image, were manually removed from the mask. This mask was combined with the thresholded non-motion-sensitized CSF signal image, and morphological operations (imfill, imclose; MATLAB2024a) were applied to close residual gaps. The vessel mask was then dilated and made to stop at the boundary of the combined mask, and the non-motion sensitized CSF signal was subtracted to isolate the vasculature area. Post-M1 segments of the MCA were manually delineated and extracted, and all vessel masks were visually inspected to exclude voxels falling outside the vessel boundary. Concentric rings were generated by iteratively dilating the post-M1 vessel masks (imdilate; MATLAB). This mask was applied to the non-motion-sensitized CSF-signal images and to the CSF-mobility maps (Supplemental Figure 1). CSF-mobility and CSF-signal values were extracted within each ring and plotted as a function of distance from the vessel. CSF-signal and mobility values were normalized subject-wise by the max peak. The M1 PVSAS was not included because of its ‘eye-donut’ shape.”

We have added an additional section on our limitations in the discussion:

“In terms of limitations, beyond the lack of ability to detect net flow, it should be mentioned that we have a small cohort. However, this cohort size has been used previously to assess CSF dynamics[9,10]. Furthermore, the intrathecal images and non-invasive CSF-STREAM data were obtained from separate cohorts. Though a limitation, the consistency of PVSAS across both modalities supports the robustness of these findings. Although we implemented a quantitative pipeline, future work should automate the characterization of the PVSAS on vasculature across the brain. Moreover, we have a long scan time that could lead to motion artifacts but were careful to exclude any participants with too much motion[1]. Importantly, although the overall scan time is ~38.5 minutes, each sub-scan lasts ~5.5 minutes, making standard motion correction appropriate. Ongoing work aims at reducing the scan time to ~20 minutes through further

acceleration[11], and this shorter protocol will be implemented in future clinical studies[12–14]. Additionally, we have low temporal resolution when parsing the cardiac and respiration dynamics, but it has been shown previously that this temporal resolution is sufficient to detect physiological modulation above noise levels[1]. Finally, we were unable to directly compare ICA CSF-mobility with intrathecal imaging[5], because intrathecal contrast reaches the ICA too rapidly and fills the compartment globally, precluding spatial assessment of CSF with contrast at the ICA level."

2. Thank you to the authors for addressing the concern regarding partial volume effects. Showing the principal orientation and the non-motion-sensitized images does help alleviate concerns about this potential confound.

Thank you for your feedback – your concerns strengthened the manuscript.

3. However, the argument regarding the impact of respiration and cardiac pulsation on high CSF mobility in PVSAS—and the conclusion that the observed higher mobility is not caused by cardiac or respiratory effects but by the presence of a membrane because PVSAS and SAS are affected similarly—is not yet fully convincing (Figure 8). Or if the key supporting argument is instead the presence of a sharp drop-off of the high mobility from PVSAS to SAS as mentioned in the response and manuscript, please clarify how "sharp" is defined quantitatively, what threshold defines a sharp transition, and what percentage of vessel segments demonstrate this sharp versus non-sharp pattern? This would support the conclusion beyond just reliance on visual inspection of the sharp drop-off and representative images.

We agree that the plots showing respiration and cardiac CSF-mobility in the PVSAS and SAS is not the key argument for a presence of a membrane. Instead, this data shows us that cardiac and respiration modulate both the PVSAS and SAS, but do not necessarily account for the large CSF-mobility change we see in the PVSAS. We believe this hints that that there may be other factors leading to the increase in mobility near the vasculature beyond cardiac and respiration dynamics. This could be a membrane, but without anatomical confirmation, we will emphasize that this is a hypothesis.

New text in the discussion: "However, the presence of a membrane should still be considered a hypothesis until confirmed anatomically."

However, we do think that one of the key arguments for the presence of a membrane is the sharp drop off. We think that if there was no membrane present, then there would be a more diffuse fading of signal. We defined "sharp" in our methods as there being a local ≥20% change from the PVSAS to the SAS and the percentage of vessels with this sharp transition was 51% (Figure 7).

Additionally, we now quantitatively show the sharp drop-off with the post-M1 PVSAS with our automated concentric circles (Supplemental Figure 7, Supplemental Figure 8). On average, we see a drop off of signal around 1.5 mm (11 subjects, post-M1 $p=6.0\times10^{-4}$,

paired t-test, Bonferroni-corrected). Using this concentric circle analysis, we found that the majority of the post-M1 vasculature showed a ≥20% change of CSF-mobility from the max peak (Supplemental Figure 8).

We have added this additional text to the results section:

"Semi-automated concentric circles around the post-M1 vasculature mirror these results, showing high CSF-mobility with a steep drop-off at ~1.5 mm distance from the vessel wall (Supplemental Figure 7f; 11 subjects, post-M1 p=6.0x10-4, paired t-test, Bonferroni-corrected, 2 tests; see also Supplemental Figures 7f, 8). Mean non-motion sensitized CSF-signal show no significant change between where the PVSAS is expected (0-1.5 mm) and the SAS (1.5-3 mm) (Supplemental Figure 7h; 11 subjects, post-M1 p=0.74, paired t-test; see also Supplemental Figures 7g and 8)."

We have added additional text about the sharp drop off in our discussion:

"Additionally, the CSF-mobility PVSAS to SAS transition shows a sharp drop off (e.g. Figure 2a,b,c,e,f,g,i,j,k; Supplemental Figures 3a, 4b,f, 9i-k), which may indicate the presence of a membrane. This is corroborated quantitatively by steep drop-off profiles transitioning from the PVSAS to the SAS (Figures 2c,g,k; 4a,b) and by the concentric circle analysis of post-M1 vessels, which show drop-off at ~1.5 mm from the vessel wall (Supplemental Figures 7, 8). With the concentric circles, the majority of the post-M1 PVSAS examined showed a ≥20% drop-off from the maximum mobility. If elevated CSF-mobility near vessels arises solely from cardiac and respiratory pulsations, we would instead predict a gradual, rather than abrupt, decline with distance. Consistent with a membrane, intrathecal contrast scans[5] (Figure 2,d,h,l; Supplemental Figures 2c,d,g,h, 4a,b,e,f) shows that the contrast stays close to vessel for a prolonged time (e.g. MCA) and then diffuses. The increase in CSF-mobility within the PVSAS likely also explains the faster dispersion of intrathecal contrast agent within the PVSAS. However, the presence of a membrane should still be considered a hypothesis until confirmed anatomically."

Below are the figures mentioned above:

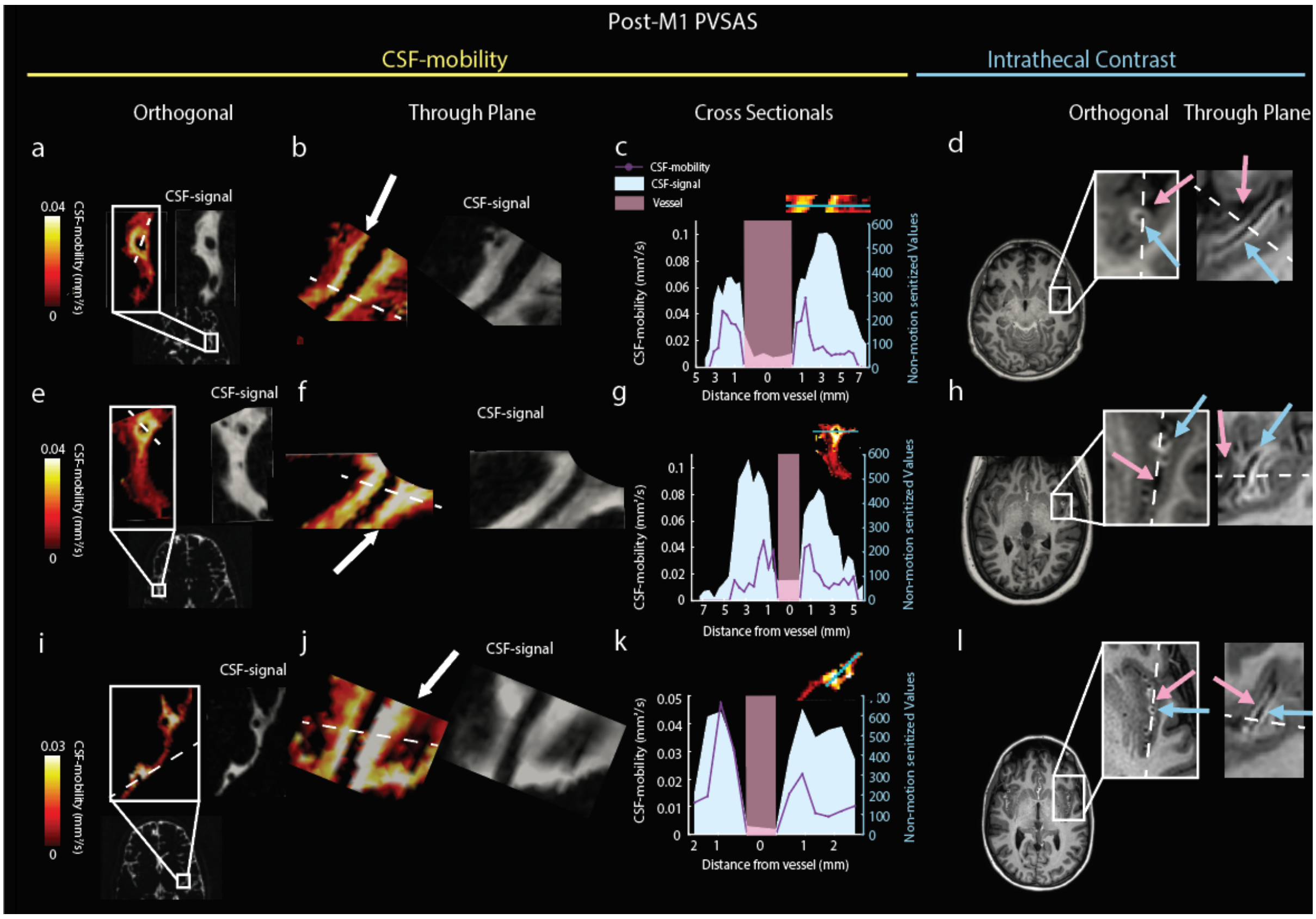

Figure 2 | Perivascular subarachnoid space (PVSAS) in the post-M1 (M2-M4) region.

a/e/i) *First column* shows full-donut PVSAS with high CSF-mobility surrounding post-M1 arteries. Non-motion-sensitized scans (gray) (referred to as 'CSF-signal') confirm equal CSF presence in both high- and low-mobility regions. Some of the PVSAS show some structural heterogeneity, indicating that the full-donut PVSAS can be asymmetric and deviate from perfect circularity (e.g., e, i). White boxes mark regions used for PVSAS extraction; dotted lines indicate locations of the corresponding through-plane images in second column.

b/f/j) *Second column* shows through-plane views of orthogonal CSF from the first column, displaying CSF-mobility, alongside matching non-motion-sensitized scans (gray). Dotted lines indicate locations of the corresponding orthogonal images in first column.

c/g/k) *Third column* presents representative cross-sectional plots from similar areas in the first and second columns. The pink bar marks the vessel; the x-axis shows distance from the vessel (mm), with CSF-mobility (purple, left y-axis) and non-motion-sensitized signal (blue, right y-axis). ROI location on the CSF-mobility map is indicated in the top right of the plot.

d/h/l) *Fourth column* shows post-M1 intrathecal contrast-enhanced PVSAS from patients from similar anatomical locations as in the first and second column (examples are from

data used in Eide & Ringstad, 2024). Dotted white lines indicate intersecting locations. Pink arrow highlights the subarachnoid space (SAS), and the blue arrows show the contrast within the PVSAS.

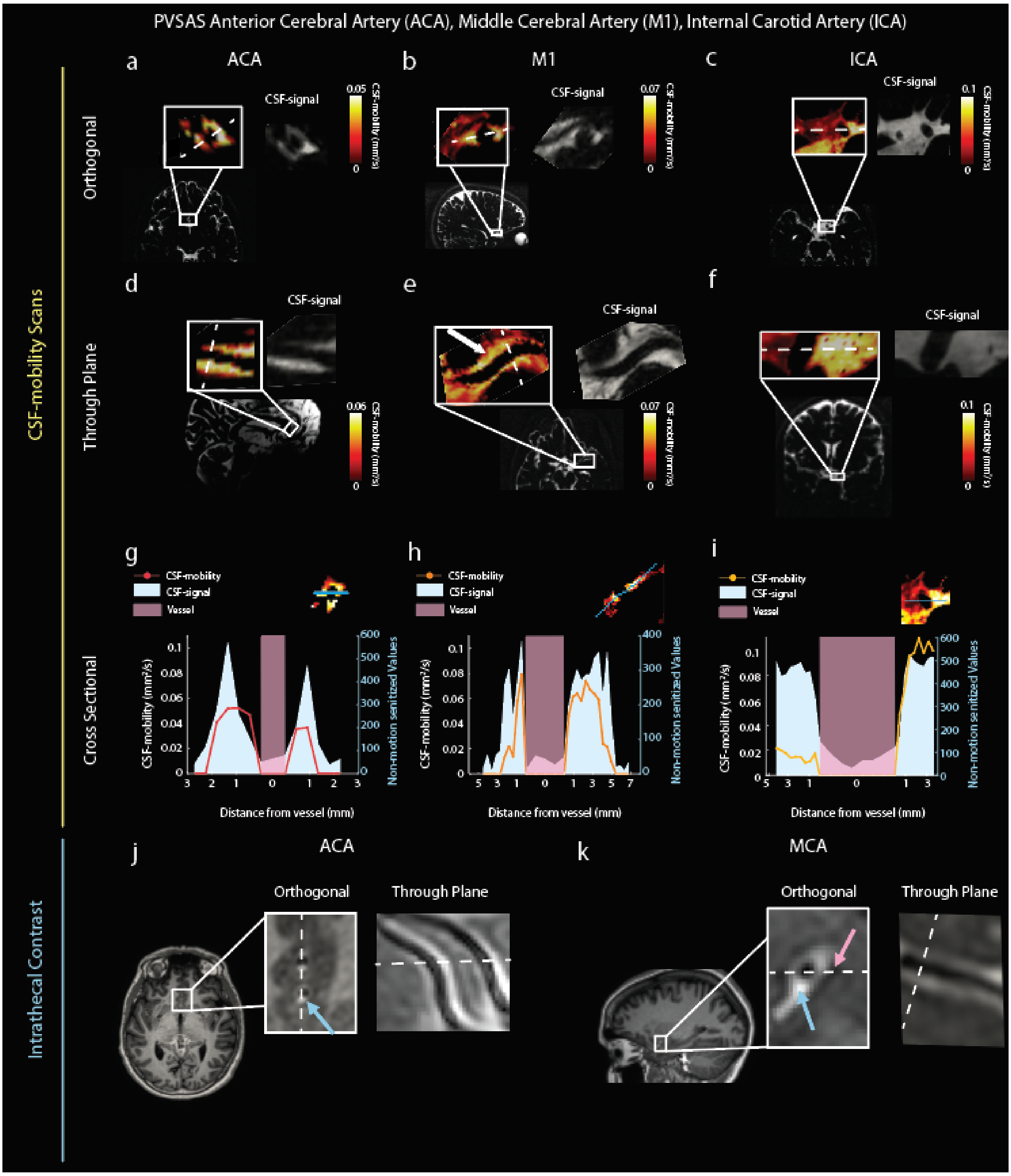

Figure 3 | Perivascular subarachnoid space (PVSAS) across the anterior carotid artery (ACA), M1 and internal carotid artery (ICA).

a) Example of an ACA CSF-mobility PVSAS, displaying tight-CSF in orthogonal image. The non-motion-sensitized scan (gray) (referred to as 'CSF-signal') shows a narrower CSF space around the vessel compared to other locations, for example around the MCA. The white box marks the extraction site of the PVSAS; the white dotted line indicates the location of the through-plane image seen in 'd'.

b) Example of an M1 CSF-mobility PVSAS showing high CSF-mobility near the artery in a bilateral triangular pattern within a region of lower CSF-mobility. The non-motion-sensitized scan (gray) confirms the presence of CSF in the zoomed-in view, indicating that reduced mobility is not due to a reduction in CSF signal. Identical layout to 'a'.

c) Example of an ICA CSF-mobility two-compartment, showing asymmetric mobility: low CSF-mobility on one side of the artery and high on the other. The non-motion-sensitized scan (gray) confirms similar CSF signal on both sides of the artery. White box indicates the extracted region for through-plane image.

d/e/f) Through plane images of ACA, M1, and ICA CSF-mobility. The non-motion-sensitized scan (gray) shows CSF presence. White dotted line shows location of orthogonal cut from 'a/b/c'.

g/h/i) Representative cross-sectional plots of the PVSAS shown in similar area to orthogonal cut for ACA, M1, and ICA above. Pink bar represents the vessel; the x-axis indicates distance from the vessel (mm), the left y-axis shows CSF-mobility, and the right y-axis shows non-motion-sensitized signal. ROI location is indicated in the corner.

j) ACA (A1) intrathecal contrast scan showing tight contrast-enhancement[5] near the vessel (blue arrow), and can be compared to the ACA PVSAS in 'a' and 'd'. White dotted line indicates where orthogonal and through-plane images were extracted.

k) MCA intrathecal contrast scan shows high eye-shaped contrast-enhancement[5] next to the vessel (blue arrow), within a lower contrast area (pink arrow), and can be compared to M1 PVSAS in 'b' and 'e'. White dotted line shows where the orthogonal and through-plane images were extracted.

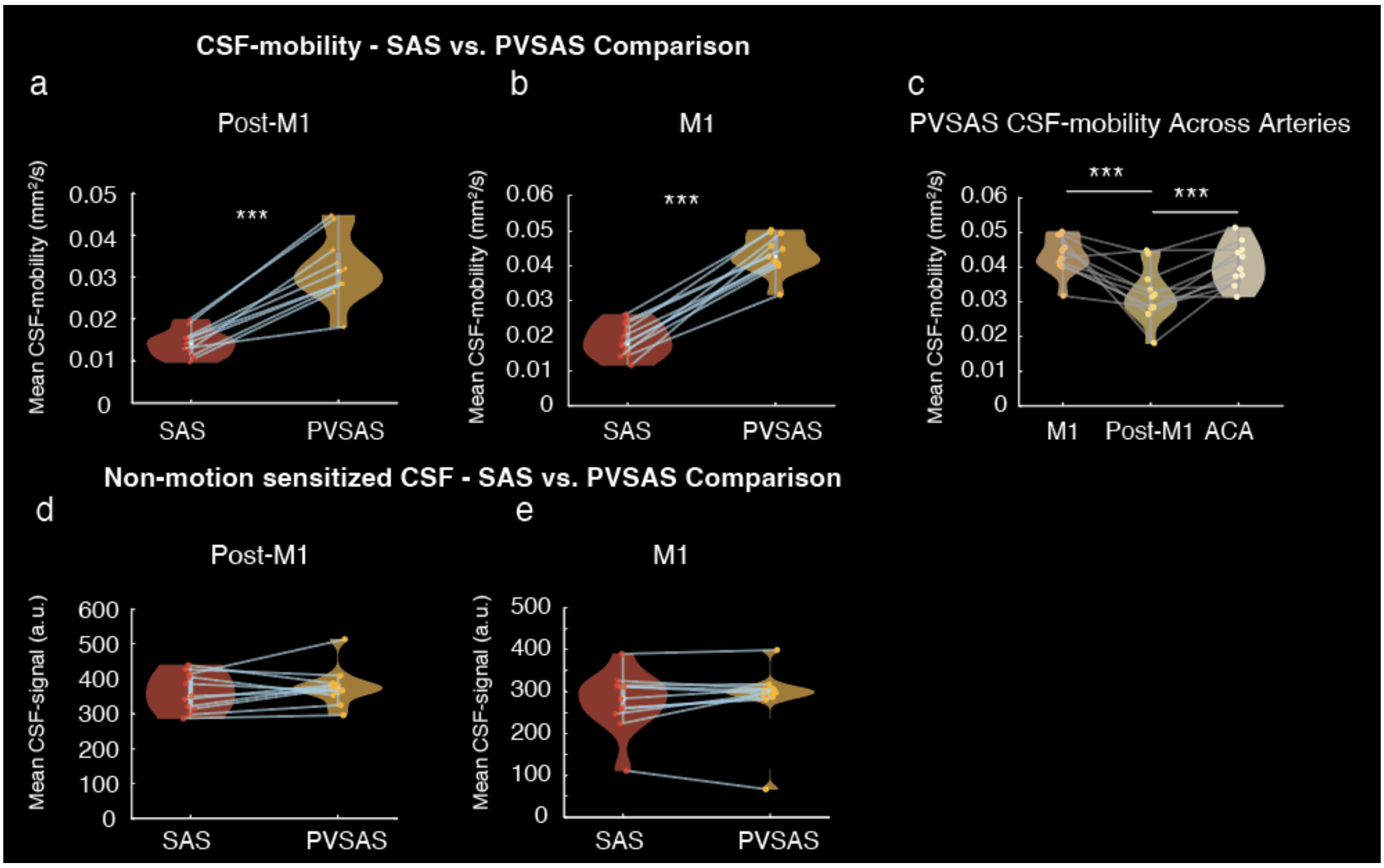

Figure 4 | Elevated CSF-mobility in the perivascular subarachnoid space (PVSAS) relative to the subarachnoid space (SAS).

a) Violin plot of post-M1 mean CSF-mobility in two subsections of the SAS and PVSAS (post-M1 PVSAS mean=0.032±0.008 mm^2/s, post-M1 SAS mean=0.01±0.003 mm^2/s; n=22 segments, 11 subjects, $p=2.0 \times 10^{-6}$, paired t-test, Bonferroni-corrected, 2 tests).

b) Violin plot of M1 mean CSF-mobility in two subsections of PVSAS and SAS (M1 PVSAS mean=0.043±0.005 mm^2/s, post-M1 SAS mean=0.02±0.005 mm^2/s; 22 segments, 11 subjects, $p=6.5 \times 10^{-8}$, paired t-test, Bonferroni-corrected, 2 tests).

c) PVSAS CSF-mobility values across M1, post-M1, and ACA, shows significantly more CSF-mobility in the M1 and ACA PVSAS than the post-M1 PVSAS (11 subjects, M1 vs. post-M1: $p=1.49 \times 10^{-3}$; M1 vs. ACA: p=0.192; post-M1 vs. ACA: $p=1.24 \times 10^{-3}$, paired t-test, Bonferroni-corrected, 3 tests).

d/e) SAS and PVSAS ROIs of the post-M1 and M1 on non-motion-sensitized images show similar CSF signal intensity, indicating that differences in CSF-mobility between SAS and PVSAS is not due to change in CSF presence (11 subjects, post-M1 p=0.32, M1 p=0.32, paired t-test).

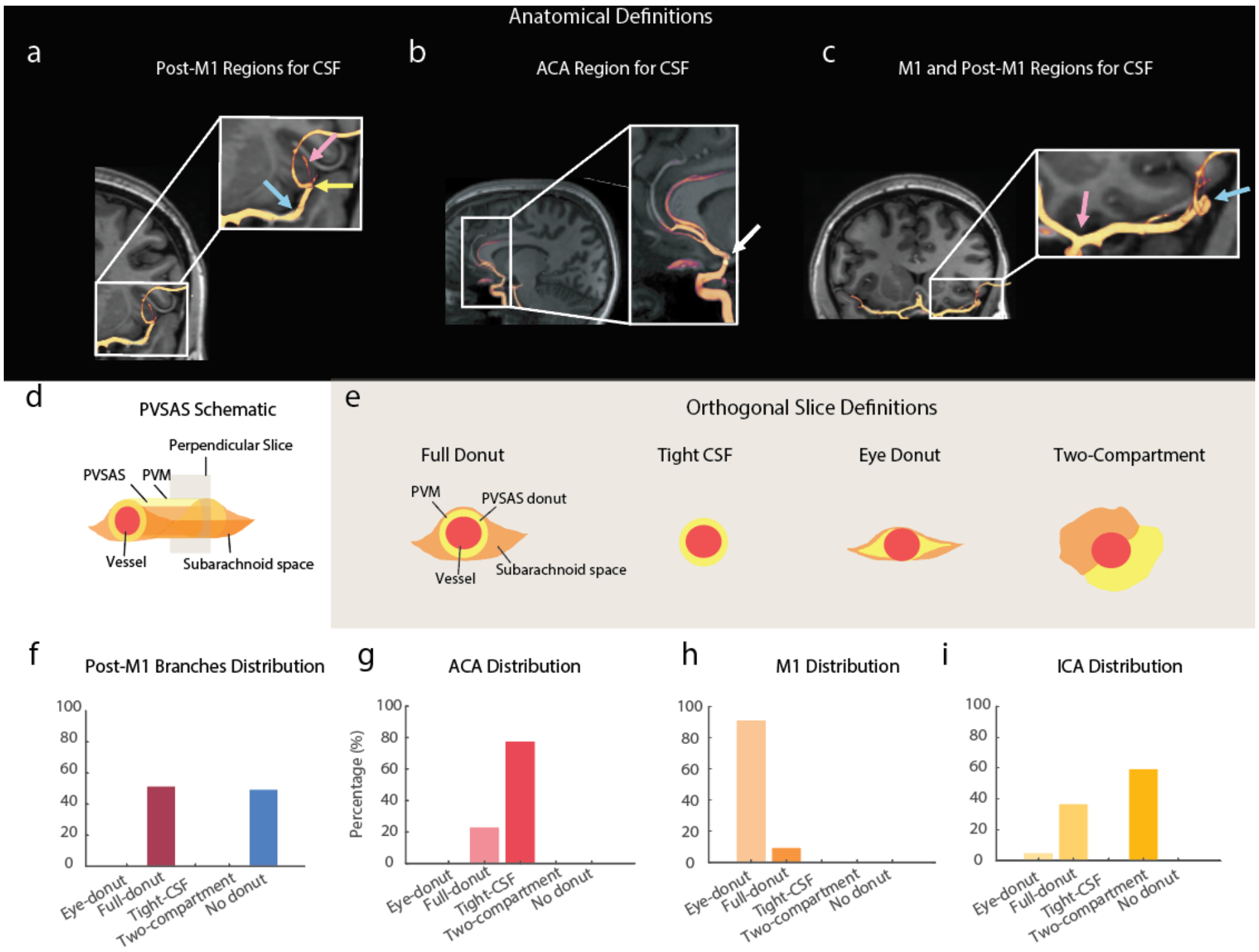

Figure 7 | Characterizing perivascular subarachnoid space (PVSAS).

a) 3D representation of arterial tree on top of a T1 anatomical transverse slice shows example of arteries that were rated in post-M1 distribution (pink arrow points to smaller artery). Blue arrow points to turn from M1 to M2. Yellow arrow points to turn from M2 to M3.
b) 3D representation of anterior carotid artery (ACA) on top of T1 anatomical sagittal image defines the ICA-to-ACA transition (white arrow).
c) 3D representation of middle cerebral artery (MCA) on top of T1 anatomical coronal image defines arterial transition ((internal carotid artery (ICA)-to-M1)) transition (pink), and M1-to-M2 transition (blue)).
d) Schematic illustration of perivascular subarachnoid space (PVSAS).
e) Cross-sectional schematics of CSF shapes.

f) Proportion of PVSAS located within the post-M1 arterial segments indicate 51% of visible arteries branching off the MCA have full-donuts (n=93 segments, 11 subjects).

g/h/i) Distributions of observed CSF shapes around ACA, M1, and ICA arteries (22 segments, 11 subjects).

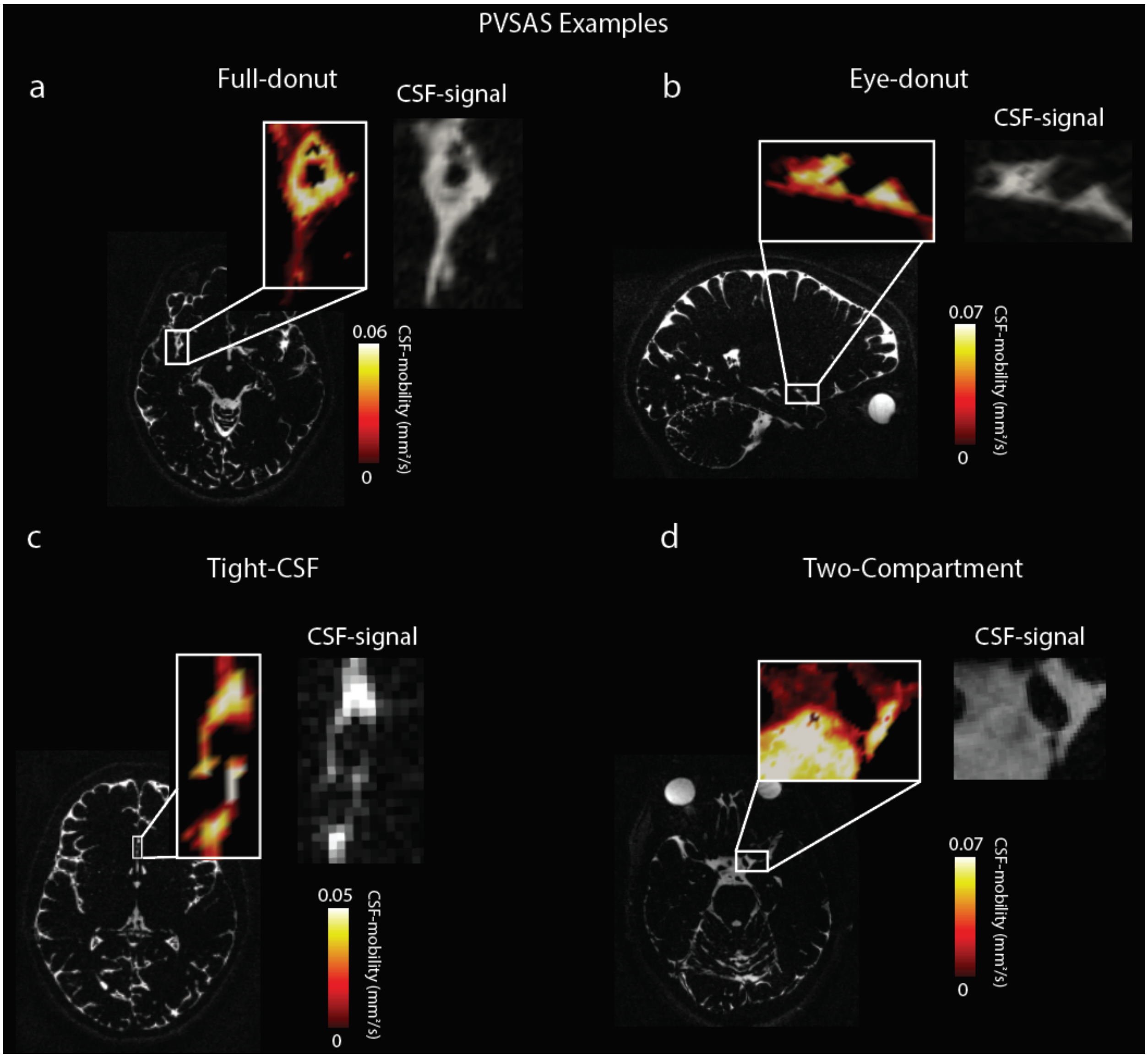

Supplemental Figure 3 | Examples of CSF structure across the anterior carotid artery (ACA), M1 and internal carotid artery (ICA).

a) Full-donut perivascular subarachnoid space (PVSAS).
b) Eye-donut PVSAS.
c) Tight-CSF.
d) Two-compartment.

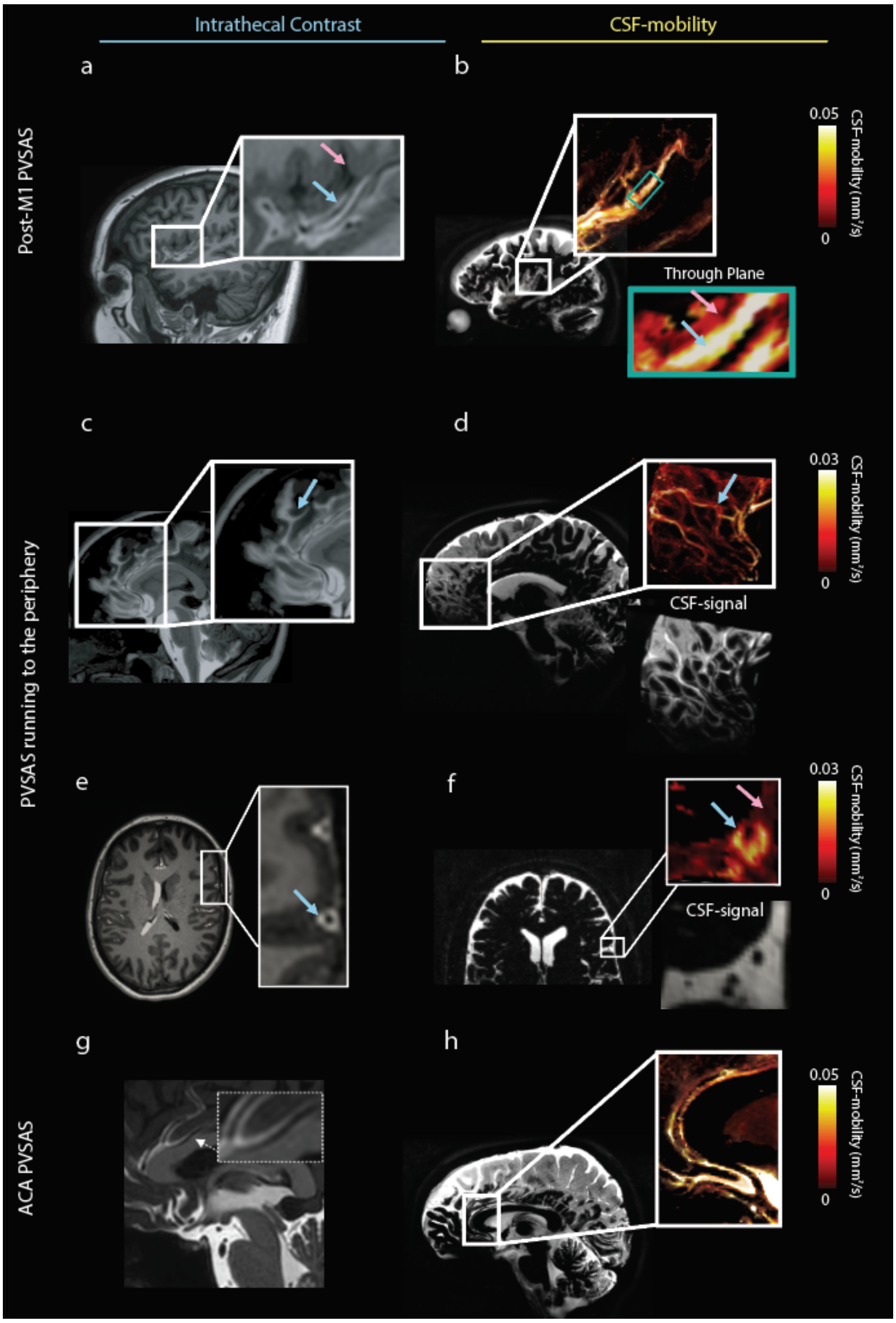

Supplemental Figure 4 | Intrathecal and perivascular subarachnoid space (PVSAS) comparisons.

a) Intrathecal contrast agent travelling along the post-M1 shows contrast in the perivascular subarachnoid space (PVSAS) close to the vessel (blue arrow), and no contrast in the subarachnoid space (SAS) (pink arrow).
b) CSF-mobility of the post-M1 PVSAS. Shown in gray is a maximum-intensity projection (MIP) of the non-motion sensitized scan, and in the white box is the CSF-mobility, showing the MIP of post-M1 PVSAS. Green box shows cross sectional of post-M1 PVSAS show steep drop off between the PVSAS (blue) and the SAS (pink).

c) Intrathecal contrast agent scan shows contrast running to the periphery in the PVSAS (blue arrow).
d) CSF-mobility, showing PVSAS running to the periphery. Gray MIP of non-motion sensitized scan.
e) Intrathecal contrast agent PVSAS running to the periphery (blue arrow).
f) CSF-mobility PVSAS at the periphery (blue arrow) surrounded by SAS (pink arrow).
g) Intrathecal contrast agent runs along anterior carotid artery (ACA) shows the PVSAS.
h) CSF-mobility 3D-representation of ACA shows limited SAS around the PVSAS.

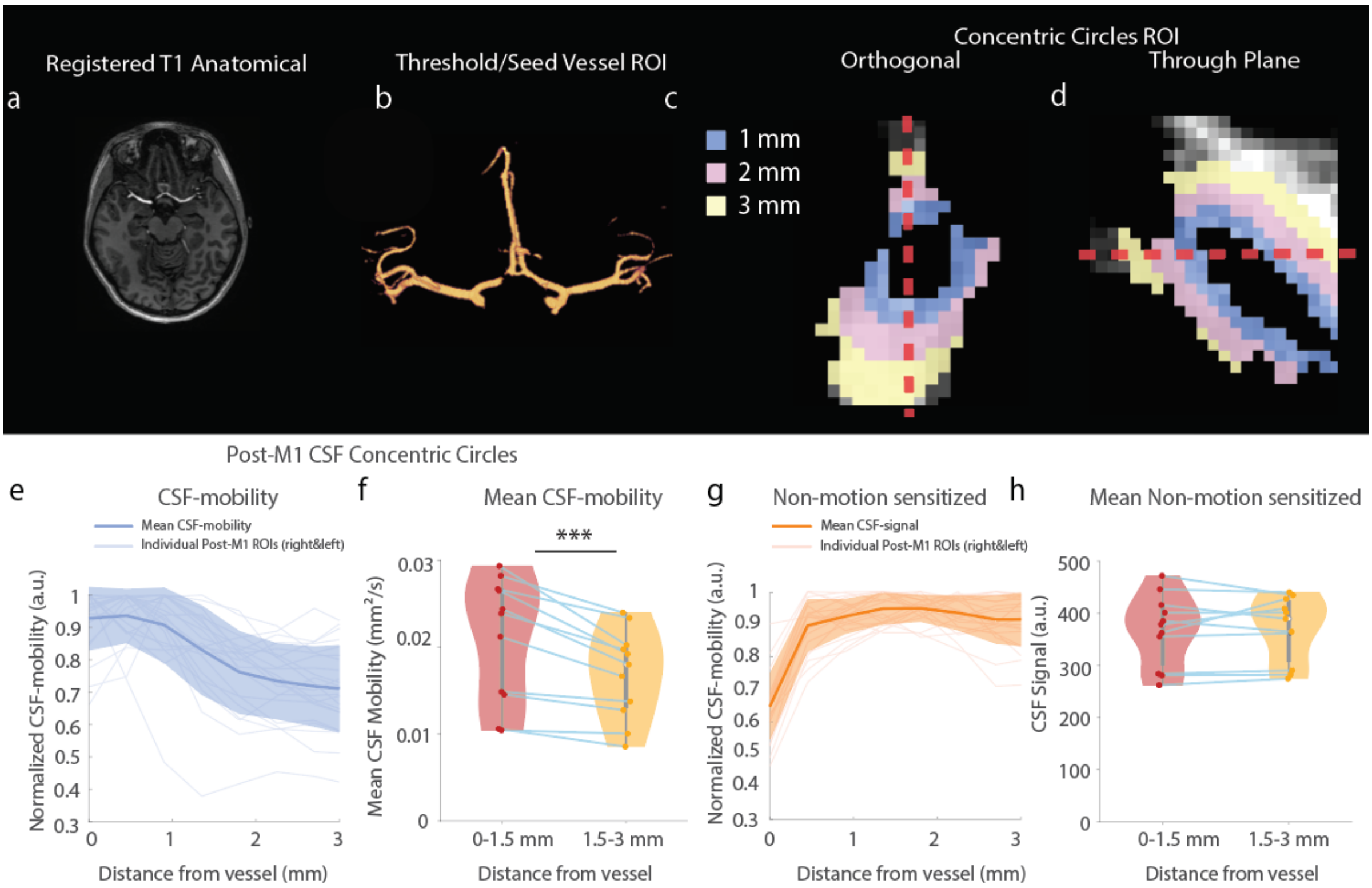

Supplemental Figure 7 | Concentric circles in the post-M1 region show distinct CSF-mobility in the PVSAS compared to the SAS, while CSF-signal remains similar.

a) Inflow effect visible in the registered T1 anatomical image used to create vessel mask.

b) Maximum intensity projection (MIP) of the vessel map after thresholding the T1 image and placing a seed at the ICA.

c/d) Concentric circles overlaid on the CSF-mobility scan representing distances of ~1 mm (blue), ~2 mm (pink), and ~3 mm (yellow) from the vessel wall. Red dotted line indicates corresponding orthogonal/through-plane cut.

e) Normalized CSF-mobility stepping away from the vessel shows high CSF-mobility with a steep drop-off at ~1.5 mm to low CSF-mobility. Light blue lines indicate individual post-M1 ROIs (right and left for each subject). Dark blue line indicates average. Shaded blue line shows standard deviation.

f) Mean CSF-mobility (0-1.5 mm vs. 1.5-3 mm) show significantly higher CSF-mobility where the PVSAS is expected (11 subjects, post-M1 $p=6.0 \times 10^{-4}$, paired t-test).

g) Normalized non-motion-sensitized CSF-signal as a function of distance from the vessel. A partial volume effect influences the CSF-signal close to the vessel independently of CSF-mobility, with no systematic decrease observed with increasing distance. Light orange lines indicate individual post-M1 ROIs (right and left for each subject). Dark orange line indicates average. Shaded orange line shows standard deviation.

h) Mean CSF-signal (0-1.5 mm vs. 1.5-3 mm) show no significant change in CSF-signal where the PVSAS is expected (11 subjects, post-M1 p=0.74, paired t-test).

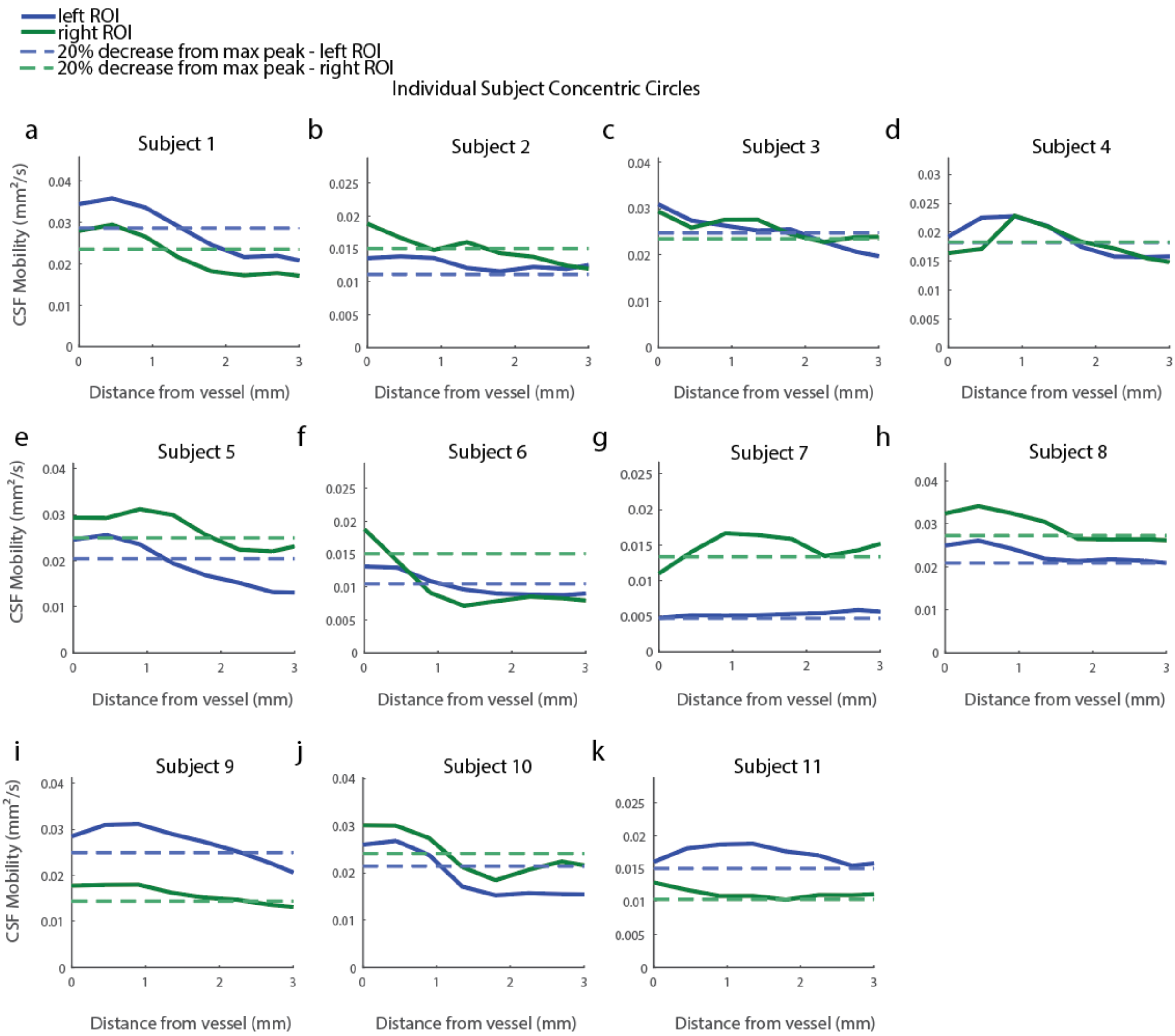

Supplemental Figure 8 | Individual subject concentric circles to visualize the perivascular subarachnoid space (PVSAS) in the post-M1 region. CSF mobility along concentric circles in the post-M1 perivascular subarachnoid space (PVSAS) for individual

subjects (a-k). Traces show mean CSF-mobility as a function of distance from the vessel wall for the left (blue) and right (green) hemispheres. Dashed lines indicate the 20% drop threshold from the peak CSF-mobility values for each hemisphere.

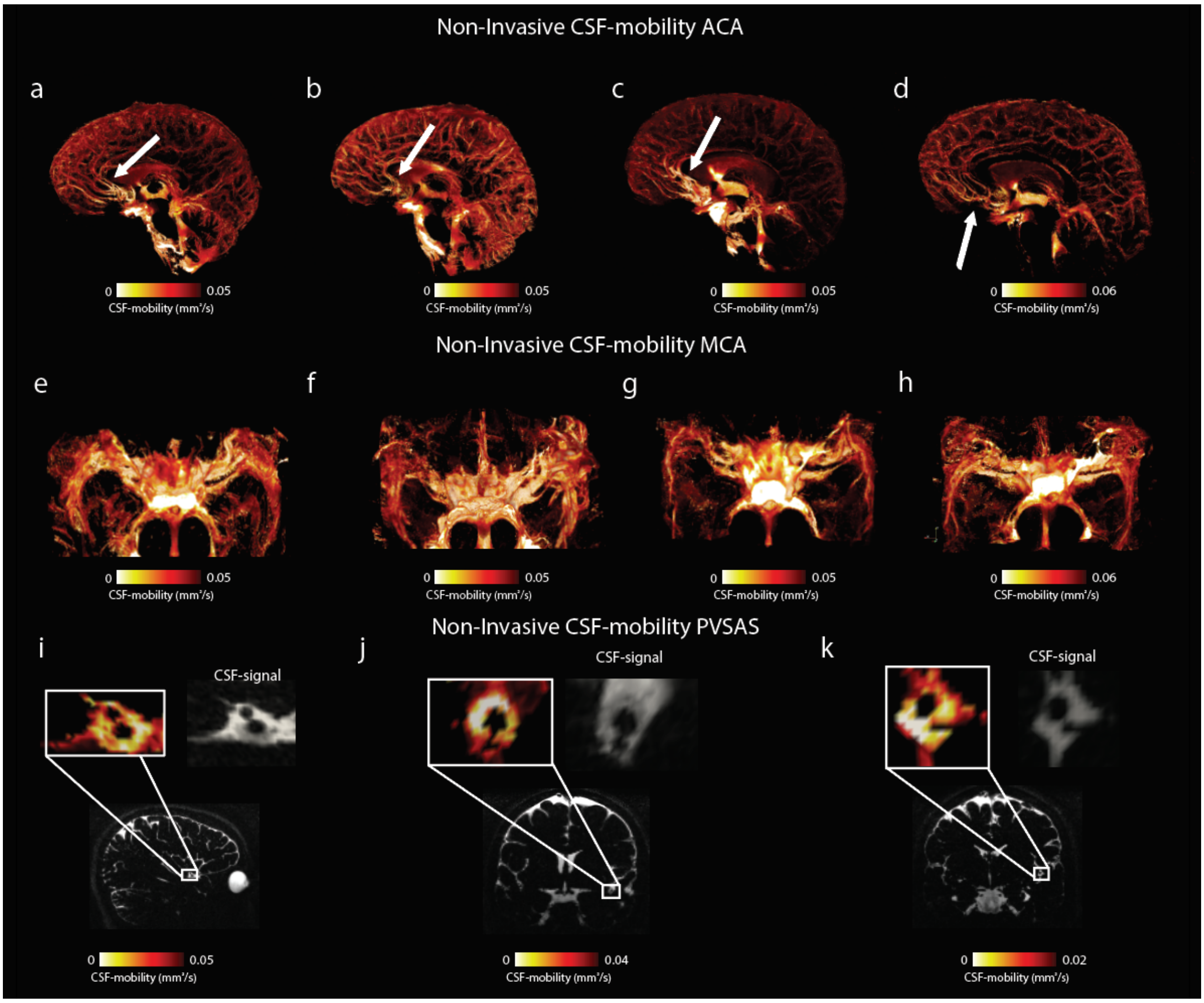

Supplemental Figure 9 | Additional examples of CSF-mobility perivascular subarachnoid space (PVSAS).

a-d) Additional sagittal views of CSF-mobility scan in healthy controls show perivascular subarachnoid space (PVSAS) surrounding the anterior carotid artery (ACA). White arrows highlight ACA.

e-h) Transverse views of CSF-mobility scan show high CSF-mobility PVSAS around the middle cerebral artery (MCA).

i-k) Examples of full-donut PVSAS with high CSF-mobility surrounding post-M1 arteries, next to matching non-motion-sensitized scans (gray). White boxes mark regions used for PVSAS extraction; dotted lines indicate locations of the corresponding through-plane images in second panel.

References

1. Hirschler, L. *et al.* Region-specific drivers of CSF mobility measured with MRI in humans. *Nat Neurosci* 1–10 (2025) doi:10.1038/s41593-025-02073-3.
2. Le Bihan, D. *et al.* MR imaging of intravoxel incoherent motions: application to diffusion and perfusion in neurologic disorders. *Radiology* **161**, 401–407 (1986).
3. Le Bihan, D. *et al.* Separation of diffusion and perfusion in intravoxel incoherent motion MR imaging. *Radiology* **168**, 497–505 (1988).
4. Helenius, J. *et al.* Diffusion-weighted MR imaging in normal human brains in various age groups. *AJNR Am J Neuroradiol* **23**, 194–199 (2002).
5. Eide, P. K. & Ringstad, G. Functional analysis of the human perivascular subarachnoid space. *Nat Commun* **15**, 2001 (2024).
6. Williamson, N. H., Komlosh, M. E., Benjamini, D. & Basser, P. J. Limits to flow detection in phase contrast MRI. *J Magn Reson Open* **2–3**, 100004 (2020).
7. Wen, Q. *et al.* Assessing pulsatile waveforms of paravascular cerebrospinal fluid dynamics using dynamic diffusion-weighted imaging (dDWI). *Neuroimage* **260**, 119464 (2022).
8. Bito, Y., Harada, K., Ochi, H. & Kudo, K. Low *b* -value diffusion tensor imaging for measuring pseudorandom flow of cerebrospinal fluid. *Magnetic Resonance in Med* **86**, 1369–1382 (2021).
9. Williams, S. D. *et al.* Neural activity induced by sensory stimulation can drive large-scale cerebrospinal fluid flow during wakefulness in humans. *PLoS Biol* **21**, e3002035 (2023).
10. Fultz, N. E. *et al.* Coupled electrophysiological, hemodynamic, and cerebrospinal fluid oscillations in human sleep. *Science* **366**, 628–631 (2019).

11. Debiasi, M., Van Osch, M. J. P., Dong, Y. & Hirschler, L. Highly-accelerated CSF-STREAM: two-fold faster CSF-mobility and FA measurement in PVS, via Locally Low Rank reconstruction. in 4744 (Honolulu, Hawaii, USA). doi:10.58530/2025/4744.
12. Prinse, F. A. M. *et al.* Investigating the role of neuroinflammation and brain clearance in frontotemporal lobar degeneration using 7T MRI and fluid biomarkers: protocol for a cross-sectional study in a tertiary care setting. *BMJ Open* **15**, e102668 (2025).
13. Kuhn-Keller, J. A. *et al.* The White Matter Hyperintensity Shape and Brain Clearance (WHIMAS) Study for Identification of Novel 7T Magnetic Resonance Imaging Markers of Cerebral Small Vessel Disease: Protocol for a Cross-Sectional Study. *JMIR Research Protocols* **14**, e77681 (2025).
14. Schriemer, S. E. *et al.* Stimulating amyloid-β clearance in cerebral amyloid angiopathy with low-sodium oxybate and/or non-invasive vagus nerve stimulation (Clear-Brain): study protocol for a randomised pre-post trial. *BMJ Open* **16**, e113194 (2026).
15. Plog, B. A. *et al.* A route for cerebrospinal fluid flow through leptomeningeal arterial–venous overlaps enables macromolecule and fluid shunting. *Nat Neurosci* 1–10 (2025) doi:10.1038/s41593-025-01977-4.
